# Advances in Topological Thermoelectrics: Harnessing Quantum Materials for Energy Applications

**DOI:** 10.1002/adma.202506417

**Published:** 2025-07-09

**Authors:** Guangsai Yang, Lina Sang, Chao Zhang, Khay See, Alex Hamilton, Michael Fuhrer, Ning Ye, G. Jeffrey Snyder, Xiaolin Wang

**Affiliations:** ^1^ State Key Laboratory of Crystal Materials Tianjin Key Laboratory of Functional Crystal Materials Institute of Functional Crystal Tianjin University of Technology Tianjin 300384 China; ^2^ Institute for Superconducting and Electronic Materials Faculty of Engineering and Information Science University of Wollongong, Innovation Campus Squires Way North Wollongong NSW 2500 Australia; ^3^ ARC Center of Excellence in Future Low Energy Electronics University of Wollongong Innovation Campus North Wollongong NSW 2500 Australia; ^4^ Department of Materials Science and Engineering Northwestern University Evanston IL 60208 USA; ^5^ School of Physics University of New South Wales Sydney NSW 2082 Australia; ^6^ School of Physics and Astronomy Monash University Clayton Victoria 3800 Australia; ^7^ CCTEG China Coal Research Institute No.5 Qingniangou East Road, Heping Street, Chaoyang District Beijing 100013 China

**Keywords:** Nernst effect, Seebeck effect, thermoelectric materials, topological materials

## Abstract

Thermoelectric (TE) effect, which enables the direct conversion of heat into electricity or vice versa, has great importance for condensed matter physics and material science due to its great potential for sustainable energy applications. Topological materials, with their topologically nontrivial band structures and rich physical phenomena, offer exciting opportunities for achieving efficient TE energy conversion. Here, an overview of the recent theoretical is provided and experimental advances at the intersection of topology and thermoelectricity. The unique features of topological materials are examined, such as band inversion, topological surface/edge states, linear Dirac/Weyl bands, and Berry curvature, affect their TE transport properties. Additionally, the potential of band topology to enhance both longitudinal and transverse TE performance is discussed. The current challenges and prospects for further advancing topological TE materials and devices are identified, aiming to develop high‐performance TE materials and devices.

## Graphical abstract

Topological materials offer a novel platform for thermoelectric energy conversion through unique band features, including band inversion, linear Dirac bands, surface states, and Berry curvature. These quantum characteristics enable enhanced longitudinal and transverse thermoelectric effects, establishing topological thermoelectrics as a promising frontier for both fundamental research and advanced energy technologies.

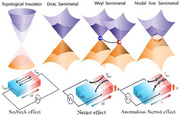

## 1 Introduction

TE technology has garnered significant interest as a sustainable, low‐emission alternative for future energy conversion, particularly in the areas of power generation, waste heat recovery, and thermal management.^[^ ^1^, ^2^, ^3^, ^4^, ^5^ ^]^ The heat‐to‐electricity conversion primally relies on the thermal, electrical, and thermomagnetic effects, such as Seebeck effects, Nernst effects and spin caloritronic effects. Over the past several decades, the field of conventional thermoelectrics based on the Seebeck effect has been undergoing successive development since the degenerate semiconductors have been recognized as good TE materials. The performance of a TE material is characterized by the dimensionless figure of merit, *zT* = *S* ^2^ *σT*/*κ*, where *T* is the temperature, *S* is the Seebeck coefficient, σ is the electrical conductivity, and *κ* is the thermal conductivity. Various strategies, including band engineering,^[^ ^6^, ^7^, ^8^, ^9^, ^10^, ^11^ ^]^ nanostructure engineering,^[^ ^12^, ^13^ ^]^ low‐dimensionality,^[^ ^14^, ^15^ ^]^ multiscale phonon scattering structures,^[^ ^16^, ^17^ ^]^ and energy filtering effect,^[^ ^18^ ^]^ have been employed to improve the performance of TE materials. However, the inherent incompatibility of properties in most proposed materials presents a significant challenge in achieving *zT* values required for practical, widespread applications. Therefore, the development of novel materials and the discovery of new physical mechanisms are essential to transcend conventional theoretical frameworks and drive further advancements in TE efficiency.

**Figure 1:**
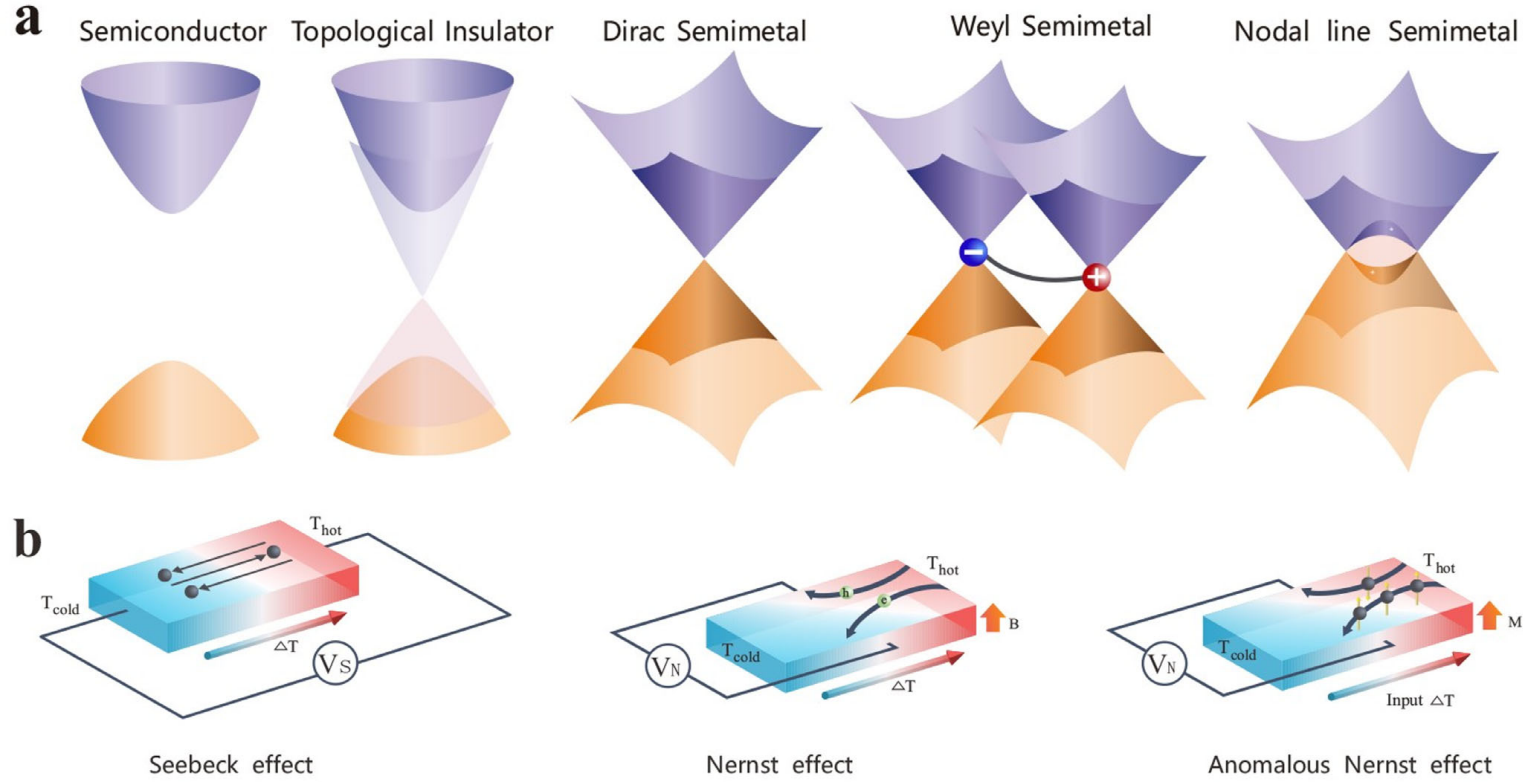
Band topology and thermoelectric effects. a) Schematic illustration of the band structures of trivial semiconductors and topological materials, including topological insulators, Dirac/Weyl semimetals, and nodal‐line semimetals. b) Corresponding longitudinal and transverse thermoelectric effects, including the Seebeck effect, the magneto‐Seebeck effect, the Nernst effect and the anomalous Nernst effect.

Over the past 18 years, the fields of quantum physics and material science have been revolutionized by the discovery of novel topological quantum phases, which have paved the way for advances in material design and their related applications. The concept of band topology has been extended to both electronic and phononic systems, giving rise to the emerging fields of “topological electronics”^[^ ^19^ ^]^ and “topological phononics”.^[^ ^20^ ^]^ which have paved the way for advances in material design and their related applications. According to topological band theory, solid materials can be reclassified as either topologically trivial or topologically non‐trivial.^[^ ^21^ ^]^ Topological materials (**Figure** **1**a), including topological insulators and topological semimetals, represent a new class of materials characterized by unique electronic structures, which include topologically protected surface or edge states, linear Dirac or Weyl bands, and large Berry curvature. These features confer unique properties to topological materials, such as ultra‐high carrier mobility and giant magnetoresistance, making them highly promising for a wide range of applications in dissipative power electronics, spintronics,^[^ ^22^ ^]^ catalysis,^[^ ^23^ ^]^ optics,^[^ ^24^ ^]^ and thermoelectricity.^[^ ^25^ ^]^

Interestingly, many topological materials have been identified in TE material system. For example, the well‐known room‐temperature TE materials, (Bi,Sb)_2_(Te,Se)_3_,^[^ ^26^ ^]^ are among the first generation of topological insulators(TIs), and several TIs exhibit excellent TE performance. Additionally, topological semimetals have been found to exhibit large longitudinal or transverse thermoelectric or magneto‐thermoelectric effects, including the Seebeck effect, Nernst effect and anomalous Nernst effect (Figure 1b). The intersection of topological materials and thermoelectrics has attracted intense research interest. A substantial body of theoretical and experimental works^[^ ^27^, ^28^, ^29^, ^30^ ^]^ has been dedicated to uncovering the influence of nontrivial topology on the TE transport properties. The principles of topological physics offer new directions beyond conventional TE research, enabling the development of highly efficient TE materials and devices for versatile energy harvesting applications by harnessing the diverse exotic electronic states of topological materials.^[^ ^31^, ^32^, ^33^, ^34^ ^]^

While some existing reviews^[^ ^35^, ^36^, ^37^ ^]^ have addressed the influence of topological surface states on TE transport properties. Here, we provide a comprehensive and up‐to‐date overview of the topological TE materials, highlighting their unique advantages and potential applications. We begin by reviewing recent theoretical research and experimental progress on TE transport phenomena in topological materials, emphasizing how their distinctive electronic structures influence TE transport properties. Then we delve into the large magneto‐thermoelectric effects observed in topological materials, discussing the pivotal role played by bulk band inversion, topological states, linear Dirac or Weyl bands, and Berry curvature in these systems. Furthermore, we explore the exciting potential of topological TE materials for advanced transverse power generation and cooling application. Finally, we address the current challenges and outline directions for further development.

## 2 Topological Materials

Topological materials are characterized by nontrivial topology in their band structures, a feature that manifests as robust surface/edge states and unconventional quantum phases distinct from those in conventional insulators, semimetals, or metals. Their defining property lies in topological invariants (e.g., Chern numbers, *Z*_2_ indices), which quantify the global geometric properties of electronic wavefunctions in momentum space.^[^ ^38^ ^]^ These invariants guarantee the existence of symmetry‐protected surface states—immune to smooth perturbations in material parameters—mirroring how a geometric object retains its topology under continuous deformations.^[^ ^39^ ^]^ In contrast, trivial materials lack such invariants and their bulk and surface states are not topologically protected. Based on band structure and symmetry considerations, topological materials are broadly categorized into topological insulators (gapless surface states with insulating bulk), topological semimetals (Dirac/Weyl nodes in bulk bands), and topological metals (nontrivial Fermi surface topology). These materials host exotic quantum phenomena that are absent in conventional systems, such as the quantum spin Hall effect and the chiral anomaly in electronic transport. The emergence of nontrivial band topology is primarily driven by band inversion between the conduction and valence bands, which arises due to the inert pair effect of heavy elements (resulting from atomic orbital interactions) and strong spin‐orbit coupling (SOC).

### 2.1 Topological Insulators

Topological insulators (TIs) are bulk insulators with a bandgap like trivial insulators, but they exhibit metallic surface states also known as topological surface states (Figure 1a). These surface states show a Dirac‐like linear dispersion and are occupied by massless fermions,^[^ ^40^ ^]^ which are topologically protected against backscattering by time reversal symmetry.^[^ ^41^, ^42^ ^]^ In addition to that, these states appear as a spin‐momentum‐locked helical metal, meaning the electron's spin is locked to its momentum direction. The topological invariant for TIs is the Z_2_ invariant, a mathematical quantity that serves as a critical criterion for distinguishing trivial insulators from TIs.^[^ ^43^ ^]^ The study of topological phases has primarily focused on materials with heavy elements, such as Bi_1−x_Sb_x_, strained α‐Sn, and HgTe. The first experimental confirmation of Dirac surface states in 3D TIs was observed in Bi_0.9_Sb_0.1_ using angle‐resolved photoemission spectroscopy (ARPES). Tetradymite compounds, including Bi_2_Se_3_, Bi_2_Te_3_, and Sb_2_Te_3_, are well‐known for their topological surface states and have been extensively studied for their tunable topological properties. In 3D TIs, all crystal surface facets have topological surface states. However, some systems only present topological properties on certain facets, while others remain insulating. These materials are classified as weak TIs examples of which include KHgSb,^[^ ^44^ ^]^ β‐Bi_4_I_4_,^[^ ^45^, ^46^ ^]^ and Bi_4_Br_2_I_2_.^[^ ^47^ ^]^ Furthermore, in TIs, surface states are protected by time‐reversal symmetry. In contrast, certain systems such as SnTe,^[^ ^48^ ^]^ Pb_1−x_Sn_x_Te,^[^ ^49^ ^]^ and Pb_1−x_Sn_x_Se^[^ ^50^ ^]^ have surface states protected by crystal symmetries, such as mirror and rotational symmetry, and are classified as topological crystalline insulators (TCIs). For example, SnTe exhibits robust surface states protected by the family of mirror planes, which only appear on crystal facets perpendicular to one of these planes.^[^ ^51^ ^]^

### 2.2 Topological Semimetals

Topological semimetals (TSMs) are materials that exhibit topologically stable band crossing in the bulk, either at discrete points or along the lines in the momentum space. This contrasts with trivial metals, which are characterized by the shape of their Fermi surface. The topological semimetals (Figure 1a) can be further divided into Dirac semimetals (DSMs), Weyl semimetals (WSMs), nodal line semimetals (NLSMs), and others depending on the crossing type.^[^ ^52^, ^53^ ^]^ In DSMs and WSMs, the band crossing occurs at special points in the Brillouin zone, known Weyl or Dirac points. DSMs have a four‐fold‐degenerate Dirac point with the same energy and momentum, whereas WSMs have a pair of two‐fold‐degenerate Weyl points with opposite chirality, resulting from the breaking of spatial inversion or time‐reversal symmetry. In WSMs, the opposite chirality of the Weyl nodes gives rise to a quantized chiral charge, akin to magnetic monopoles and anti‐monopoles in momentum space. A hallmark of WSMs is their topologically protected surface states, known as Fermi arcs, which connect the projections of Weyl nodes with opposite chirality on the material's surface. The TaAs family of compounds, including TaAs, TaP, and NbP, were among the first materials experimentally confirmed to be WSMs that break inversion symmetry. Co_3_Sn_2_S_2_ was later discovered as the first magnetic Weyl semimetal, with time‐reversal symmetry (TRS) breaking. In DSMs, where both reversal symmetry and inversion symmetry coexist, two doubly degenerate bands cross to form a Dirac node, with linear dispersion in all three momentum directions. Each Dirac node can be viewed as a pair of degenerate Weyl nodes of opposite chirality. Representative DSMs include Na_3_Bi and Cd_3_As_2_. In Nodal‐line semimetals, the band crossing can form a closed line or a ring in momentum space. The degeneracy of the nodal line can be twofold, as observed in the WSMs, or fourfold, as in the DSMs. It should be noted that the bulk Fermi surface in NLSMs consists of 1D lines, whereas in WSMs, it comprises 0D points. In addition to that, the surface of NLSMs features “drumhead”‐like surface states, in contrast to the 1D Fermi arc surface states shown in WSMs.

### 2.3 Topological Phonons

The concept of quantum topology has opened up new perspectives on phonon systems associated with lattice vibrations, leading to the development of a new field known as “topological phononics”.^[^ ^20^ ^]^ Unlike electronic systems, phonons obey Bose‐Einstein statics, meaning all phonon states across the phonon spectrum contribute to thermal transport and can be experimentally probed. In this section, we briefly outline the concept of topological phonons and their relevance to TE applications. In‐depth discussion, readers are referred to existing reviews ref. [20, 54]. Nontrivial topological phonon states in crystalline materials are theoretically classified into several distinct types,^[^ ^54^ ^]^ including topological optical phonons (e.g., Dirac/Weyl point phonons,^[^ ^55^, ^56^ ^]^ nodal‐line phonons,^[^ ^57^ ^]^ and nodal‐surface phonons),^[^ ^58^, ^59^ ^]^ topological acoustic phonons,^[^ ^60^ ^]^ and high‐order topological phonons.^[^ ^61^, ^62^ ^]^ The unique properties of topological phonons, like topology, Berry curvature, and Berry phase, provide an unusual way to control phonon transport, with implications for phenomena such as the phonon Hall effect,^[^ ^63^ ^]^ heat transport, and thermoelectricity.^[^ ^64^ ^]^ However, the impact of phonon topology on thermal transport remains poorly understood, and systematic research in this area is still limited. Several studies have explored the influence of disorder on phonon transport in topological systems. Ong et al. explored the influence of disorder on the transport and localization of phonon modes in a topological phononic lattice, showing that while topologically protected edge modes are highly robust against uncorrelated disorder, they diminish under spatially correlated disorder.^[^ ^65^ ^]^ Similarly, Ding et al. theoretically demonstrated that topologically protected phonon edge modes can scatter when bulk phonon modes provide backscattering channels within local bandgaps.^[^ ^66^ ^]^ Another theoretical study revealed that certain metallic compounds, such as TaSb and TaBi, exhibit phonon spectra characterized by the crossing of three phonon modes (two optical and one acoustic) at specific points in the Brillouin zone.^[^ ^64^, ^67^ ^]^ This topological bosonic excitation enhances phonon scattering, thereby reducing lattice thermal conductivity and imparting a phonon‐glass‐like behavior to these materials.^[^ ^64^ ^]^ Moreover, the topological fermionic quasiparticles present in these materials enhance electronic transport, making them “electron‐crystal” materials with a pronounced TE response.^[^ ^64^ ^]^ First‐principles calculations have also shown that Heusler compound Li₂CaPb exhibits exceptionally low lattice thermal conductivity due to Dirac‐like band crossings in its optical phonon branches, which possess nontrivial topological properties.^[^ ^68^ ^]^ Additionally, strain‐driven topological phonon phase transitions in hexagonal TiO have been found to significantly reduce lattice thermal conductivity by up to 78% at room temperature. This reduction is primarily attributed to increased phonon scattering rates, which arise from the breaking of symmetry‐protected degeneracies in phonon branches.^[^ ^69^ ^]^ These findings suggest that the interplay between phonon and electronic topology could offer new strategies for controlling heat and electron transport in solids, potentially leading to high TE conversion efficiency.

### 2.4 Topological Insulators as Thermoelectric Materials

Numerous high‐performance TE materials have been identified among TIs. Notably, some of the most effective room‐ or low‐temperature TE materials, such as (Bi,Sb)₂(Te,Se)₃ and Bi_1‐x_Sb_x_, represent the first generation of 3D TIs. The intersection of these excellent TE materials and TIs can be attributed to their shared traits, such as heavy elements and narrow bulk bandgaps. For TIs, bulk band inversion is essential for the emergence of topological surface states.^[^ ^70^ ^]^ This band inversion is driven by strong spin‐orbit coupling SOC (also known as spin‐orbit interaction (SOI)) or atomic orbital interactions (e.g., s‐p coupling),^[^ ^71^, ^72^ ^]^ which are typically observed in materials containing heavy elements. For TE materials, heavy elements contribute to weaker bonding, resulting in lower phonon thermal conductivity (*κ*_L_) and a narrow bandgap that facilitates σ and *S*. The potential of TIs as promising TE materials arises from their ability to fulfill the criteria for “phonon‐glass‐electron crystal” properties. Both theoretical and experimental research continue to emphasize the potential of TIs in the development of high‐efficiency TE materials. For example, good TE properties have been experimentally demonstrated in some well‐known TIs, such as Bi_1.1_Sb_0.9_Te_2_S,^[^ ^73^ ^]^ BiSe,^[^ ^74^ ^]^ and BiTe.^[^ ^75^ ^]^

Significant advancements have been achieved in understanding non‐conventional TE phenomena in TIs. Specifically, the role of topological surface states in TE transport has been comprehensively examined. Nevertheless, conclusive experimental evidence for their effectiveness in enhancing TE performance remains elusive.  This ambiguity arises, at least in part, from practical limitations inherent to TE devices, including macroscopic dimensions (millimeter‐scale or larger), operation above room temperature, and carrier concentrations exceeding 10¹⁹cm⁻^3^. Under these conditions, bulk transport mechanisms dominate over surface contributions. Consequently, bulk band structure features—such as bulk band inversion—are likely to play a critical role in determining bulk TE properties. However, the effect of bulk states on TE transport properties remains underexplored. Recent emerging studies now emphasize that bulk TI states—fundamentally driven by band inversion—can also significantly impact unconventional transport phenomena. In this section, we will discuss the unique TE effects originating from both bulk and surface states of TIs.

### 2.5 Impact of Bulk Band Inversion on Thermoelectric Properties

Enhancing conventional TE performance involves the simultaneous optimization of *S*, σ, and *κ*. However, these parameters are often interdependent, leading to inherent trade‐offs, particularly between high *σ* and large *S*. Increasing carrier concentration would naturally boost σ but reduce *S*. High‐dispersive or “light” bands, characterized by small effective mass and high carrier mobility, are favorable for *σ*. In contrast, low‐dispersive or “heavy” bands, with large effective mass and low mobility, are ideal for achieving large thermopower. This inverse relationship between band curvature in light and heavy bands highlights the challenge of optimizing TE performance. It emphasizes that improving TE efficiency involves more than just enhancing a single property; a balanced approach is essential to achieve optimal results.

**Figure 2:**
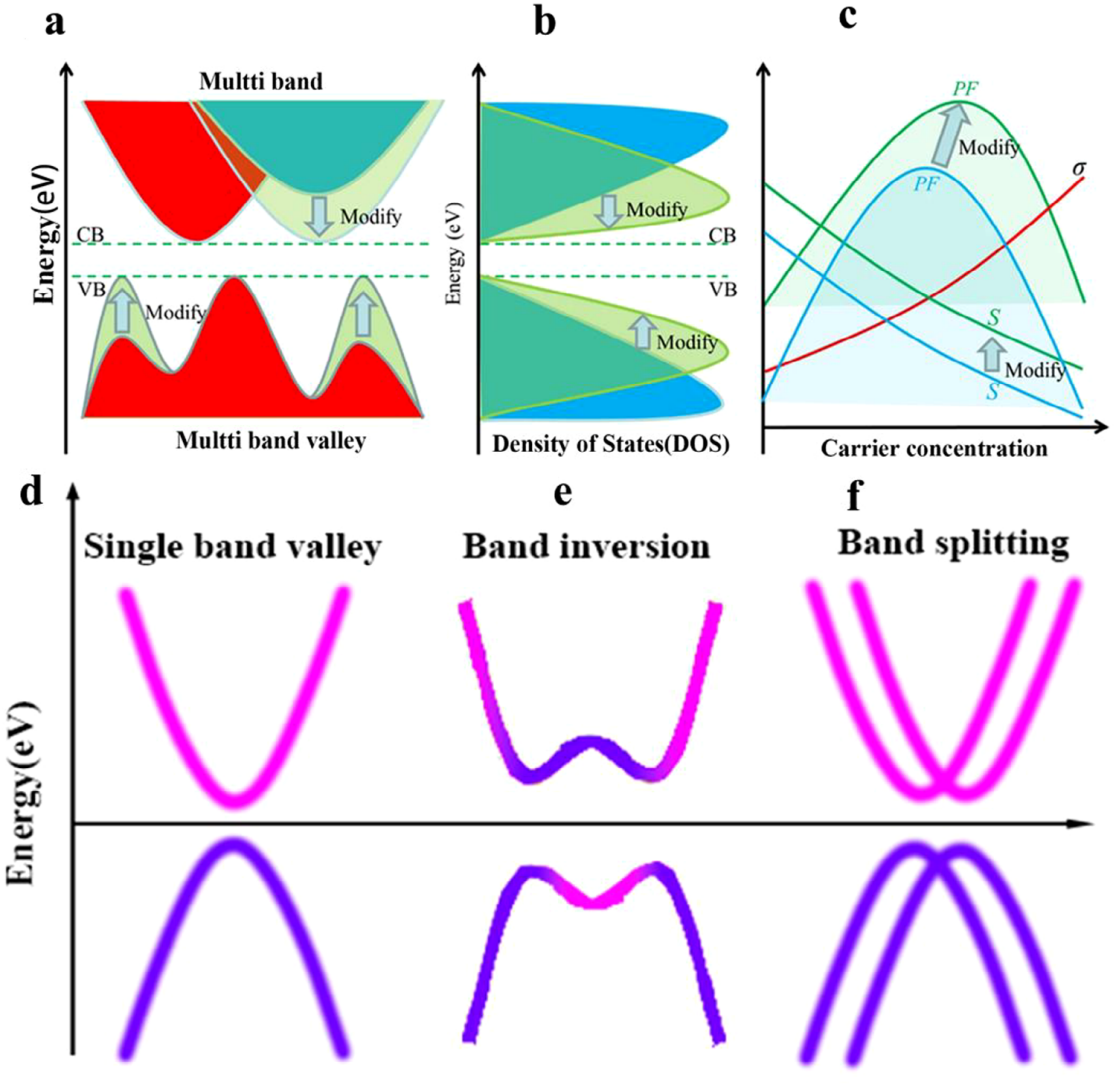
Band valley degeneracy. a) Schematic illustration of multi‐valley band degeneracy achieved by aligning different bands at the same energy level. b) Modification of the density of states (DOS) and power factor (c) resulting from band valley degeneracy. a–c) Adapted by with permission from^[^ ^76^ ^]^ under a Creative Common CC BY licence. d) Schematic of a single band valley. e) Schematic representation of bulk band inversion‐induced valley degeneracy. f) Schematic of valley degeneracy arising from band splitting.

One promising strategy to partially decouple *S* and σ is band convergence, which aligns different bands at distinct symmetry points to the same energy level (**Figure** **2**a). At a given Fermi level, such multiple band alignments increase both charge carrier concentration and density of states (DOS).^[^ ^76^ ^]^ The DOS effective mass, $m_{d}^{*}$, is given by $m_{d}^{*}={N_{v}}^{2/3}m_{b}^{*}$, where $m_{b}^{*}$ is the single‐pocket DOS effective mass, and *N*_v_ represents the number of Fermi surface pockets. Thus, a higher *N*_v_ enhances σ while maintaining *S*, thereby improving the power factor (*PF* = *S* ^2^ *σ*) as demonstrated in Figure 2b,c.

**Figure 3:**
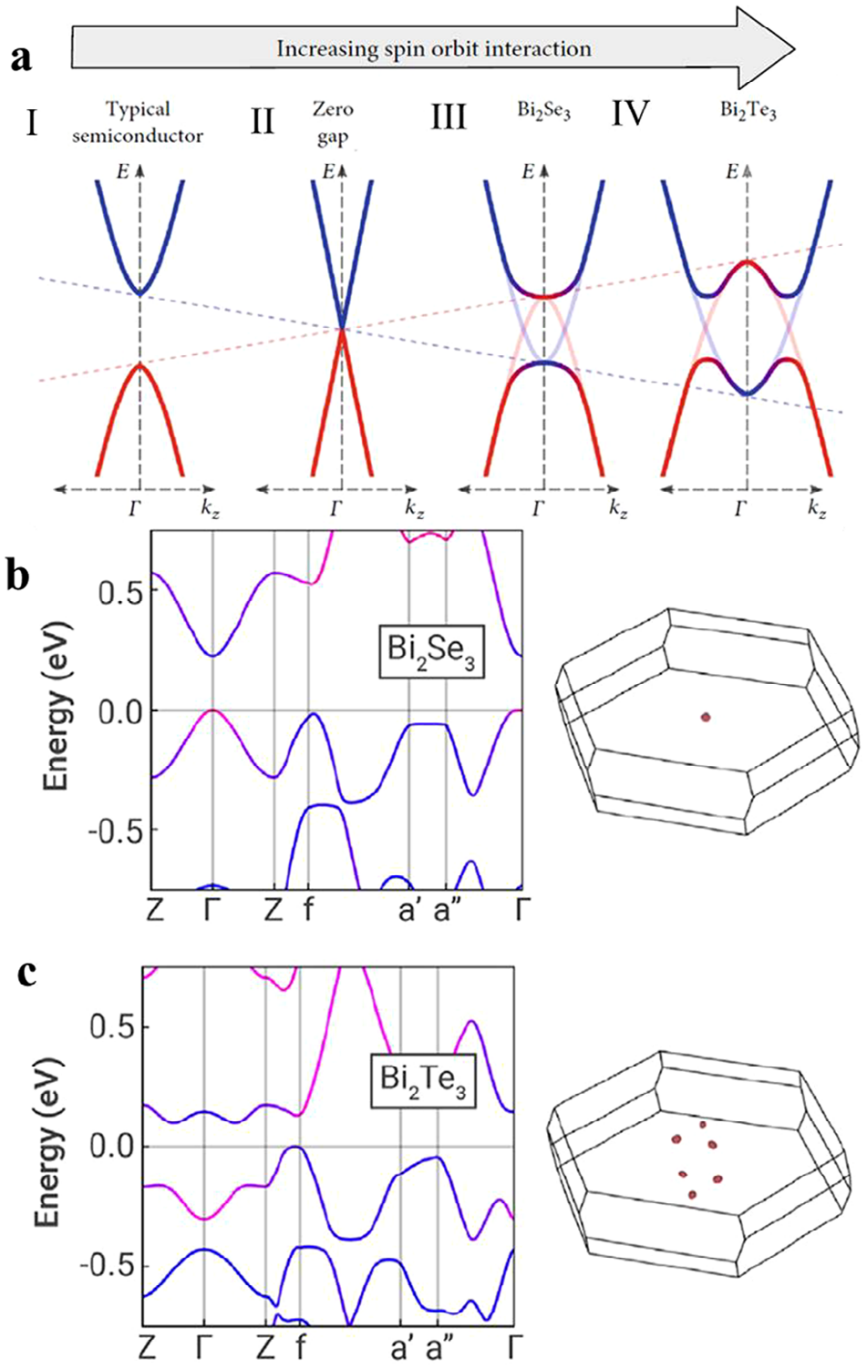
Bulk band inversion and valley degeneracy in Bi_2_Te_3_/Bi_2_Se_3_ topological insulators. a) In the absence of spin‐orbit interaction (SOI), bulk Bi₂Se₃ and Bi₂Te₃ behave as conventional semiconductors. As SOI increases, the bandgap gradually closes, eventually leading to band overlap. The SOI‐induced anticrossing at the overlap reopens the gap, reconfiguring the band states at the Γ point. a) Adapated with permission from^[^ ^79^ ^]^ under a Creative Commo CC BY licence. b) Band structure of Bi₂Se₃: The bands exhibit minimal inversion, resulting in a single valley (N_v_ = 1). c) Band structure of Bi₂Te₃: Sufficient band inversion leads to warped bands and enhanced valley degeneracy (N_v_ = 6). b,c) Adapted with permission from^[^ ^80^ ^]^ under a Creative Common CC BY licence.^[^ ^78^ ^]^

Bulk TIs exhibit a highly dispersive bulk band structure, resulting in a small carrier effective mass and high carrier mobility.^[^ ^77^ ^]^ More importantly, relativistic effects and strong SOC not only drive band inversion but also open a small energy gap. In single‐band systems, band inversion and band splitting can lead to valley degeneracy (Figure 2d–f). Notably, band inversion is a prerequisite for the emergence of topological surface states. When band inversion occurs at a specific k‐point (k_0_), strong band coupling can shift the band edges away from k_0_, giving rise to multiple carrier pockets. Snyder et al. demonstrated that the strength of band inversion directly influences the TE performance of bulk TIs.^[^ ^78^, ^79^ ^]^ **Figure** **3** presents a schematic illustration of how bulk band inversion drives valley degeneracy in the Bi₂Se₃‐Bi₂Te₃ system. In the absence of SOC, Bi₂Te₃ and Bi₂Se₃ exhibit a conventional bulk semiconductor band structure, where the conduction band minimum and valence band maximum form a single valley at the Γ point (Figure 3a(I)). As SOC increases, the bandgap gradually closes, eventually resulting in band overlap (Figure 3a,(II)). This SOC‐induced ant‐crossing at the overlap causes a gap reopening, reconfiguring the band states at the Γ point. Unlike Bi₂Se₃ (Figure 3a,(III) and Figure 3b), which exhibits single valley degeneracy (*N*_v_ = 1) due to its non‐warped (“U”‐like) band structure, Bi₂Te₃, with stronger SOC, shows significant band inversion. This results in warped, non‐parabolic bands (“W” or “M”‐like) near the Γ point (as shown in Figure 3a,(IV),c), leading to higher valley degeneracy (*N*_v_ = 6), which underpins its superior TE performance.

**Figure 4:**
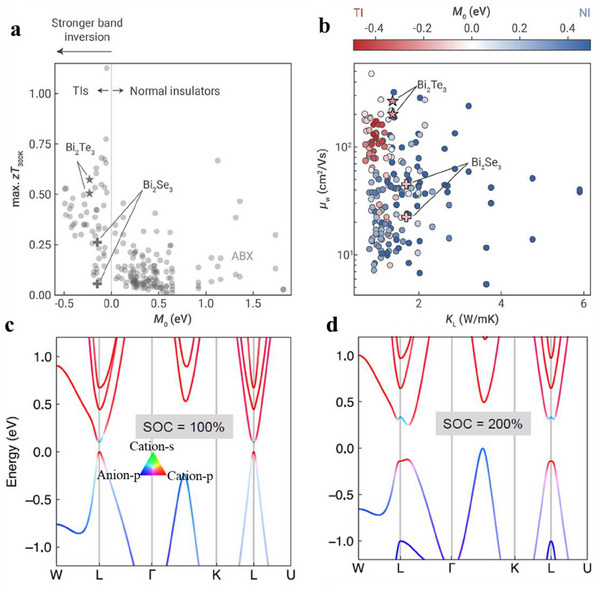
The effect of bulk band inversion on thermoelectric properties. a) The maximum attainable *zT* (optimized with respect to doping levels) at room temperature is calculated using first‐principles Boltzmann transport calculations. The maximum zT is plotted as a function of the M₀ parameter (defined in Equation 1), which represents the strength of band inversion for topological insulators (TIs). a,b) Adapted by with permission from^[^ ^78^ ^]^ under Attribution‐NonCommercial 3.0 Unported Licence. b) The weighted mobility (µ_w_) and lattice thermal conductivity (κ_L_) are shown, with TIs represented by red (negative M₀) and normal insulators (NIs) represented by blue (positive M₀). c) The electronic structure of SnTe when the SOC strength is set to (A) 100%, the normal amount, and (b) 200%. The SOC strength contributes to the degree to which bands are inverted. c,d) Adapted by with permission from^[^ ^81^ ^]^ under a Creative Commons CC‐BY license.

Therefore, enhancing the band inversion strength in bulk TIs could serve as a viable strategy for enhancing TE performance. A recently developed **k p** perturbation theory‐based model has provided a mathematical criterion for band warping in centrosymmetric TIs, characterizing band as:^[^ ^76^ ^]^

(1) $$|M_{0}|=\frac{E_{\mathrm{CB}}\left(k_{0}\right)-E_{\mathrm{VB}}\left(k_{0}\right)}{2}$$

where *E*_CB_ and *E*_VB_ represent the conduction and valence band edges, respectively, and k_0_ is the k‐point where band inversion occurs. The parameter |*M*_0_| quantifies the bandgap at k_0_, with negative M_0_ indicating band inversion in TIs and more negative values correspond to stronger inversion. In contrast, normal insulators, lacking inversion, have a positive *M_0_*. Band inversion‐driven warping explains the distinct band structures of Bi₂Se₃ and Bi₂Te₃, where the latter exhibits stronger inversion (i.e., more negative *M*_0_) due to enhanced SOC.^[^ ^81^ ^]^ A recent study investigating the impact of band inversion‐driven warping on TE performance, using first‐principles Boltzmann transport calculations, revealed that TIs generally achieve higher maximum *zT* values compared to conventional semiconductors.^[^ ^78^ ^]^ This enhancement is even more pronounced in TIs with stronger band inversion (**Figure** **4**a). The maximum attainable *zT* of a material is closely linked to the TE quality factor*B* = μ_*w*_/κ_*L*_, where *µ*_w​_ is the weighted mobility (carrier mobility weighted by the density of states). Higher values of *B* lead to higher maximum *zT*. Band inversion is typically induced by the strong SOC, which is associated with heavy elements; thus, they are expected to have low *κ*_L_. Moreover, the band warping in a TI gives rise to high *µ*_w_ as revealed by first‐principles calculations in Figure 4b.

Band inversion is primarily governed by SOC and atomic orbital interactions, making it tunable through adjustments to these interactions. For instance, in the case of SnTe (Figure 4c), the band extrema are located at the L‐point. Doubling the strength of the SOC in SnTe theoretically leads to a greater separation between the valence and conduction band edges at the L‐point, potentially even causing an off‐centering of the band edges along the L‐Γ direction (Figure 4d).^[^ ^81^ ^]^ This effect signifies an intensified band inversion. Snyder et al. have provided critical insights into band inversion‐driven warping and valley degeneracy in centrosymmetric, non‐magnetic TIs.^[^ ^81^ ^]^ Their findings highlight two essential conditions for valley degeneracy: significant band inversion and the presence of energetically proximate secondary bands with opposite parity near the band edges. Remarkably, their study mapped the potential valley degeneracies across 11 centrosymmetric point groups, revealing the possibility of increasing valley degeneracy by up to 48‐fold. These insights establish a comprehensive framework for exploiting the non‐trivial electronic structures of TIs, paving the way for advanced band structure engineering.

Bulk band inversion can be enhanced through mechanical strain, external pressure, or alloying. First‐principles calculations reveal that hydrostatic compression strengthens band inversion in Bi₂Te₃, leading to an increased maximum *zT* at room temperature for both n‐ and p‐type carriers.^[^ ^78^ ^]^ This effect arises from increased cation‐p/anion‐p hybridization and crystal field splitting in the presence of compression strain, which lower the Bi‐p_z_ state relative to the Te‐p_z_ state, making M_0_ more negative.^[^ ^80^, ^82^ ^]^ Experimentally, external pressure enhances the power factor in Bi_0.5_Sb_1.5_Te_3_, as evidenced by the increase in *µ*_w_ with pressure.^[^ ^83^ ^]^ Alloying is another effective strategy for tuning the band structure and optimizing transport properties. For example, the metastable rock‐salt SnSe, a known TI with a highly warped band structure, shows an enhanced power factor and increased *µ*_w_ upon AgSbSe_2_ alloying.^[^ ^84^ ^]^ This enhancement is attributed to compressive strain, evidenced by reduced lattice parameters, which strengthens sp‐mixing and intensifies band inversion, further warping the band structure. In the SnTe system, co‐alloying SnTe with GeTe and PbTe induces band inversion, generating multiple valence band maxima with small energy gaps (≈0.03–0.04 eV). This enables Fermi level crossing of multiple valleys under high hole doping, enhancing the *S* more than it reduces σ, thereby improving TE performance. Therefore, inducing band inversion through alloying offers a promising route to creating multiple electronic valleys in materials that originally possess a single band, ultimately leading to significantly enhanced TE performance.

### 2.6 Enhanced Thermoelectric Performance via Electronic Topological Transitions

**Figure 5:**
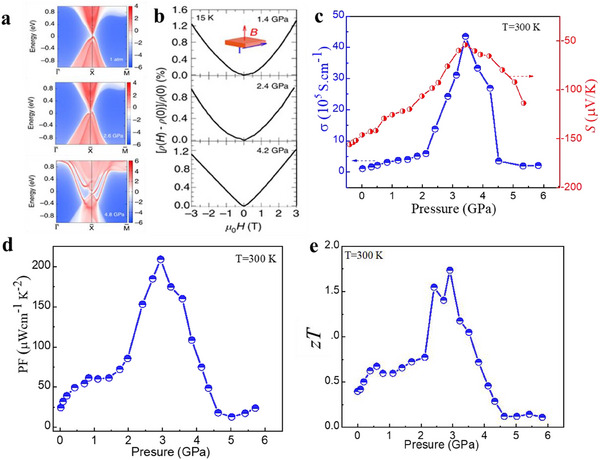
Enhancement of thermoelectric performance by pressure‐indueced topological phase transition. a) Energy and momentum dispersion with the local density of states on the (001) surface at ambient pressure, 2.6 GPa, and 4.8 GPa for Cr‐doped PbSe. The red regions indicate the bulk energy bands and the blue regions indicate the bulk energy gaps. A non‐trivial topological phase transition occurs above 2.6 GPa. b) The magneto‐resistivity [*ρ*(*H*) − *ρ*(0)]/*ρ*(0) at 15 K for selected pressures as a function of the magnetic field strength *H*.^[^ ^87^ ^]^ The linear magneto‐resistivity indicates the experimental realization of the topological states in this material. c) Pressure dependence of electrical conductivity and Seebeck coefficient. d) Pressure dependence of power factor (*PF*). e) Pressure‐dependence of *zT* at 300K. Figure adapted (reproduced) with permission,^[^ ^87^ ^]^ Copyright 2019, Spring Nature.

The modulation of electronic band structures, such as by enhancing the SOC or inducing an odd number of band inversions, can drive transitions between trivial insulators and topological states. Recent studies have shown that inducing topological phase transitions can significantly increase the TE power factor of topological materials.^[^ ^85^, ^86^ ^]^ A notable breakthrough in the TE performance of Cr‐doped PbSe was achieved by inducing a topological phase transition through lattice compression using a diamond anvil cell.^[^ ^87^ ^]^ The calculated band structure for Cr‐doped PbSe (**Figure** **5**a) reveals a pressure‐driven band inversion, where the bandgap near the high‐symmetry point L in the Brillouin zone narrows down, closes at 2.6 GPa, and then reopens with an inversion between the conduction band and the valence band, indicating the occurrence of the topological phase transition. At ambient pressure, there are no surface states, but at 2.6 GPa, a nontrivial topological crystalline insulator phase is formed, with the appearance of metallic surface states around the X point, while the bulk remains insulating. The linear magnetoresistance (Figure 5b) at low magnetic fields, a hallmark of TIs, is observed when the material passes the topological phase transition, providing direct transport evidence for the experimental realization of the topological crystalline surface states in Pb_0.99_Cr_0.01_Se under pressure. The *S* of Cr‐doped PbSe increases significantly with pressure, reaching a maximum value at 3.4 GPa before dropping (Figure 5c). Compared to the σ at ambient pressure, the σ at the topological phase transition pressure point is improved by 40 times. Such high σ is more than sufficient to compensate for the drop in thermopower and the rise in thermal conductivity, leading to a large power factor (Figure 5d) and a record high *zT* of 1.7 at room temperature (Figure 5e).

**Figure 6:**
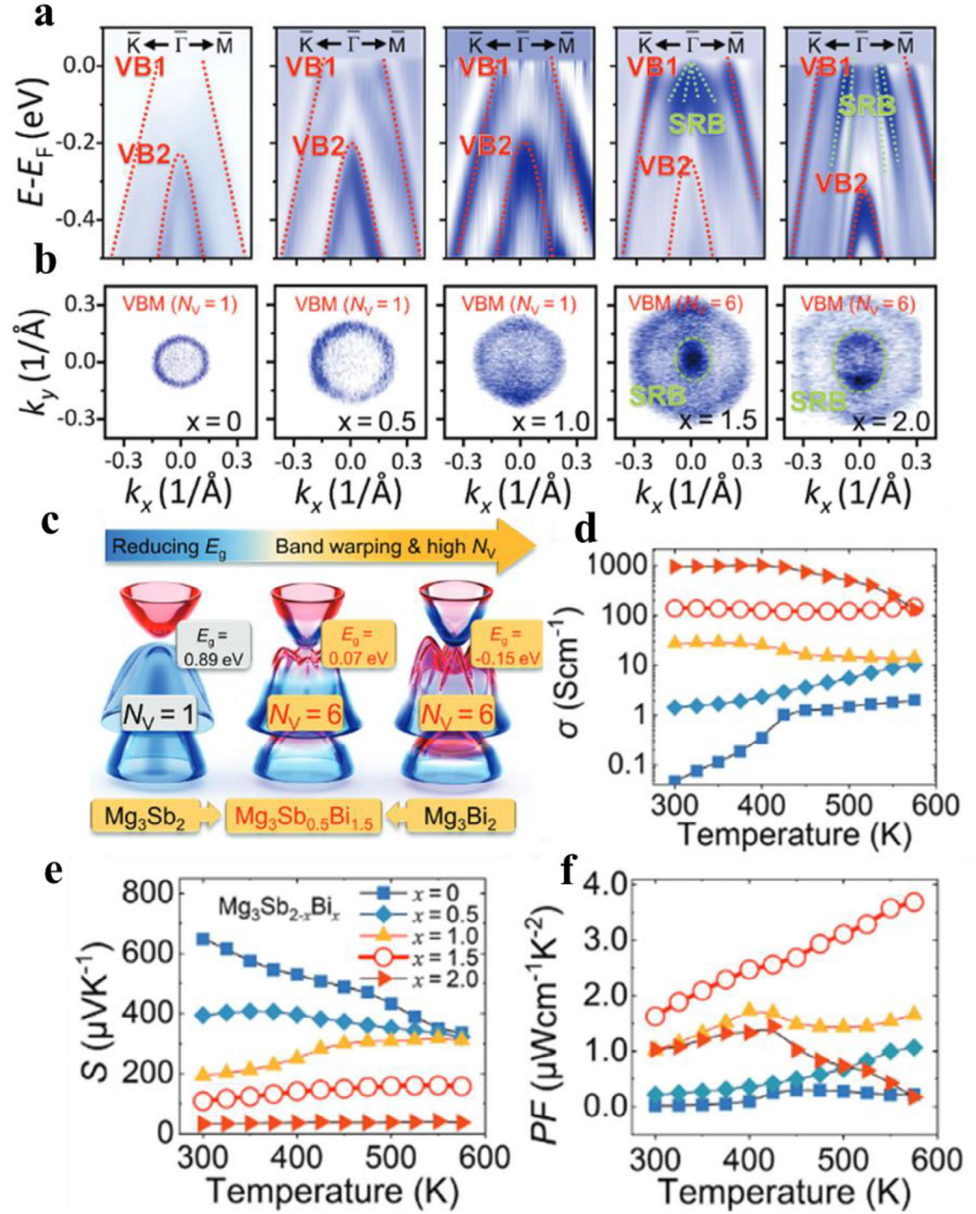
Enhancement of thermoelectric performance by alloying‐induced topological phase transition. a) Second derivative spectra of energy distribution curves (EDC) from ARPES measurements along the $\bar{K}-\bar{\Gamma }-\bar{M}$ direction of Mg_3_Sb_2−_ *_x_* Bi*_x_*. b) Corresponding ARPES Fermi surface maps. The bulk valence bands (VB1 and VB2) and the surface resonance band (SRB) are marked by red and green lines, respectively. The SRB and the transition of Fermi surface map emerges with *x* >1.5, indicating the occurrence of topological electronic transition. c) Schematic diagram of the topological electronic transition of Mg_3_Sb_2−_ *_x_* Bi*_x_* modeled using the *k*.*p* theory. The mixing color of blue and red at the band edges indicates the *s*‐*p* hybridization due to spin‐orbit coupling (SOC) effect and band inversion. e,f) Temperature dependence of electrical conductivity d), Seebeck e) and power factor f) Figure adapted with permission from,^[^ ^88^ ^]^ Copyright 2024, Wiley.

In addition to external pressure, chemical doping, and alloying can also induce topological phase transitions. For example, in the rock‐salt system, SnTe, a TCI, alloying with a normal insulator like PbTe induces a topological phase transition. At a critical composition x_c_, the bandgap closes, giving rise to linear Dirac cones. This band structure evolution significantly impacts TE properties. A similar effect is observed in Mg_3_Sb_2_, a trivial semiconductor, with Bi triggers a transition to a topological semimetal in Bi‐rich Mg_3_Sb_2‐x_Bi_x_. ARPES and Fermi surface mapping of Mg_3_Sb_2‐x_Bi_x_ (**Figure** **6**a,b) reveal that for compositions with x = 0, 0.5, and 1.0, the material retains its trivial semiconductor nature, characterized by a valence band maximum at the Γ point and a low valence band degeneracy (*N*_v_ = 1). However, when x reaches 1.5, the material exhibits a surface resonance band (SRB) and a hexagonal‐ring‐shaped Fermi surface, indicating the onset of a topological electronic transition, accompanied by a reduced bandgap and increased valence band degeneracy (Figure 6c). As a result, the σ (Figure 6d) increases, and the *S* (Figure 6e) decreases significantly with increasing Bi content, dropping from 110 µV K^−1^ at 300K for Mg_3_Sb_0.5_Bi_1.5_ to 32 µV K^−1^ for Mg_3_Bi_2_ at the same temperature. Despite this reduction in *S*, the power factor (Figure 6f) reaches an optimal value of 1.63 µW cm^−1^ K^−2^ at 300K and 3.69 µW cm^−1^ K^−^^2^ at 575 K for Mg_3_Sb_0.5_Bi_1.5_ sample.^[^ ^88^ ^]^ This improvement is attributed to the combination of high hole density, enhanced carrier mobility, and a moderate *S* enabled by the positive bandgap and a high valence band degeneracy. Thus, topological electronic transition presents a promising strategy for enhancing the TE performance of materials.

### 2.7 Impact of Topological Surface States on Thermoelectric Properties

**Figure 7:**
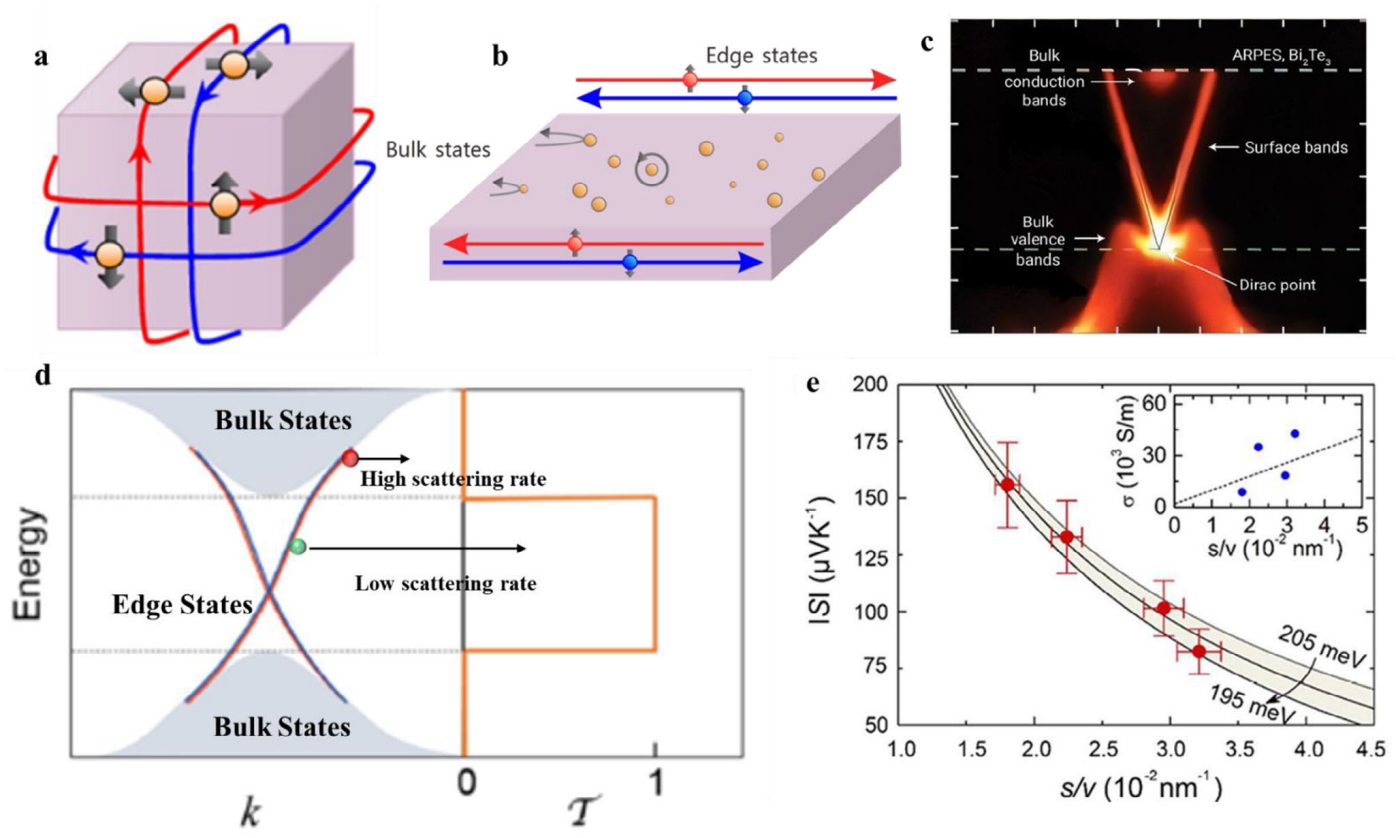
Topological surface states. a) Schematic of 3D topological surface states. a) Adapted with permission from^[^ ^89^ ^]^ under a Creative Commons Attribution 4.0. b) 2D topological surface/edge states. The bulk states are scattered by disorders and defects, whereas the edge states remain minimally affected. b) Adapted with permission from^[^ ^91^ ^]^ under a Creative Commons Attribution 4.0 International License. c) Electronic structure of Bi_2_Te_3_ from angle‐resolved photoemission spectroscopy (ARPES). The bulk valence bands and bulk conduction bands are separated by a bandgap, yet the gapless surface bands cross the bulk bandgap. c) Adapted with permission.^[^ ^90^ ^]^ Copyright 2009, The American Association for the Advancement of Science. d) Schematic band structure of 2D topological materials, illustrating the topological edge states within the bulk bandgap. Surface carriers in the bulk bandgap, which are protected against backscattering by time‐reversal symmetry, have a lower scattering rate than surface carriers in the bulk bands, which are subjected to bulk‐surface interactions. This results in a boxcar‐shaped transmission probability due to energy‐dependent elastic scattering. d) Adapted with permission from^[^ ^91^ ^]^ under a Creative Commons Attribution 4.0 International License. e) Seebeck coefficient and electrical conductivity (inset) of Bi_2_Se_3_ nanowires with varying surface‐to‐volume ratios. e) Adapted with permission.^[^ ^92^ ^]^ Copyright 2009, Royal Society of Chemistry.

In TIs, the bulk states with a bandgap are confined to the interior of the material, whereas the surface states with Dirac‐like linear dispersions are predominantly localized at the surface in 3D TIs or along edge for 2D TIs^[^ ^89^ ^]^ (**Figure** **7**a,b). These surface states exhibit exotic quantum phenomena that are absent in trivial materials, including robust protection against backscattering, spin‐momentum locking, and massless Dirac fermion behavior. Angle‐resolved photoemission spectroscopy (ARPES) serves as a powerful tool for directly probing the energy dispersion and momentum characteristics of these states (Figure 7c).^[^ ^90^ ^]^ The efforts have been made to have a better understanding of exotic phenomena of TIs in recent years. The interest in TIs as potential TE materials is largely driven by the prospect of leveraging their topological surface states to enhance TE performance. In this context, we provide an overview of the current understanding of how TI surface states contribute to TE transport and discuss key factors that govern their role in charge transport phenomena.

#### 2.7.1 Dual‐Channel Transport Mechanism

The coexistence of bulk and surface states (Figure 7d) necessitates a careful evaluation of their respective contributions to charge transport. A parallel circuit model provides an effective framework for describing the TE properties of TIs by incorporating both bulk and surface transport channels. In this model, the total TE transport quantities are expressed as follows:^[^ ^30^ ^]^

(2) $$G_{t}=G_{s}+G_{b}$$

(3) $$S_{t}=\frac{S_{s}G_{s}+S_{b}G_{b}}{G_{s}+G_{b}}$$

(4) $$zT=\frac{G_{t}S_{t}^{2}T}{K}$$

where *G = σA/L* is the electrical conductance and *K = κA/L* is the thermal conductance. Subscripts *s* and *b* denote surface and bulk state contributions, respectively, with 𝐴 representing the cross‐sectional area and 𝐿 the material length. Since the *S* contributions from surface and bulk states vary with geometric size, it is more appropriate to describe TE effects using conductance (*G*) rather than electrical conductivity (σ). This indicates that the TE properties of TIs are not intrinsic but strongly geometry‐dependent.

#### 2.7.2 Size Effect

The topological surface states and bulk states show distinct transport properties and physic dimensions. The relative contributions of topological surface states and bulk states to TE transport properties are largely determined by the number of available states, which are influenced by the geometry of the sample. In bulk TIs, surface states contribute minimally to transport due to their limited density of states. For instance, in Bi₂Se₃ TIs, the *S* decreases while σ increases with the increasing surface‐to‐volume ratio (Figure 7e). Experimental studies on Bi_2_Se_3_^[^ ^92^ ^]^ and Bi_1‐x_Sb_x_.^[^ ^93^ ^]^ nanowires have shown that *S* and σ exhibit a non‐monotonic dependence on wire diameter, which were attributed to the presence of TI surface states. Generally, for Bi₂Te₃ TIs, the impact of surface states on TE performance becomes more pronounced when the sample size is on the order of a few nanometers, except for Bi_1.1_Sb_0.9_Te_2_S.

In addition, when the thickness of TI thin film decreases to the order of the localization length of boundary states (∼1–10 nm), a subbandgap opens at the Dirac point due to hybridization between the top and bottom surface states.^[^ ^94^, ^95^ ^]^ The surface gap may supress σ by breaking time‐reversal symmetry, but it also enhances *S* due to the emergence of massive Dirac fermions and the suppression of bipolar effects. Theoretically, hybridization‐induced bandgaps in Bi₂Te₃ thin films have been predicted to enhance *zT* of surface states over a broad range of chemical potentials at low temperatures.^[^ ^96^, ^97^ ^]^ Experimentally, a Bi_1.5_Sb_0.5_Te_1.7_Se_1.3_ thin film with 4 quintuple layers (QLs), where a surface bandgap is present, exhibits a higher *S* than an 8 QL film, where the gap is closed. This suggests that suppressing bipolar conduction via a surface gap can improve TE performance.^[^ ^98^ ^]^ Beyond hybridization, external perturbations such as strain and magnetic fields have also been predicted to induce a surface bandgap and enhance the power factor.^[^ ^99^ ^]^

#### 2.7.3 Anomalous Seebeck Effect

In TI nanostructures, the surface Seebeck coefficient (*S*_s_) can exhibit an opposite sign to that of bulk carriers, a phenomenon known as the anomalous Seebeck effect. This effect arises due to the unique scattering mechanisms of surface carriers. Generally, the electronic states below and above the Fermi level contribute opposite signs of the *S*: if transport is dominated by charge carriers below the Fermi level, *S* is positive, whereas if carriers above the Fermi level dominate, *S* is negative. The *S* can be estimated by using the Sommerfield expansion:^[^ ^100^ ^]^

(5) $$S={\left.-\frac{\pi^{2}k_{B}^{2}T}{3e}\frac{\partial \ln\left[\tilde{T}\left(E\right)\right]}{\partial E}\right|}_{E=E_{F}}$$

Where $\tilde{T}\left(E\right)=M\left(E\right)T\left(E\right)$, *M*(*E*) represents the distribution of conduction modes at a given energy *E*, determined by the band structure (e.g., band effective mass, density of states, band degeneracy), and T(E) is the transmission probability, which is related to the mean free path λ(*E*) or the scattering time τ(*E*). Equation (5) suggests that a large *S* requires a steep slope of T(E). This enhancement in *S* can occur through two mechanisms: 1) increasing energy dependence of *M*(*E*), such as by introducing a hump in the density of states (e.g., resonant levels), and 2) increasing the energy dependence of τ(*E*), for instance, via resonant scattering or low‐energy electron filtering effects.

The energy dependence of τ(*E*) plays a crucial role in TE transport properties of TIs. For example, in a 2D TI system (Figure 7d), electron edge states within the bulk bandgap are immune to backscattering from nonmagnetic defects,^[^ ^101^ ^]^ and therefore have long scattering time. Whereas electron edge states near the bulk band edge would suffer from the edge‐bulk interactions, leading to a short scattering time. This strong energy dependent scattering can generate a sizeable *S* even though the topological surface states are gapless, and the anomalous Seebeck. Assuming the *E*_F_ is is situated near the bulk conduction band edge and above the Dirac point, charge carriers are expected to exhibit electron‐like behavior, as indicated by Hall measurements, with a negative *S*_b_. Due to edge‐bulk interactions, the edge states above *E*_F_ are scattered, and edge states below *E*_F_ ​ dominate the transport, resulting in a positive *S*_s_. In this scenario, the sign of the total *S* will be determined by the ratio *G*_b_/*G*_s_. When the surface states dominate the electron transport (*S*_b_<0<*S*_s_ and *G*_s_>>*G*_b_), the total *S* will reverse its sign, even if *E*_F_ is near the conduction band edge. The anomalous *S* value, where the polarity of *S* is opposite to that of the Hall effect, has been theoretically predicted for both 3D and 2D TIs^[^ ^96^ ^]^ and experimentally observed in five quintuple layer (Bi_1−x_Sb_x_)_2_Te_3_ films^[^ ^29^ ^]^ and (Bi_1−x_Sb_x_)_2_Te_3_ nanowires.^[^ ^102^, ^103^ ^]^

### 2.8 Relative Contributions of Bulk and Surface States

The relative contribution of bulk and topological surface states to the TE transport properties of TIs are influenced by various factors, including 1) geometric size, 2) temperature, and 3) Fermi level. These important factors should be considered to utilize the surface and bulk channels synergistically. First, as discussed earlier, geometric factors, such as thickness and grain size, strongly affect the relative contribution of surface states. Second, surface states are inevitably influenced by inelastic electron‐phonon scattering, which becomes more pronounced at higher temperatures. As a result, surface‐state contributions tend to be overwhelmed by bulk states at elevated temperatures. Typically, surface states dominate the transport properties usually occur at temperatures below ≈200 K.^[^ ^104^ ^]^ However, TE converters usually operate at elevated temperatures, where bulk carrier contributions dominate. Third, the maximum *S* values for bulk and surface states are reached at different *E*_F_. The optimal *E*_F_ for maximum *S*_s_ is near the bulk band edge, while for maximum *S*_b_, it is typically within bulk bandgap. Moreover, *S*_s_ and *S*_b_ may have opposite signs, leading to mutual compensation and a reduced overall *zT*.^[^ ^30^ ^]^ Thus, achieving optimal performance requires balancing these contributions.

Numerous theoretical calculations have explored the TE efficiency of both 2D and 3D TIs, utilizing various approximations.^[^ ^30^, ^96^, ^105^, ^106^ ^]^ Some studies predicted *zT* values approximately an order of magnitude higher than those of conventional bulk TE materials. For instance, recent studies predicted a *zT*∼3 for graphene‐based 2D TI nanoribbons^[^ ^107^ ^]^ and a maximum *zT* of≈2.0 in Bi_2_Te_3_ thin film.^[^ ^108^ ^]^ More recently, Liu et al demonstrated that *zT* depends on the electron transmission probability (T(E)), reaching a maximum when T(E) assumes a boxcar shape.^[^ ^91^ ^]^ This boxcar‐shaped T(E) is predicted to emerge in 2D TIs, where the topologically protected, gapless 1D edge states reside within the bulk bandgap (Figure 7d). Consequently, electrons in the edge modes within the bandgap are immune to scattering, resulting in T(E)= 1. However, edge electrons near the bulk area may experience scattering, with T(E) gradually approaching zero by introducing disorders within the bulk region, which extends the transport length. This evolution of the T(E) toward a boxcar shape, combined with disorder‐induced phonon scattering (which reduces thermal conductivity), possibly further enhances *zT*.

Experimentally, high *zT* values have been observed in nanostructured bulk bismuth telluride materials,^[^ ^109^ ^]^ where the improvement in properties is mainly attributed to reduced lattice thermal conductivity. Several experimental effort^[^ ^102^, ^110^, ^111^ ^]^ have investigated the effects of topological surface states on TE properties, but *zT* values exceeding unity have rarely been reported. This is likely due to the competitive contributions of bulk and surface states to the overall transport properties. Additionally, various non‐topological states—such as bulk bands and localized impurity states—also contribute to electron transport in real TI systems, complicating the task of isolating the effects of nontrivial surface states from trivial ones. This complexity poses a significant challenge in determining whether topological states can substantially enhance the TE efficiency of TIs.

### 2.9 Thermoelectric Properties of Intrinsic Topological Surface States

**Figure 8:**
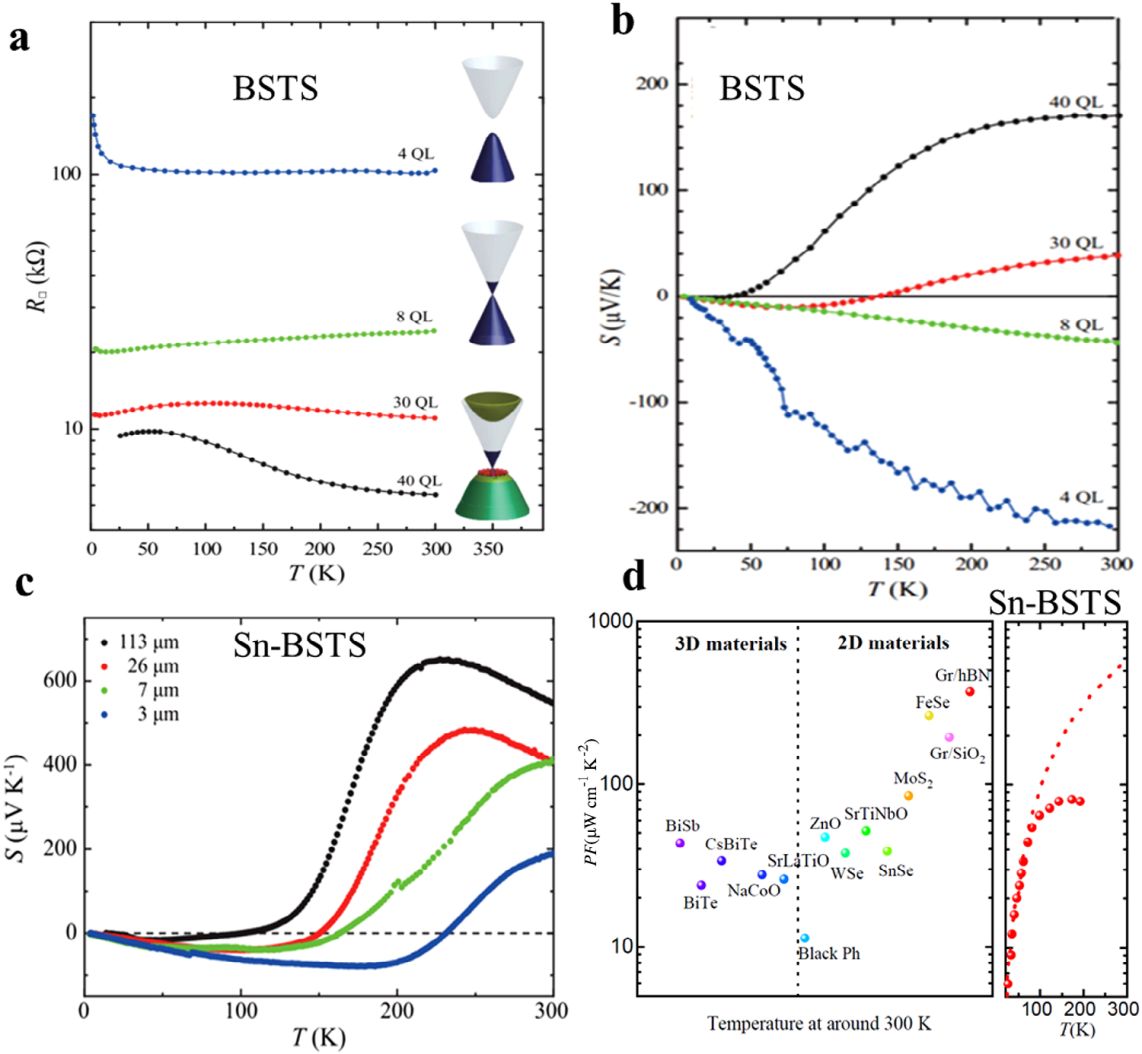
Thermoelectric properties of topological insulator films. a) Electrical transport properties of Bi_2−_ *_x_* Sb*_x_* Te_3−_ *_y_* Se*_y_* (BSTS) TI thin film with 40, 30, 8, and 4 quintuple layers(QLs). *R* denotes the sheet resistance of BSTS thin films. The inset shows schematic band illustrations of each film: the 40 QL and 30 QL BSTS are in a mixed state of a bulk state and a metallic topological surface Dirac state (m‐TSDS), the 8‐QL BSTS is a gapless m‐TSDS, and the 4‐QL BSTS is gap opened (g‐TSDS). b) Seebeck coefficient of BSTS thin films with 40‐, 30‐, 8‐, and 4‐ QLs. a,b) Adapted with permission.^[^ ^98^ ^]^ Copyright 2019, American Physical Society. c) Thermopower as a function of four TI Sn‐Bi_1.1_Sb_0.9_TeS_2_ flakes with different thicknesses of 113, 26, 7, and 3 µm. d) Comparison of the power factors between 2D or 3D thermoelectric materials and Sn‐BSTS flakes. The PF of Sn‐BSTS is plotted on the right side as a function of *T* (red balls). The *PF* shows an increase with an increase in *T* below 80 K, and the saturation above 80 K is caused by the contribution of the bulk carriers thermally excited at high *T*. The red dashed line in the right panel represents the intrinsic *PF* values of pure TSDS of Sn‐BSTS at high *T* according to the theoretical prediction. c,d) Adapted with permission.^[^ ^51^ ^]^ Copyright 2021, American Physical Society.

To detect the intrinsic TE performance of topological surface states, TI nanostructures must possess low bulk concentrations, few defects, highly insulating bulk states, and a Dirac point located within the bulk bandgap. Recent studies have identified Bi_2−_ *_x_* Sb*_x_* Te_3−_ *_y_* Se*_y_* (BSTS) and Sn‐Bi_1.1_Sb_0.9_TeS_2_(Sn‐BSTS) as highly bulk insulating 3D TIs with ideal 2D Dirac surfaces, making them excellent platforms for studying the TE properties of pure topological surface states. Tanigaki et al.^[^ ^51^, ^98^ ^]^ reported the intrinsic TE performance of topological surface states in 40‐quintuple‐layer (QL) BSTS thin films and Sn‐BSTS single‐crystal flakes. The 40‐QL BSTS sample (**Figure** **8**a,b) exhibited semiconducting behavior with a positive *S* that demonstrated a nonlinear *T* dependence. When the thickness was reduced to 8 QL, a transition to intrinsic metallic behavior was observed, as indicated by a temperature‐dependent resistance and a negative, linear temperature‐dependent *S*, reflecting the metallic nature of the topological surface Dirac states (m‐TSDS). Further reducing the thickness to 4 QL led to an increase in resistance at low temperatures, and a nonlinear increase in *S* to −212 µV K⁻¹, suggesting the opening of a gap in the topological surface states (g‐TSDS). Intriguingly, the *S* of pure m‐TSDS in BSTS 3D TIs was an order of magnitude larger than those of conventional metals, yielding a *zT* value of 0.1. Compared with BSTS, Sn‐BSTS has superior bulk insulation, allowing its pure m‐TSDS to persist at critical thicknesses on the micron scale. At 77K, significant enhancements were observed in both *S* (≈58 µV K^−1^) and *PF* (≈50 µW cm^−1^ K^−2^) for 3‐µm flakes of the pure TSDS (Figure 8c,d). Above 80K, thermally excited bulk carriers began to dominate, leading to a reduction in total *S* and *PF* due to the opposite signs of *S*_b_ and *S*_s_. The *PF* of pure TSDS monotonically increased with *T* and was expected to reach *PF* = 600 µW cm^−1^K^−2^ at 300K, based on theoretical simulations (Figure 8d). The good TE performance of m‐TSDS in Sn‐BSTS TIs can be attributed to its strong energy‐dependent relaxation time *τ*(E), which arises from the bulk‐ edge state interaction.

### 2.10 Topological Semimetals as Thermoelectric Materials

**Figure 9:**
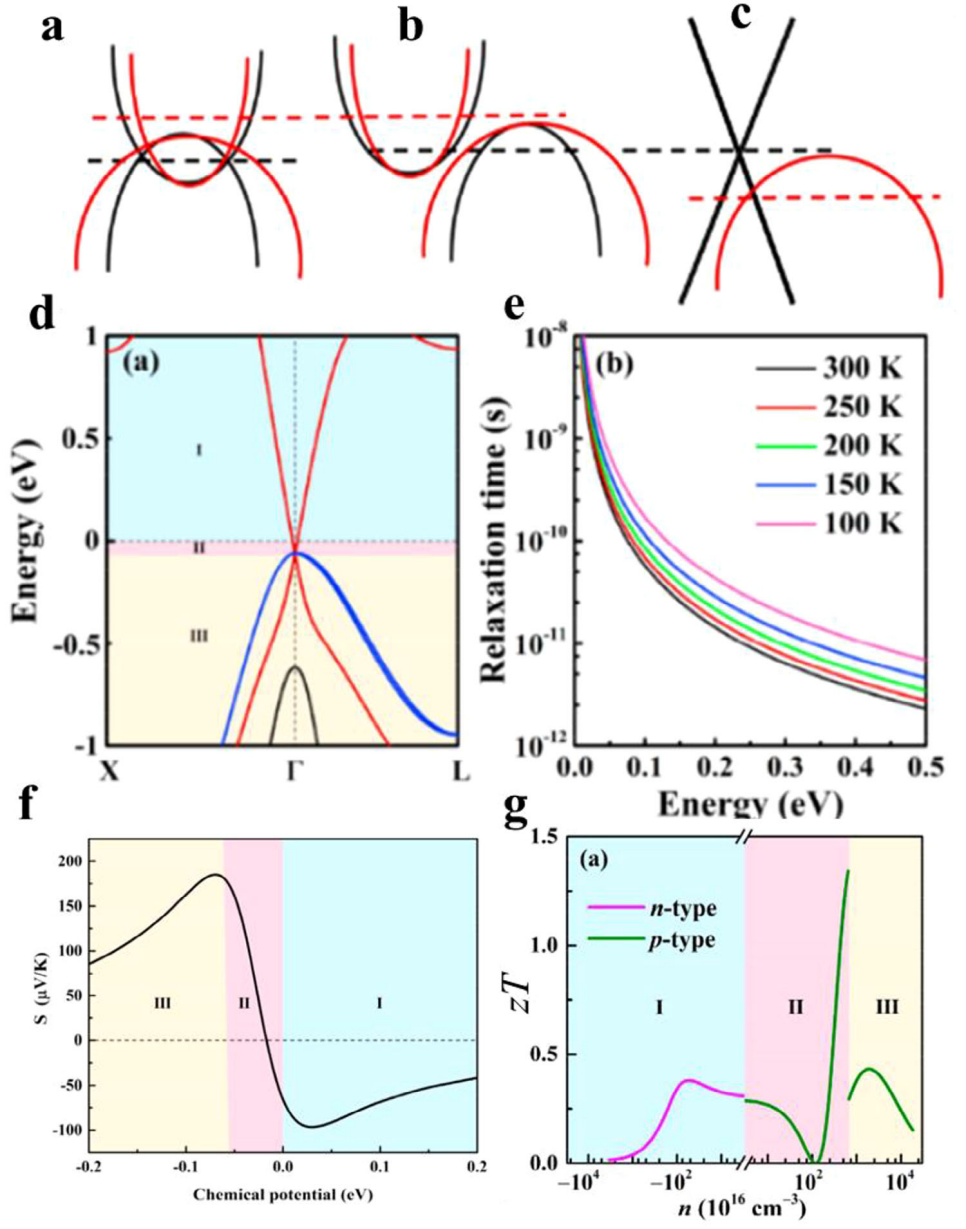
Topolocial semimetals for thermoelectric materials. a) Schematic illustration of a semimetal with parabolic bands. b) Schematic of an indirect semimetal with parabolic bands. c) Schematic of a Dirac or Weyl semimetal with linear dispersion. The Fermi level is indicated by the dashed line. In each case, the band structure can be symmetric (black curves) or asymmetric (red curves). a–c) Adapted with permission.^[^ ^112^ ^]^ Copyright 2019, American Physical Society d) Energy band structure of Na₂AgSb, where the red and blue lines represent the Dirac band and a valence band passing through the Dirac point, respectively. e) Energy‐dependent relaxation time of the Dirac band at different temperatures. f) Calculated Seebeck coefficients of Na₂AgSb as a function of chemical potential at room temperature. g) Calculated *zT* values of Na₂AgSb as a function of carrier concentration at room temperature. d–g) Adapted with permission.^[^ ^113^ ^]^ Copyright 2021, Elsevier.

Conventional high‐performance TE materials are typically semiconductors with small bandgaps, as they strike a trade‐off between the σ and *S*. Semiconductors generally exhibit *S* values that are orders of magnitude larger than those of metals due to the bandgap, which breaks the symmetry between electrons and holes. In contrast, semimetals, characterized by a small overlap between conduction and valence bands and thus a near‐zero band gap (**Figure** **9**a–c), often suffer from significant bipolar effects due to the coexistence of electron and hole carriers^[^ ^112^ ^]^. As a result, semimetals are generally thought to have lower *S* due to the cancellation between electron and hole contributions, and high bipolar thermal conductivity resulting from electron‐hole annihilation, which leads to poor TE performance.

Based on the two‐carrier model, the *S* in semimetals can be expressed as *S* = (σ_h_ *S*_h_ + σ_e_ *S*_e_)/(σ_e_ + σ_h_), where the subscripts *h* and *e* denote the contribution of electrons and holes to transport properties, respectively. Since *S*_e_ and *S*_h_ have opposite signs, achieving a large overall *S* requires a significant ratio of *S*_h_ /*S*_e_ and σ_h_/σ_e_. Both *S* and σ depend on the density of states (DOS) effective mass and carrier mobility, so the asymmetry between conduction and valence bands can be quantified using the weighted mobility ratio, $A=\left(N_{e}m_{e}^{*3/2}\mu_{e}\right)/\left(N_{h}m_{h}^{*3/2}\mu_{h}\right)$,^[^ ^114^, ^115^, ^116^ ^]^ where the *N*_e_ (*N*_h_) is the number of conduction (valence) band degeneracy, *m*_e_ (*m*_h_) and 𝜇_e_(𝜇_h_) is the electron (hole) effective mass and carrier mobility, respectively. When the A>>1 or A<<1, a large *S* and weak bipolar effects can be expected. This requires asymmetric band structures around *E*_F_ or substantial differences in the energy‐dependent scattering rates of electrons and holes. Figure 9a–c illustrates three different groups of semimetals based on their band structure characteristics, with the asymmetric band structures (depicted by red curves) offering favorable conditions for enhanced thermopower due to the significant density of states differences between hot and cold carriers.

In TSMs, linear‐dispersion bands near the Fermi level, such as the Dirac bands found in materials like Bi, Sb, and Na_3_Bi, provide high carrier mobility but generally contribute little to the *S* due to their intrinsic symmetry. However, if additional conventional parabolic bands are present near the Dirac point, as depicted by the red curves in Figure 9c, the resulting density of states can break the symmetry around the Fermi level.^[^ ^117^ ^]^ For example, first‐principles calculations predict the Na_2_AgSb, a Dirac semimetal, exhibits a large *S* of ≈200 µV K^−1^ at 300K.^[^ ^113^, ^117^ ^]^ The band structure (Figure 9d) of Na_2_AgSb features a Dirac band alongside an extra parabolic valence band passing through a linear band at the Dirac point, which leads to a strong band asymmetry around the Fermi level. This asymmetry, where the DOS of holes exceeds that of electrons, weakens electron‐hole compensation, contributing to improved TE properties. Based on the location of the Fermi level and Dirac point, the band structure is divided into three distinct regions (Regions I, II, and III in Figure 9d). Notably, the relaxation time (Figure 9e) of the Dirac band is several orders of magnitude longer than that of the parabolic bands between 100 and 300K, enabling high electrical conductivity in Region II. The relationship between *S* and chemical potential at room temperature is shown in Figure 9f, where the maximum *S* occurs when the Fermi level lies at the boundary between Regions I and II. Na₂AgSb also exhibits intrinsically low lattice thermal conductivity (0.36 W m^−1^ K^−1^ at 300K). Ignoring lattice thermal conductivity, the *zT* can be approximated as $zT\approx \frac{S^{2}\sigma }{\kappa_{e}}T=\frac{S^{2}}{L}$, where *L* is the Lorenz number, determined by the Wiedemann‐Franz law ($L\approx \frac{\kappa_{e}}{\sigma T}$). Thus, *zT* is primarily governed by the magnitude of *S*. Given that Na_2_AgSb have a large *S*, this leads to a high *zT* value of 1.3 at 300 K in Region II, as shown in Figure 9g.

**Figure 10:**
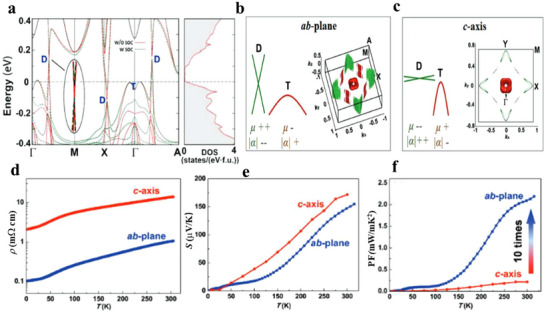
The band structure and thermoelectric properties of topological semimetals YbMn_2_Sb_2._ a) Calculated band structures and density of states (DOS), including band structures with spin‐orbit coupling (SOC, “w soc”) and without SOC (“w/o density of states (DOS)soc”). “D” presents the Dirac bands and “T” refers to traditional regular bands. A very small gap (<10 meV) in the Dirac bands can be introduced by SOC, The inset shows an enlargement of the Dirac band indicating the extremely small bandgap due to SOC. b,c) Fermi surface illustration in the *ab*‐plane (b) and *c*‐axis direction (c); d–f) Temperature dependence of resistivity (d), Seebeck coefficient (e), power factor (f). Figure adapted with permission from^[^ ^118^ ^]^ under a Creative Commons CC BY license.

The coexistence of a regular hole pocket and 2D Dirac Fermi surfaces near the Fermi level is also found in topological semimetal YbMnSb_2_(**Figure** **10**).^[^ ^118^ ^]^ Interestingly, the 2D Dirac bands and parabolic bands show obvious anisotropy, with different curvatures along the ab‐plane and c‐axis, as illustrated in Figure 10b,c. While highly dispersive bands provide high carrier mobility, they result in lower *S*, whereas flatter bands lead to larger *S* but lower carrier mobility. This anisotropy leads to distinct TE transport properties (Figure 10d–f) along different crystallographic directions. To achieve a trade‐off between 𝜎 and *S* (this can be qualified by weighted mobility), one innovative strategy is to combine highly dispersive Dirac bands and heavy regular bands. The higher power factor along the ab‐plane compared to the c‐axis, as shown in Figure 10f, suggests that the coexistence of these Dirac and parabolic bands is advantageous for optimizing TE performance.

**Figure 11:**
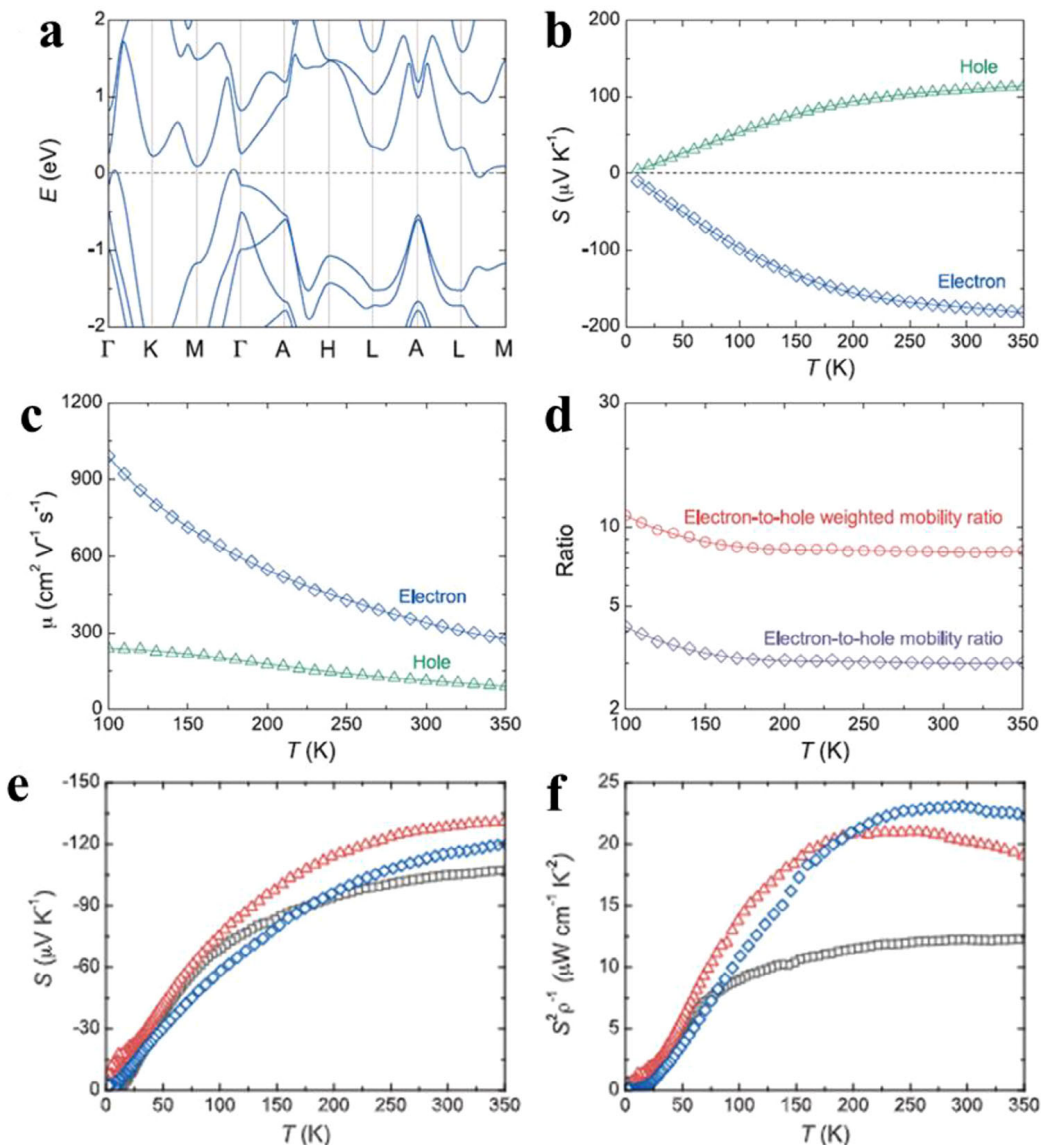
Thermoelectric properties of Mg_3.2_Bi_2‐x_Te_x_. a) Calculated band structure of Mg_3_Bi_2_ with the band overlap energy shifted to −0.1 eV. b) Comparison of partial Seebeck coefficients between electrons and holes. c) Comparison of mobilities between electrons and holes. d) Electron‐to‐hole mobility ratio and electron‐to‐hole weighted mobility ratio. e) Temperature dependence of Seebeck coefficient. f) Temperature dependence of power factor. Figure adapted with permission.^[^ ^114^ ^]^ Copyright 2019, The American Association for the Advancement of Science.

There are several topological semimetals that has been reported to show good TE performance. Koshi et al reported that the 1D Dirac semimetal Ta_4_SiTe_4_ shows a very large thermopower of over 400 µV/K^−1^ and a very large power factor of 80 µW cm^−1^K^−2^ at 130K.^[^ ^119^, ^120^ ^]^ Similarly, Mg_3_Bi_2_, identified as a topological nodal‐line semimetal, is emerging as a promising material for TE cooling and power generation.^[^ ^114^ ^]^ The calculated band structure of Mg₃Bi₂ (**Figure** **11**a) reveals a band overlap energy of ≈0.1 eV, based on two‐band modeling. The conduction and valence bands show valley degeneracies of 6 and 1, respectively. In the two‐band model, the partial *S* (Figure 11b) for electrons was noticeably larger than that for holes, and the electrons have higher carrier mobility (Figure 11c) compared to holes. The significant difference in effective masses between the conduction and valence bands, along with a high electron‐to‐hole weighted mobility ratio (Figure 11d), contributes to the high thermopower (Figure 11e) and power factor (Figure 11f) observed. Additionally, partial substitution of Bi with Sb in Mg_3_Bi_2_, induces a band structure transition from semimetallic to semiconducting, further enhancing *S*. Combining with reduced lattice thermal conductivity due to alloying effect, a *zT* of≈0.85 at 350K is achieved. These findings indicate that inducing a topological phase transition from semimetals to semiconductors is an effective strategy for improving TE performance. Moreover, cation vacancy ordering in bulk Zintl Eu₂ZnSb₂ has been shown to lead to a topological electronic transition and low thermal conductivity, achieving a *zT* of 1.0 at 823K.^[^ ^121^, ^122^ ^]^

### 2.11 Magneto‐Thermoelectric Effects in Topological Materials

#### 2.11.1 Longitudinal Magneto‐Thermoelectric Effects in Topological Materials

In the absence of magnetic field, the contribution of holes and electrons to thermopower in topological semimetals can cancel each other out, unless there is a significant difference in the weighted mobility between holes and electrons. However, many topological materials, characterized by their linear dispersion bands, exhibit high carrier mobility, which can result in a strong TE response to magnetic fields in both longitudinal and transverse directions. Recently, several topological semimetals have been found to display unusual longitudinal magneto‐Seebeck (denoted as *S*_xx_) and transverse Nernst effects (denoted as *S_x_*_y_) when a magnetic field is applied perpendicular to a temperature gradient. In this section, we first discuss the large magneto‐thermoelectric effects in topological materials.

**Figure 12:**
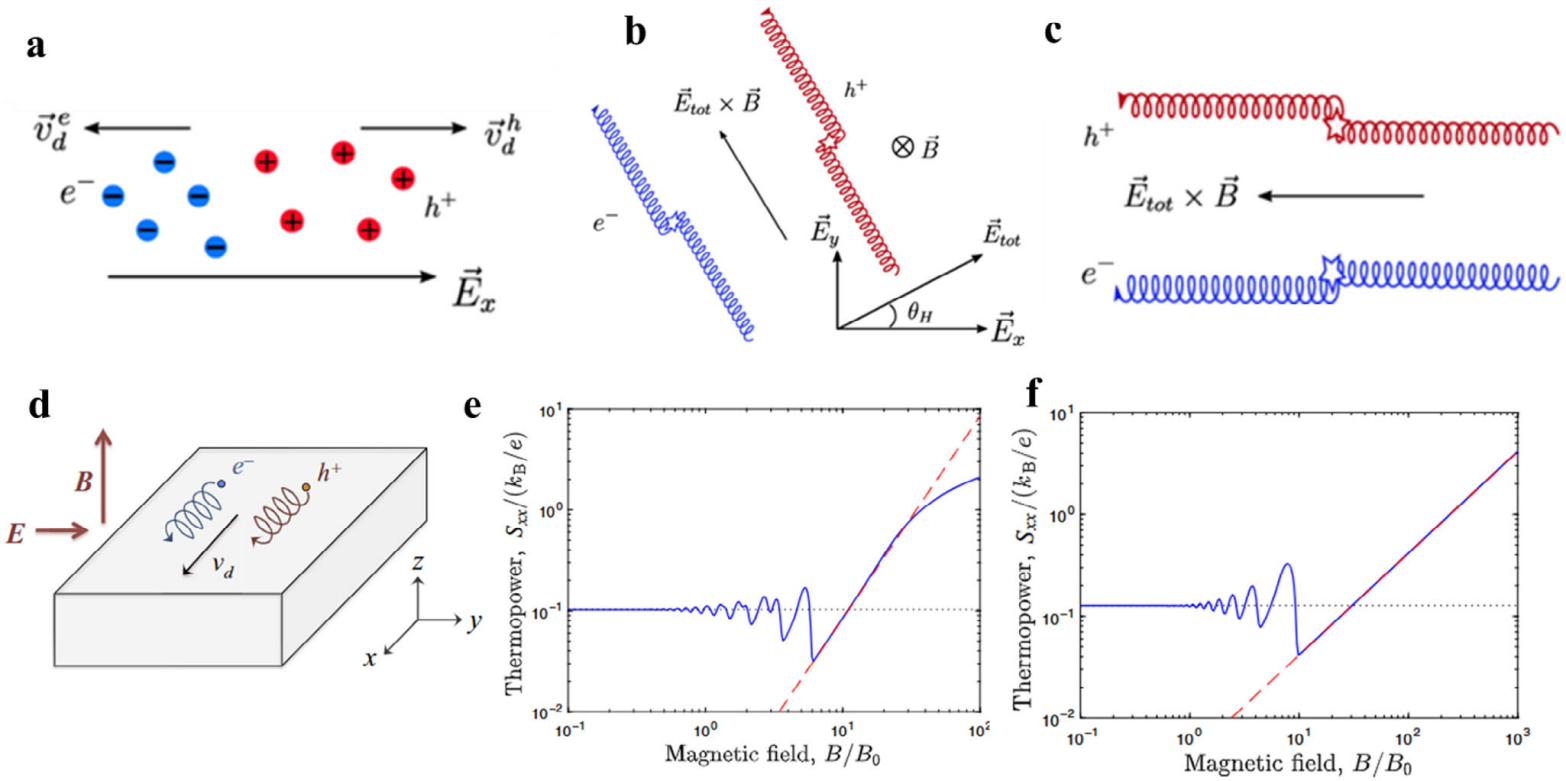
The large magneto‐Seebeck effect in topologic semimetals. a–c) Schematic illustration of the semiclassical motion of electrons and holes in different regimes of magnetic field. (a) At zero or very low magnetic fields, electrons (blue) and holes (red) experience opposite drift velocities under the applied electric field, resulting in near‐cancellation of the heat current and a small thermopower. (b) As the magnetic field increased, the Hall angle θ_H_ remains small, but the heat current is dominated by the x component of the E × B drift, which allows electron and hole carriers to contribute additively. (c) At high magnetic fields, the Hall angle (θ_h_) approaches 90°, and the E × B drift velocity aligns nearly with the current direction (x). a–c) Adapted with permission.^[^ ^124^ ^]^ Copyright 2021, American Physical Society, d) Schematic depiction of the E × B drift of carriers in a strong magnetic field. Electrons and holes drift in the same direction under the influence of crossed electric and magnetic fields. Both types of carriers contribute additively to the heat current in the x‐direction and subtractively to the electric current, resulting in a large Peltier heat (Π_x__x_) and, consequently, a large thermopower (*S*_x__x_). e) Predicted magneto‐thermopower as a function of magnetic field for a degenerate semiconductor with a parabolic dispersion relation. f) Predicted magneto‐thermopower as a function of magnetic field for a gapless Dirac/Weyl semimetal. Unlike in the semiconductor case, the thermopower continues to increase with magnetic field strength without saturation. d–f) Adapted with permission.^[^ ^123^ ^]^ Copyright 2018, The American Association for the Advancement of Science.

The mechanisms underlying magnetic field‐modulated thermoelectric effects in topological semimetals can be explained from multiple perspectives. On one hand, the longitudinal thermopower *S*_xx_ can be understood through the Onsager symmetry relation, expressed as the ratio of the Peltier heat current $J_{x}^{Q}$ to the electrical current $J_{x}^{e}$ defined as $S_{xx}=\frac{1}{T}\cdot \frac{J_{x}^{Q}}{J_{x}^{e}}$. In the absence of magnetic field (*B*), electrons and holes, both carrying heat, move in opposite directions under an applied electric field, leading to the cancellation of contributions in $J_{x}^{Q}$, resulting in a small *S*_xx_ (**Figure** **12**a). When a magnetic field is present and B<<B_H_ (where *B*_H_ is the field at which σ_xy_ and σ_xx_ are equal), the Lorentz force deflects the transport of electrons and holes, creating a y‐component of the electric field, $E_{y}=-\frac{\sigma_{xy}\cdot E_{x}}{\sigma_{xx}}$, where σ_xy_ and σ_xx_ is the electrical conductivity along x direction and Hall conductivity along y direction, respectively, and *E*_x_ is the applied electric field along the x direction (Figure 12b). This y component can generate a $\vec{E}\times \vec{B}$ draft velocity in the x direction, causing the electrons and holes to move in opposite directions and contribute additively to thermopower, in contrast to the subtractive contributions in the zero‐field scenario. As *B* increases, such that B>>B_H_, the Hall angle (𝜃_H_) approaches ≈90 degrees, aligning the $\vec{E}\times \vec{B}$ draft velocity with the current direction x(Figure 12c), leading to a large, field‐enhanced thermopower.^[^ ^123^ ^]^ Fu et al. predicated that under a strong quantizing magnetic field (ω_*c*_τ >> 1, where *ω*_c_ is cyclotron frequency and *τ* is the momentum scattering time), electrons and holes drift in the same direction under a crossed electric and magnetic fields (Figure 12d). This results in both types of carriers contributing additively to the heat current in the x‐direction while contributing oppositely to the electric current, leading to a large Peltier heat (*Π*_x__x_) and, consequently, a large thermopower (*S*_x__x_). The thermopower of trivial semiconductor (Figure 12e) shows a *B* ^2^ enhancement and finally saturates at a value ∼*k*_B_/*e*. In contrast, the thermopower of the Dirac semimetals (Figure 12f) increase lineally with *B* without saturation when reaching the extreme quantum limit, potentially reaching extremely high values under magnetic field.^[^ ^124^ ^]^

On the other hand, the Mott formula suggests that thermopower is also associated with the energy‐dependent scattering time, which can be influenced by momentum‐relaxation processes involving entropy carriers. The magnetic field differentially affects charge carriers based on their energy, inducing an “energy filtering” effect that enhances carrier entropy. The *S*_xx_ can be enhanced until the electrons are forced into a cyclotron motion when *µB* > 1, where *µ* and *B* are the charge carrier mobility and magnetic field, respectively. Moreover, the cyclotron frequency of the charge carriers is also energy dependent. For a general ɛ(*p*)∝*p* ^γ^, the Boltzmann equation gives the cyclotron frequency $\omega \left(\varepsilon \right)={\left.eB\varepsilon^{\prime }\left(p\right)/p\right|}_{p=p\left(\varepsilon \right)}$. The *S*_xx_ was predicated to increases for *γ* < 2, decreases for *γ* > 2, and remains unchanged at *γ* = 2, where *ɛ*, *p*, *γ*, *ɛ′*, and *e* represent energy, momentum, index number, energy differential, and elementary charge, respectively.^[^ ^125^ ^]^ Besides the diffusion mechanism, the phonon‐drag effect at low temperatures and the magnon‐drag effect in magnetic materials also contribute to the Seebeck coefficient, both of which can be modulated by the magnetic field. For example, phonon‐drag can dominate thermopower in many materials at low temperatures.^[^ ^126^, ^127^, ^128^ ^]^ Phonon‐drag thermopower at a particular magnetic field and temperature has been widely investigated in 2D electron systems,^[^ ^129^, ^130^, ^131^ ^]^ where results show that magneto‐thermopower exhibits oscillatory behavior as a function of the magnetic field. Thus, phonon‐drag is a possible mechanism for the large magneto‐thermopower, or Nernst effect, observed in non‐magnetic Dirac/Weyl semimetals.^[^ ^132^ ^]^ Additionally, an unusually enhanced thermopower has recently been reported in the chiral‐lattice magnet MnGe, which exhibits a topological spin texture.^[^ ^133^ ^]^ The large magneto‐thermopower in MnGe is attributed to the strong energy dependence of the charge‐transport lifetime, driven by unconventional carrier scattering from the dynamics of the emergent magnetic field generated by the topological spin texture. Therefore, the modulation of the energy‐dependent density of states and scattering time under a magnetic field is the key origin of enhanced magneto‐thermopower in topological materials.^[^ ^134^ ^]^

**Figure 13:**
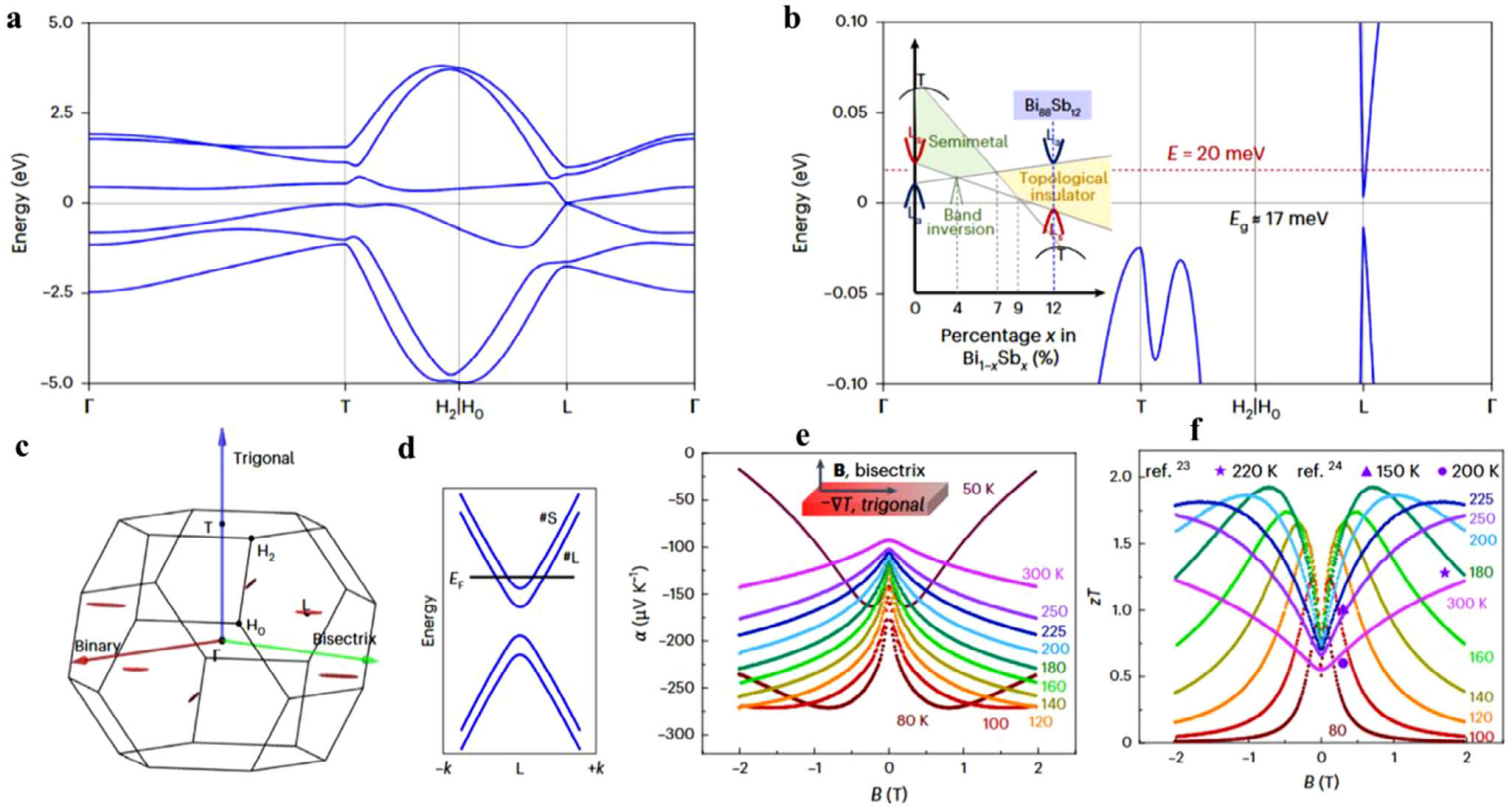
Band structure and magento‐thermoelectric performance of topological insulator Bi_88_Sb_12_. a) Band structure of Bi_88_Sb_12_. b) An enlarged view of the band structure close to the Fermi level at the T and L points. Inset shows the schematic of the composition dependence of the band structures of Bi_1–_ *_x_* Sb*_x_*, with *x* ranging from 0% to 12%. A bandgap *E*_g_ of around 17 meV is found in Bi_88_Sb_12_. c) Brillouin zone and electron Fermi pockets with a Fermi energy at 20 meV. d) Schematic illustration of the band dispersion with Zeeman splitting. A large *g*‐factor in Bi_1–_ *_x_* Sb*_x_* would split the degenerate Fermi surfaces under zero field at L points into two individual pockets, one that is smaller inside (marked #S) and another one that is larger outside (marked #L). The *E*_F_ is reduced for the #S band and increased for the #L band compared to that in the degenerate state. Here *k* is a wave vector. e) Field dependent Seebeck coefficient. f) Field dependent *zT*.Figure adapted with permission from^[^ ^136^ ^]^ under the Creative Commons CC BY license.

The exploration of magneto‐thermoelectric materials dates back to the 20th century, with Bi‐rich Bi_1‐x_Sb_x_ alloys being among the most extensively studied systems. Notably, Bi_1‐x_Sb_x_ was the first experimentally discovered 3D TI in 2003.^[^ ^135^ ^]^ The historical record for thermoelectric performance in these materials is a *zT* value of 1.28 at ≈225K under a magnetic field of 1.7 T. More recently, Pan Yu et al. reported a large magneto‐Seebeck effect and an enhanced *zT* of 1.7±0.2 at 180K and 0.7 T in high‐quality single‐crystalline Bi_88_Sb₁₂.^[^ ^136^ ^]^ The electronic structure of Bi_88_Sb₁₂ exhibits a direct bandgap with extrema at the L point (**Figure** **13**). The band structure of Bi_1‐x_Sb_x_ is highly composition‐dependent: for x<0.22, increasing Sb content induces a transition from a semimetal to a TI. At x≈4%, the L_s_ and L_a_ bands close, forming a fourfold degenerate Dirac point. As x increases to≈7%, the overlap between the T and L bands disappears, leading to an inverted‐band insulator, and at higher x, the system evolves into a direct‐gap TI (Figure 13b). Figure 13c illustrates the Fermi pockets in the Brillouin zone at E = 20 meV, revealing three highly anisotropic electron pockets at the L point. The small Fermi surface results in a low carrier concentration, while the pronounced anisotropy enhances carrier mobility and magneto‐thermoelectric performance along the trigonal direction. Theoretical calculations based on a massive Dirac Hamiltonian suggest that the tunability of the *S* under an external magnetic field originates from Zeeman splitting and the large g‐factor of Bi₈₈Sb₁₂. The large g‐factor induces a significant splitting of the Dirac bands into two distinct Fermi pockets with different Fermi energies (Figure 13d), each contributing separately to the magneto‐Seebeck effect. Experimental measurements show that the *S* is significantly enhanced under a weak magnetic field as low as 0.1 T (Figure 13e). The magneto‐Seebeck effect and magneto‐resistivity are generally described by:α = α_∞_μ^2^ *B* ^2^/(1 + μ^2^ *B* ^2^) + α_0_/(1 + μ^2^ *B* ^2^) and ρ = ρ_0_(1 + μ^2^ *B* ^2^), where *α*_0_ and *ρ*_0_ is the Seebeck coefficients and resistivity at zero magnetic fields, *α*_∞_ represents the saturation magneto‐Seebeck coefficient without any scattering, respectively. In high‐mobility topological insulators, an external magnetic field significantly enhances the *S* and resistivity while reducing electronic thermal conductivity. When these effects—Seebeck enhancement, electronic thermal conductivity suppression, and resistivity increase—reach an optimal balance, the power factor and *zT* can be maximized. Owing to its Dirac band dispersion and ultrahigh mobility, Bi₈₈Sb₁₂ achieves a remarkable magneto‐thermoelectric *zT* of ≈2 at 180K and 0.7 T.

**Figure 14:**
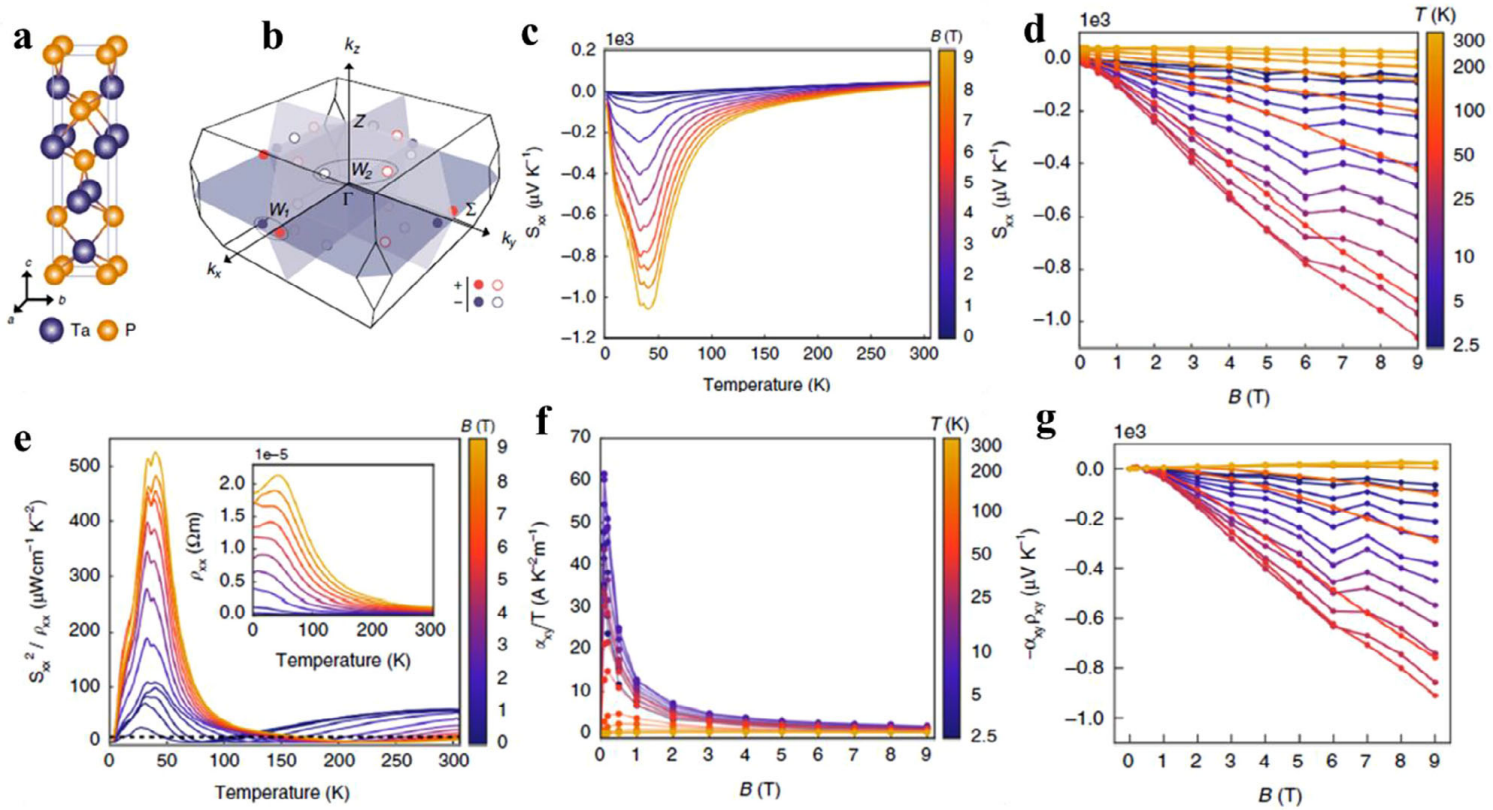
The high magneto‐thermoelectric performance of Weyl semimetal TaP. a) Inversion‐symmetry‐breaking crystal structure of TaP. b) The Brillouin zone of TaP, highlighting the locations of the inequivalent Weyl nodes W_1_ (filled circles) and W_2_ (empty circles). The Weyl nodes are paired as source “+” (red) and sink “−” (blue) of Berry curvature, separated in momentum space. c) Measured temperature‐dependent longitudinal thermopower *S*_xx_ at various magnetic fields for a topological Weyl semimetal (WSM) TaP.^[^ ^139^ ^]^ d) *S*_xx_ as a function of *B*, and the *S*_xx_ shows a non‐saturating linear relationship with the field. e) The power factor and the resistivity (inset)as a function of temperature.^[^ ^139^ ^]^ f) α_xy_/T (α_xy_ is the transverse thermoelectric conductivity) as a function of the magnetic field at various temperatures. g) The transverse component ‐α_xy_ρ_xy_ (thermoelectrical Hall conductivity) as the dominant contribution to logitudinal *S*_xx_. Figure adapted with permission from^[^ ^139^ ^]^ under the Creative Commons CC BY license.

The enhanced magneto‐thermopowers have also been found in some topological semimetals, such as Pb_1‐x_Sn_x_Se,^[^ ^137^ ^]^ ZrTe_5_,^[^ ^32^, ^138^ ^]^ TaP,^[^ ^139^ ^]^ Cd_3_As_2_,^[^ ^140^, ^141^ ^]^ ZrSiS,^[^ ^142^ ^]^ and PtSn_4_.^[^ ^143^ ^]^ For example, TaP, a Weyl semimetal with inversion‐symmetry‐breaking crystal structure (**Figure** **14**a,b), has an unusual energy‐independent DOS $g\left(n=0\right)=\frac{N_{f}\cdot Be}{4\pi^{2}\hbar^{2}\nu_{F}}$, which increases linearly with *B*. As a result, the *S*_xx_ of TaP (Figure 14c) increases by more than two orders of magnitude, starting at a B of≈0.1 T and reaching an ultra‐high value of≈1100 µV K^−1^ at cryogenic temperatures (40 K) and *B* = 9 T.^[^ ^139^ ^]^ Notably, *S*_xx_ rises linearly with B, without any sign of saturation (Figure 14d). More importantly, the low electrical resistivity *ρ*_xx_, is maintained owing to *n* = 0 Landau level (LL) with topologically protected Weyl nodes, allowing the electrons to evade electron localization under field. The resulting giant power factor (*S*_xx_^2^/*ρ*), reaching up to 525 µW cm^−1^ K^−2^ (Figure 14e), is an order of magnitude higher than that of conventional TE materials, which typically only work at elevated temperatures. The thermoelectric Hall conductivity (*α*_xy_) of TaP shows a universal quantization behavior of *α*_xy_/*T* (Figure 14f), which is independent of B‐field. The *S*_xx_ can be de decomposed into transverse (‐*α*_xy_ *ρ*_xy_) and longitudinal (*α*_xx_ *ρ*_xx_) component, namely,*S_xx_* = −α_*xy*_ρ_*xy*_ + α_*xx*_ρ_*xx*_. As shown in Figure 14g, we can see that the transverse term dominates the longitudinal *S*_xx_. Thus, the large magneto‐Seebeck can be attributed to the quantized thermoelectric Hall effect. However, the intrinsic *κ*_L_ in these novel semimetals is several orders of magnitude higher than in high‐performance TE semiconductors, limiting their overall TE efficiency.

**Figure 15:**
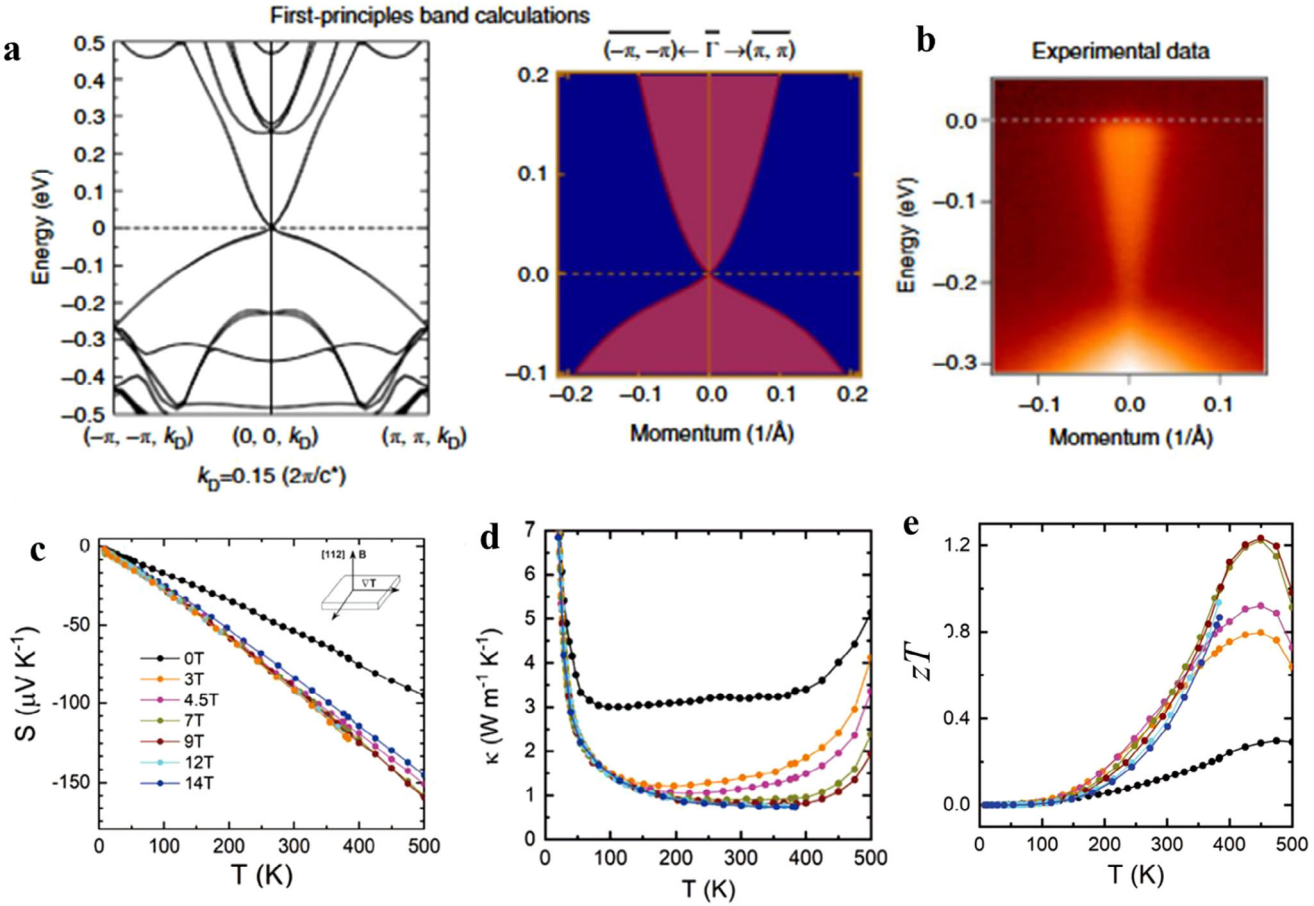
Band structure and magneto‐thermoelectric performance of the topological semimetal Cd_3_As_2_. a) Left: First‐principles calculation of the bulk electronic structure of Cd_3_As_2_. Right: Projected bulk band structure on to the (001) surface, where the shaded area represents the projection of the bulk bands.^[^ ^144^ ^]^ b) Angle‐resolved photoemission spectroscopy (ARPES) dispersion map of Cd₃As₂, exhibiting a simple Dirac cone‐like band structure. Figure a and b adapted with permission from^[^ ^144^ ^]^ under the Creative Commons CC BY license. c) Temperature dependence of thermopower for Cd_3_As_2_ under various magnetic field. d) Temperature‐dependent figure of merit (*zT*) of Cd_3_As_2_ under various magnetic fields.^[^ ^140^ ^]^ e) Thermal conductivity of Cd₃As₂ under different magnetic fields. Figure c–e adapted with permission.^[^ ^140^ ^]^ Copyright 2019, Wiley.

**Figure 16:**
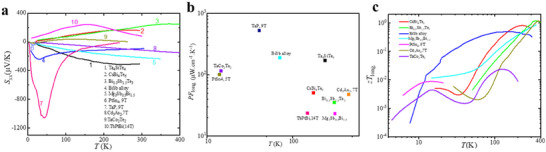
Longitudinal thermopower, power factor, and figure of merit of topological materials and other semiconductor materials at low temperatures. a) Temperature dependence of longitudinal magneto‐thermopower or thermopower (*S*_xx_). b) power factor (PF_long_) and c) The figure of merit (*zT*_long_) for topological materials (including Ta_4_SiTe_4,_^[^ ^120^ ^]^ PtSn_4_,^[^ ^143^ ^]^ TaP,^[^ ^139^ ^]^ Cd_3_As_2_,^[^ ^140^ ^]^ TaCo_2_Te_2_,^[^ ^148^ ^]^ Bi‐Sb alloy,^[^ ^149^ ^]^ Bi_0.5_Sb_1.5_Te_3_^[^ ^83^ ^]^ and other material such as CsBi_4_Te_6_.^[^ ^150^ ^]^

For high‐performance magneto‐thermoelectric applications, a material must exhibit large magneto‐thermopower and low *κ*_L_, while maintaining high σ under a magnetic field. A notable example is Cd_3_As_2_, a 3D topological Dirac semimetal with a highly linear bulk band crossing forming a 3D dispersive Dirac cone (**Figure** **15**a,b).^[^ ^144^ ^]^ Cd_3_As_2_ possesses ultrahigh carrier mobility (𝜇≈10^4^≈10^6^ cm^2^V^−1^s^−1^),^[^ ^145^ ^]^ which is highly favorable for TE performance. The thermopower *S*_xx_ shows a significant increase with increasing *B* (Figure 15c). Moreover, its complex crystal structure and soft phonon modes contribute to an anomalously low *κ*_L_ (0.3 to 0.7 W m^−1^ K^−1^ at 300K).^[^ ^146^, ^147^ ^]^ In Cd_3_As_2_ crystal, the κ_e_ dominates the total *κ*, and hence, the magnetic field significantly suppresses heat transport (Figure 15d). As a result, Cd_3_As_2_ shows a high *zT* of 1.24 at 350K under a magnetic field of up to 7 T (Figure 15e), comparable to the best room‐temperature TE material Bi_2_(Te,Se)_3_ system.^[^ ^140^, ^141^ ^]^ **Figure** **16** summarize the longitudinal thermoelectric performance of some topological materials^[^ ^83^, ^120^, ^139^, ^140^, ^143^, ^148^, ^149^ ^]^ and other materials^[^ ^150^ ^]^ at low temperature. The results suggest that topological semimetals hold significant potential for applications in cryogenic temperature.

#### 2.11.2 Transverse Nernst Effects in Topological Materials

**Figure 17:**
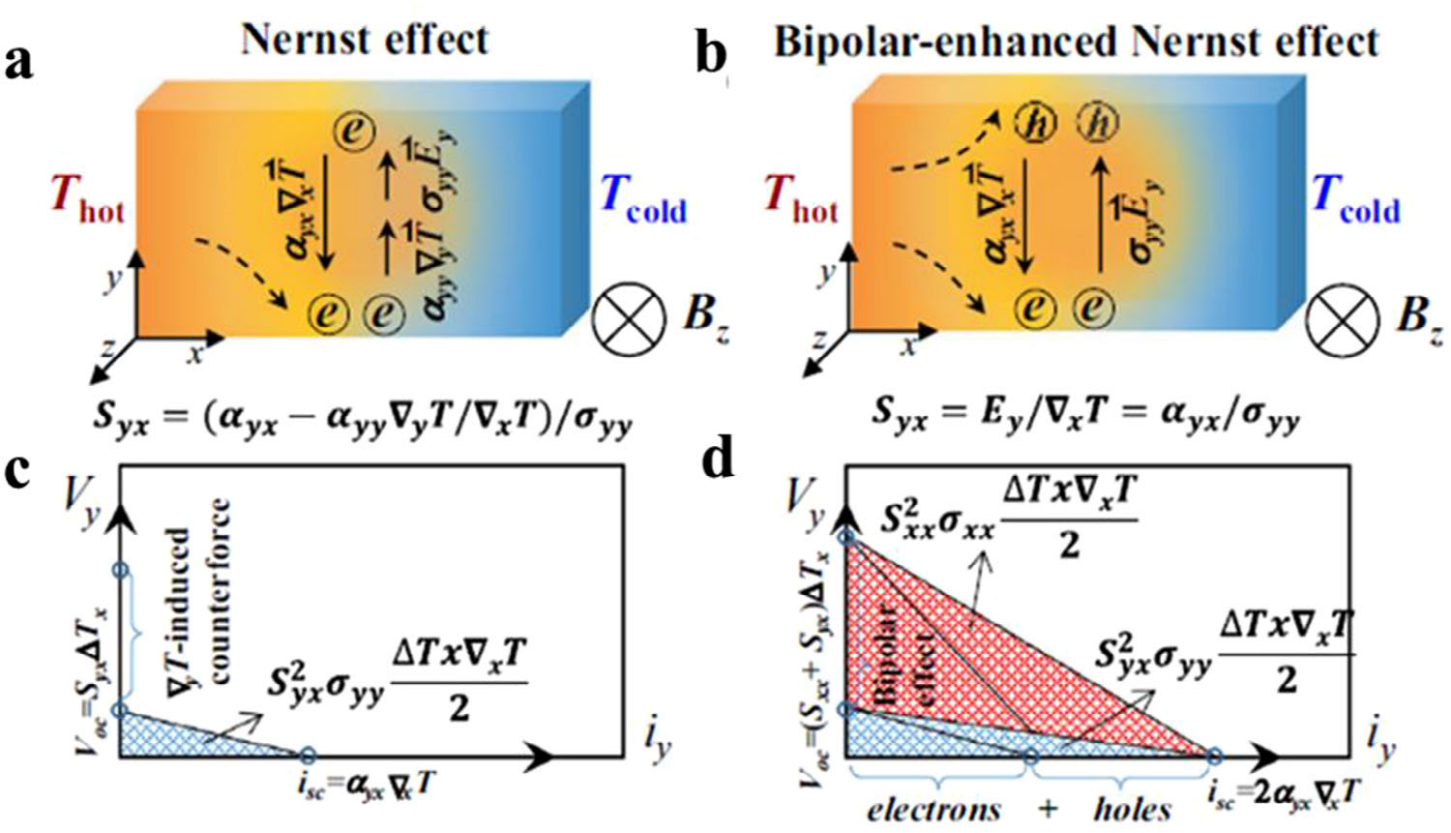
Bipolar effect on the Nernst effect. a,c) Schematic of Nernst effect in single‐type carrier system without bipolar effect, from perspectives of internal current flow (top) and electric power output (bottom). The half of the product of the short‐circuit current (i_sc_ = α_xx_∇_x_T, where α is the Seebeck conductivity) and open‐circuit voltage (V_oc_ = *S*_xx_Δ*T*_x_, where *S i*s the Seebeck coefficient, represents the maximal electric power output measured by the power factor *(PF_trans_*.), as indicated by the shaded areas. Strong bipolar effect can greatly enhance the transverse Nernst power factor. Note that d) depicts the ideal case of a full compensation of the thermal Hall effect for arbitrary materials, corresponding to perfectly symmetric conduction and valence bands with the Fermi level located at the midpoint. Figure adapted with permission from^[^ ^150^ ^]^ under the Creative Commons CC BY license.

The Nernst‐Ettingshausen effect is known as a transverse magneto‐thermoelectric effect which occurs when carriers in a conductor produce a transverse electrical signal (E_y_) in response to a perpendicular magnetic field (*B_z_*) and a longitudinal thermal gradient (∇*T*_x_). The Nernst thermopower S_yx_ is defined as $S_{\mathrm{yx}}=-E_{y}/\nabla T_{x}=\frac{V_{y}}{\Delta T_{x}}\frac{L_{x}}{L_{y}}$, where *L*_x_ and *L*_y_ denote the distance between the two temperature leads and the distance between the two voltage wires, respectively. For non‐magnetic materials with a single type of charge carriers, the Nernst thermopower exhibits linear behavior as a function of the magnetic field. However, in semimetals where both electrons and holes coexist, the magnetic field dependence of ordinary Nernst effect (ONE) becomes non‐linear. Typically, the ONE is generally small in normal metals with a single type of chare carries due to the Sonderheimer's cancellation. As shown in **Figure** **17**a–c, within a single‐type carrier system, the transverse thermoelectric current α_*yx*_∇_*x*_ *T* is balanced by two main factors to reach a steady state: (i) the current induced by the transverse temperature gradient α_*yy*_∇_*y*_ *T*, related to the thermal Hall effect; (ii) the current driven by the Nernst effect σ_*yy*_ *E_y_*, crucial for electric power generation and heat pumping. To enhance the Nernst field (*E_y_*), reducing the impact of the α_*yy*_∇_*y*_ *T* term is necessary, making the bipolar effect an effective solution. To achieve this, the bipolar effect emerges as a viable choice. Ideally, as shown in Figure 17b,d, the α_*yy*_∇_*y*_ *T* component is eliminated in two carrier system with a perfect compensation of electrons and hole.

**Figure 18:**
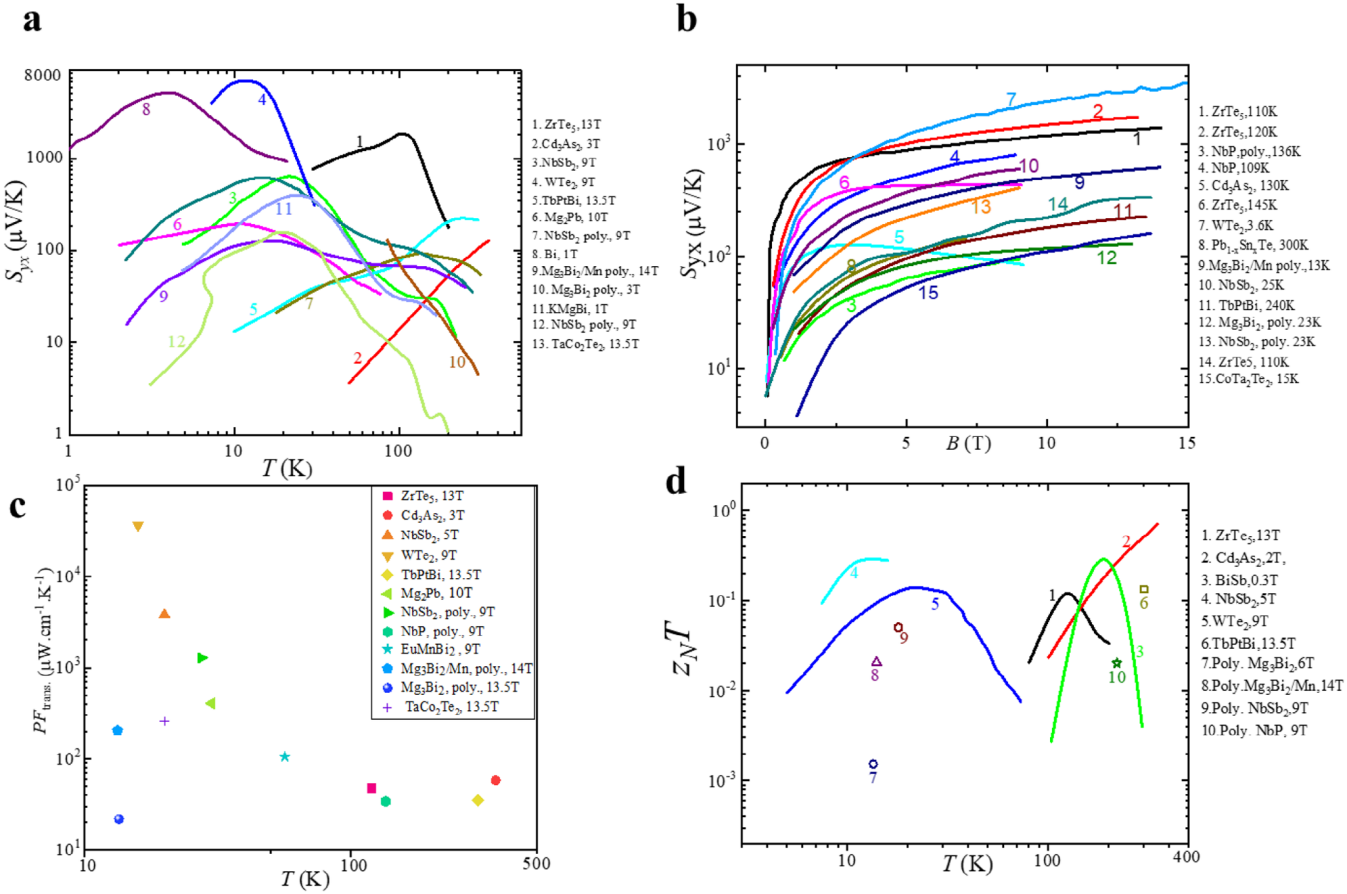
Transverse thermopower, power factor, and figure of merit of topological materials. a) Temperature dependence of the Nernst‐based thermopower (*S*_xy_). b) The magnetic‐field‐dependent Nernst thermopower (*S*_xy_). c) Transverse peak power factors. d) Transverse figure of merit (*z*_N_ *T*) for topological materials (ZrTe_5_,^[^ ^32^, ^138^, ^152^ ^]^ NbP polycrystalline,^[^ ^34^ ^]^ Bi‐Sb alloys,^[^ ^168^ ^]^ Mg_2_Pb,^[^ ^162^ ^]^ Cd_3_As_2_,^[^ ^151^ ^]^ Bi,^[^ ^156^ ^]^ Mg_3_Bi_2_ polycrystalline,^[^ ^95^ ^]^ Mg_3_Bi_2_/Mn polycrystalline,^[^ ^159^ ^]^ NbSb_2_,^[^ ^157^ ^]^ TbPtBi,^[^ ^161^ ^]^ KMgBi,^[^ ^163^ ^]^ WTe_2_^[^ ^166^ ^]^ Pb_1‐x_Sn_x_Te^[^ ^137^ ^]^ EuMnBi_2_,^[^ ^165^ ^]^ NbSb_2_ polycrystalline^[^ ^158^ ^]^ and TaCo_2_Te_2_).^[^ ^148^ ^]^

Therefore, semimetals with a strong bipolar effect are appealing for generating a large Nernst effect. In particular, topological semimetals with linear band dispersion possess key attributes for a large *S*_yx_ (*B_z_*) such as high cyclotron frequency, weak scattering characterized by *ω*_c_ *τ* > 1 (where *ω*_c_ is the cyclotron frequency and *τ* the relaxation time),^[^ ^151^ ^]^ ultra‐high carrier mobility, strong Berry curvature, as well as strong bipolar transport of compensated electrons and holes.^[^ ^152^ ^]^ Many topological semimetals feature these properties, leading to high electrical conductivity and large magnetoresistance, even at low external magnetic fields.^[^ ^153^ ^]^ Many nonmagnetic topological semimetals have been experimentally demonstrated to exhibit promising Nernst thermopowers, such as NbP,^[^ ^34^, ^154^ ^]^ WTe_2,_^[^ ^155^ ^]^ Cd_3_As_2,_^[^ ^151^ ^]^ Pb_1−_ *_x_* Sn*_x_* Se,^[^ ^137^ ^]^ *Z*rTe_5_,^[^ ^32^, ^138^, ^152^ ^]^ Bi,^[^ ^156^ ^]^ NbSb_2_,^[^ ^157^, ^158^ ^]^ Mg_3_Bi_2_,^[^ ^159^, ^160^ ^]^ TbPtBi,^[^ ^161^ ^]^ Mg_2_Pb,^[^ ^162^ ^]^ KMgBi,^[^ ^163^ ^]^ NbAs_2_.^[^ ^164^ ^]^ **Figure** **18** summarizes transverse thermopower (*S*_yx_), power factor (*PF*_trans._), and Nernst figure of merit (*Z*_N_ *T*) topological materials^[^ ^34^, ^120^, ^139^, ^140^, ^143^, ^151^, ^152^, ^156^, ^158^, ^159^, ^162^, ^165^, ^166^ ^]^ for low temperature application. As shown in Figure 18, most of topological semimetals show large transverse Nernst effects below room temperature. Notably, the maximum Nernst thermopower(*S*_yx_) occurs at the temperature where the chemical potential reaches the Dirac points.^[^ ^154^ ^]^ This is because a Dirac point is not only the location of the minimum DOS, but also where holes and electrons are compensated.^[^ ^167^ ^]^

The *S*_yx_ can be quantified by the following equation:

(6)

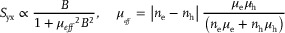




**Figure 19:**
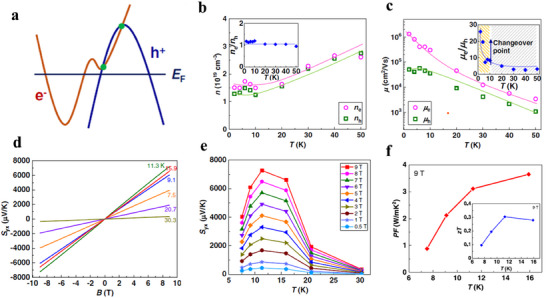
High transverse Nernst‐based thermoelectric performance in WTe_2_ topological semimetals. a) Schematic of the band structure of WTe_2_ Weyl semimetal. Showing nearly perfect compensation between electrons and holes at the Fermi energy. The green dots represent a pair of Weyl points above the Fermi energy. b) Carrier concentration. c) Mobility of both electrons and holes. The insets in (b,c) present the ratios of the concentration and mobility of electrons to holes, respectively. d) Nernst signal measured from 7.5 to 30.3K. e) Nernst signal as a function of temperature. f) Temperature dependence of Nernst power factor of WTe_2_, inset shows the *Z_N_T* values.Figure adapted with permission from^[^ ^163^ ^]^ under the Creative Commons CC BY license.^[^ ^155^ ^]^

The ultrahigh mobility from the linear band dispersion, along with the compensation between electrons and holes, is responsible for the large ONE, as observed in WTe_2_.^[^ ^155^ ^]^ WTe_2_ has a unique band structure (**Figure** **19**a) that supports nearly compensated electrons and holes with extremely high mobilities. As shown in Figure 19b, electrons (n_e_) and holes (*n*_h_) carrier concentration are nearly equal across the measured temperature range, with a slight imbalance at higher temperatures, while the mobility of the electrons is slightly higher than that of the holes, particularly at low temperature. This compensation is a key feature of the type‐II Weyl semimetal WTe_2_ and plays a crucial role in its exceptional transverse thermopower (Figure 19d,e). Topological semimetals typically exhibit giant magnetoresistance while maintaining resistivity comparable to conventional semiconductors due to their gapless band structures. This makes them highly desirable for achieving a large Nernst power factor ($PF_{trans.}=S_{yx}^{2}\sigma_{yy}$). As shown in Figure 19f, WTe_2_ achieves a Nernst power factor of ≈3 × 10^4^ µW cm^−1^K^−2^ and a promising Nernst figure of merit ($z_{N}T=S_{yx}^{2}\sigma_{yy}/\kappa_{xx}$) of 0.3 at 11.3K and 9 T. Such a high *PF_trans._* are 2–3 orders of magnitude higher than the conventional longitudinal TE power factor values, and the *z*_N_ *T* of WTe_2_ is comparable to conventional longitudinal TE materials. Moreover, the strong bipolar effect and high carrier mobility in Dirac and Weyl semimetals are particularly beneficial for achieving a large Nernst effect even under low magnetic fields. For instance, a large *z_N_T* of 0.5 at ≈300K has been observed in Cd_3_As_2_ in a modest field of just 2T,^[^ ^151^ ^]^ which can be generated using a standard commercial permanent magnet.

In addition to that, Weyl semimetals demonstrate both the ordinary Nernst effect resulting from the diffusion of electrons and holes in an external magnetic field, and the anomalous Nernst effect (ANE), which arises from the nonzero Berry curvature at the Weyl nodes. The ANE contributes to a rapid increase in *S*_yx_ at low fields and has been observed in some topological semimetals.^[^ ^138^, ^145^, ^169^, ^170^, ^171^, ^172^ ^]^

#### 2.11.3 Transverse Anomalous Nernst Effects in Topological Magnets

A magnetic material with a temperature gradient can produce a transverse electric field even in the absence of a magnetic field. This response, known as the ANE, is particularly promising for applications in transverse TE devices, as it does not rely on large external magnetic fields.

Compared with trivial magnets, the ANE in topological magnets does not follow the general magnetization scaling relation $S_{\mathrm{xy}}^{A}=\left|N_{\mathrm{xy}}^{A}\right|\mu_{0}M$, where *M* is the magnetization and $N_{\mathrm{xy}}^{A}$ is the anomalous Nernst coefficient. The ANE is the thermoelectric counterpart of the anomalous Hall effect (AHE). The corresponding transverse anomalous Nernst conductivity ($\mathrm{ANC},\alpha_{xy}^{A}$) is related to the energy derivative of anomalous Hall conductivity (AHC, $\;\sigma_{xy}^{A}$) via the Mott relation:^[^ ^36^ ^]^

(7) $$\alpha_{xy}^{A}=\frac{\pi^{2}}{3}\frac{k_{B}^{2}T}{e}{\left[\frac{d\sigma_{xy}^{A}}{dE}\right]}_{E_{F}}$$

where *k*_B_ is the Boltzmann constant, and e is the the elementary charge. Both $\;\sigma_{xy}^{A}$ and $\alpha_{xy}^{A}$ originate from the Berry curvature effects in topological systems.^[^ ^173^, ^174^ ^]^

(8) $$\sigma_{xy}^{A}=-\frac{e^{2}}{\hbar }\int \frac{dk}{{\left(2\pi \right)}^{3}}\sum_{n}f_{nk}\Omega_{n,xy}\left(k\right)$$

(9) $$\begin{array}{ccc} \alpha_{xy}^{A} & = & \frac{e}{T\hbar }\int \frac{dk}{{\left(2\pi \right)}^{3}}\sum_{n}\Omega_{n,xy}\left(k\right)\\ & & \left\{\left[\varepsilon_{n}\left(k\right)-\mu \right]f_{nk}+k_{B}T\ln\left(1+\exp\left\{-\beta \left[\varepsilon_{n}\left(k\right)-\mu \right]\right\}\right)\right\} \end{array}$$

where *f_n,k_*, *ɛ_n_*, *ħ*, and *μ* are the Fermi‐Dirac distribution function, energy of the *n* ^th^ band, the reduced Planck's constant, and the chemical potential, respectively, and *Ω*(***k***) is the Berry curvature, i.e., the Berry phase per unit area in ***k*** space.

The anomalous Nernst thermopower $S_{xy}^{A}$ is related to both ANC and AHC values and consists of two components,^[^ ^175^ ^]^ $S_{xy}^{A}=\alpha_{xy}^{A}\rho_{xx}-\sigma_{xy}^{A}\rho_{xx}S_{xx}$, where *α^A^*_xy_, *ρ_xx_*, *σ^A^_xy_*, and *S_xx_* are the anomalous Nernst conductivity, longitudinal resistivity, Hall conductivity, and Seebeck coefficient, respectively. The first term is the contribution of the transverse TE current, and the second term is due to the Hall current induced by Seebeck effect. *α^A^*_xy_ directly converts ∇*T* into a transverse electric field, making the first term typically regarded as the intrinsic ANE.^[^ ^176^ ^]^

**Figure 20:**
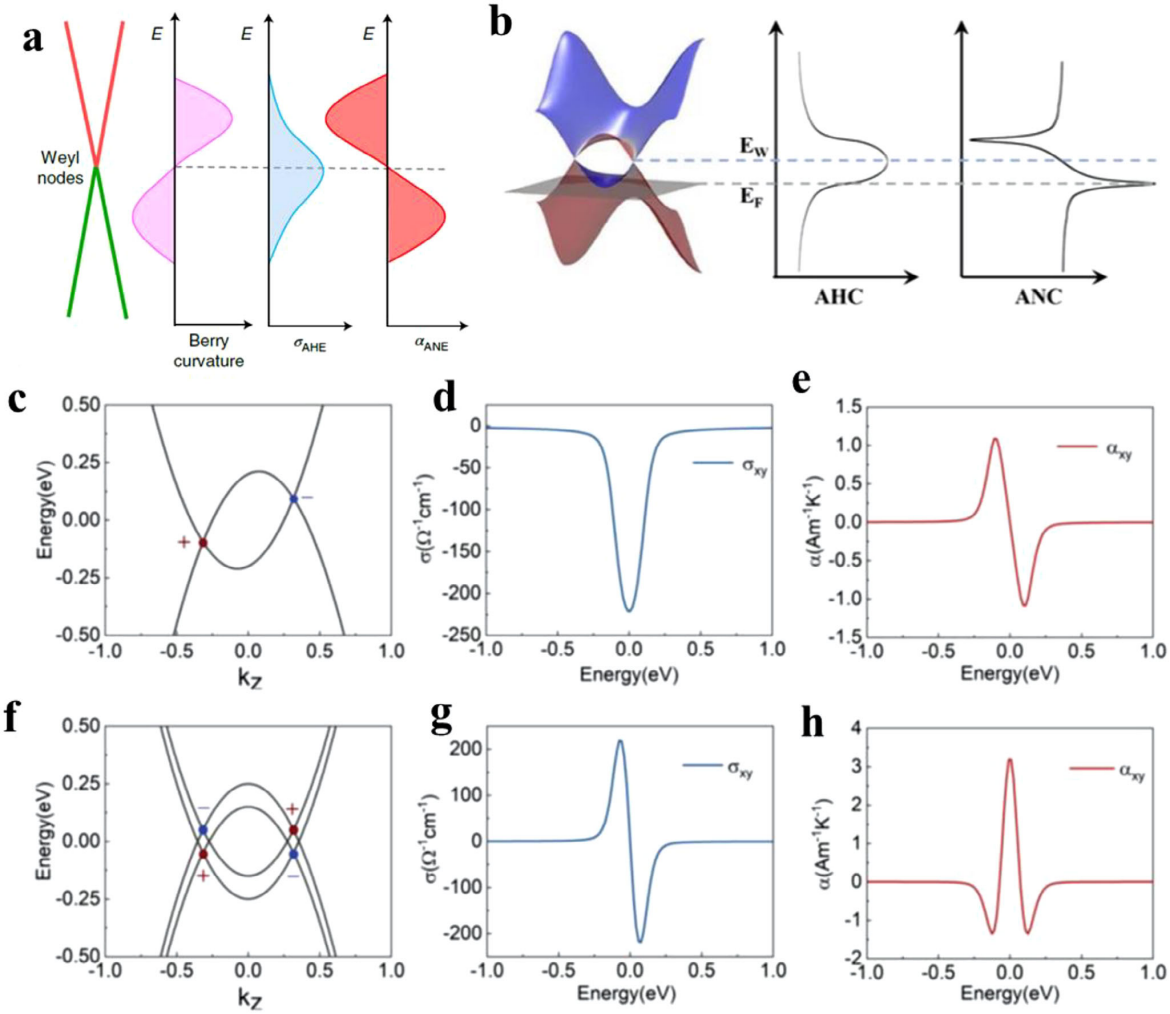
Anomalous Nernst effect in magnetic Weyl semimetals. a) Schematic of the *E*_F_ dependence of the Berry curvature, anomalous Hall conductivity (AHC or *σ*_AHE_) and anomalous Nernst conductivity (ANC or *α*_ANE_). a) Adapted with permission from^[^ ^177^ ^]^ under the Creative Commons CC BY license. b) The maximum AHC and ANC occur at different energy levels: the maximum AHC is observed when *E*_F_ is near the Weyl nodes, while the maximum ANC occurs at an energy away from the Weyl nodes (https://www.cpfs.mpg.de). c) Band structure of the two Dirac band model in magnetic Weyl semimetal. d) Anomalous Hall conductivity (AHC) with respect to the chemical potential for the two Dirac band model. e) Anomalous Nernst conductivity (ANC) with respect to the chemical potential for the two Dirac band model. f) Band structure of a four‐band Dirac model under the Zeeman field. g) AHC and h) ANC with respect to the chemical potential for the four Dirac band model. A pair of Dirac nodes under a Zeeman field lead to an large ANC compared with that of a simple Weyl semimetal with two Weyl nodes. c‐h) Adapted with permission from^[^ ^178^ ^]^ under a Creative Commons CC BY license.

In magnetic Weyl semimetals, Weyl nodes appear in pairs in momentum space, possessing positive or negative magnetic charges that act as sources or sinks of the fictitious magnetic field or Berry curvature.^[^ ^174^ ^]^ Mott relation indicates that $\sigma_{xy}^{A}$ is predominantly governed by the average Berry curvature over all occupied states, while $\alpha_{xy}^{A}$ depends on the Berry curvature or energy‐dependent $\sigma_{xy}$ only near the Fermi level. The strength of the Berry curvature is directly related to the position of the Fermi level (*E*_F_), with the maximum anomalous Hall conductivity (*σ*_AHE_) when *E*_F_ is near the Weyl nodes, while the maximum anomalous Nernst effect (*α*_ANE_) is observed at a different energy (**Figure** **20**a). Thus, the *E*_F_ needs to be tuned to align with the Weyl points for a maximum *σ*_AHE_, and set away from the Weyl points to maximize *α*_ANE_ (Figure 20b).

Figure 20c illustrates a tilted two‐band Weyl mode. In this model, the calculated AHC (Figure 20d) is an even function with respect to the chemical potential, while the corresponding ANC (Figure 20e) curve is an odd function. To enhance ANC, a system can be designed with four Weyl nodes arranged in a “G‐type” chirality configuration, where one pair of nodes is positioned below the Fermi level and the other above it (Figure 20f).^[^ ^178^ ^]^ This configuration would produce a double peak in the AHC (Figure 20g), leading to a large ANC peak at the Fermi level (Figure 20h),^[^ ^178^ ^]^ contrasting with the simpler Weyl semimetal model. Such a system can be realized by applying a Zeeman field that splits the Dirac nodes and breaks the time‐reversal symmetry.

**Figure 21:**
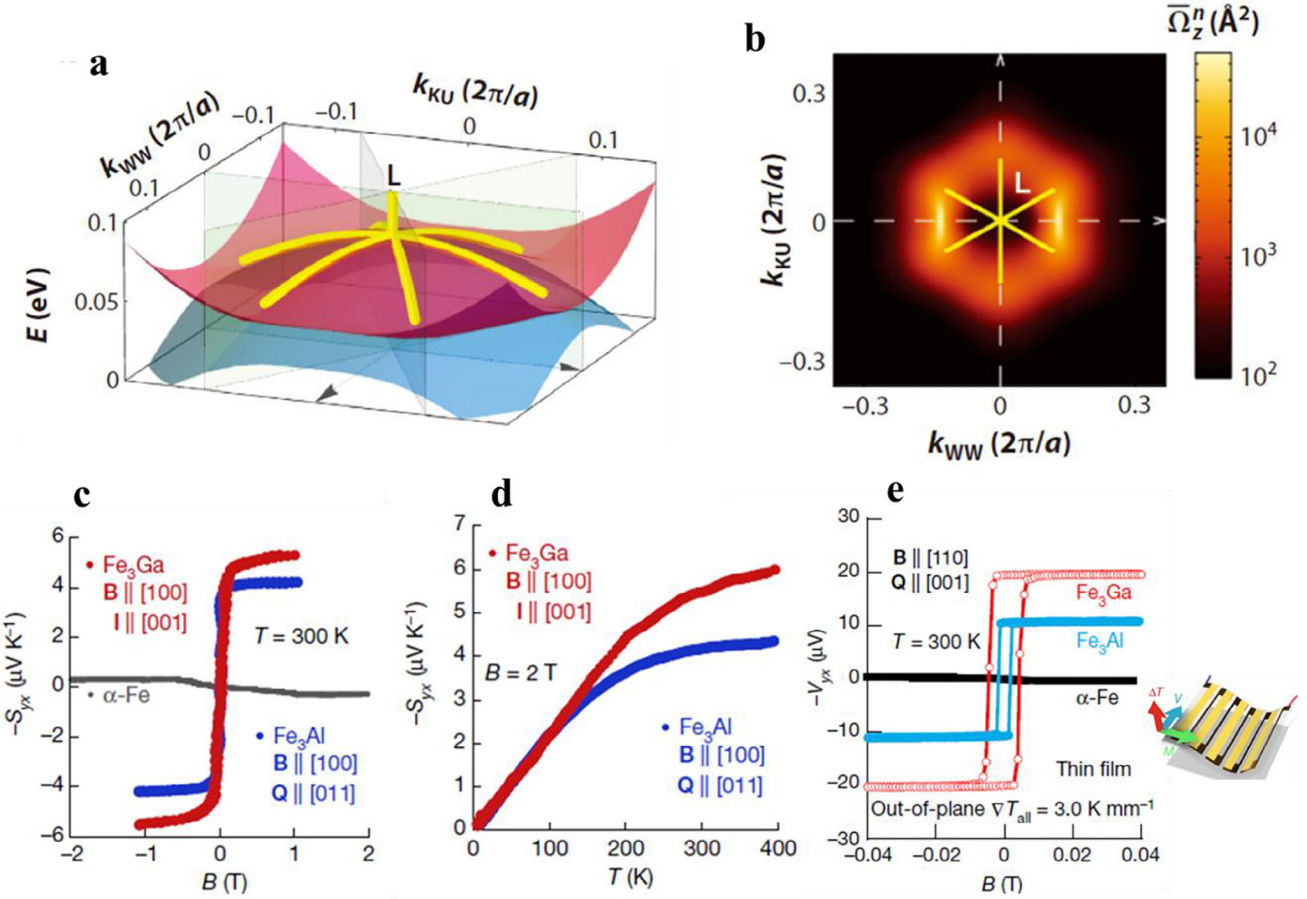
Anomalous Nernst‐based thermoelectric performance of Fe_3_X (X = Ga, Al) topological magnets. a) The nodal web structure of Fe_3_Ga, consists of nodal lines (yellow), which exhibit a nearly flat energy dispersion. b) Contour map of the Berry curvature with enhanced regions around nodal lines (yellow lines).^[^ ^179^ ^]^ c). Field dependence of the anomalous Nernst thermopower for Fe_3_Ga and Fe_3_Al. d) Temperature dependence of anomalous Nernst thermopower for Fe_3_Ga and Fe_3_Al. e) Field dependence of the transverse voltage for Fe_3_Ga, Fe_3_Al, and α‐Fe thin film devices.^[^ ^179^ ^]^ The inset shows a flexible thermopile based on the ANE.Figure adapted with permission.^[^ ^179^ ^]^ Copyright 2020, Springer Nature.

Fe_3_X (X = Ga, Al), for example, have a nearly flat band structure composed of interconnected nodal lines near the Fermi level, and strong Berry curvature around the nodal web (**Figure** **21**a,b). Such band structure results in a maximum anomalous Nernst thermopower (*S*_ANE_) of ∼6 µV K^−1^ at room temperature (Figure 21c,d). A transverse malleable TE generator has been designed using the Fe_3_Al and Fe_3_Ga thin films, as they feature in‐plane magnetization and exhibit spontaneous ANE even at zero field.^[^ ^179^ ^]^ This transverse TE generator demonstrates an output voltage in the range of several tens of microvolts (Figure 21e).

**Figure 22:**
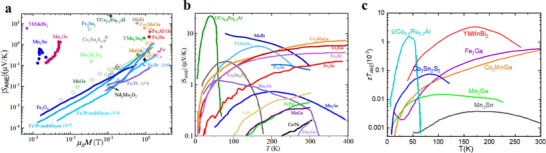
Anomalous Nernst‐based thermoelectric performance in various topological magnets. a) Magnetization (*M*) dependence of the maximum values of the ANE in various reported magnetic materials. The data sources were adopted from ref. [189] (before 2022), MnBi,^[^ ^190^ ^]^ YMn_6_Sn_6_,^[^ ^187^ ^]^ YbMnBi_2_,^[^ ^177^ ^]^ Fe_3_Sn_2_,^[^ ^185^ ^]^ and Mn_3_Si_2_Te_6_^[^ ^191^ ^]^ (after 2022). b) Temperature dependence of the ANE coefficient for various magnets. The data sources were adopted from ref. [189] (before 2022), MnBi^[^ ^190^ ^]^ and YbMnBi_2_^[^ ^177^ ^]^ (after 2022).c) The temperature dependence of *zT*_ANE_ values of several topological magnets. The data was adopted from ref. [177].

**Figure** **22**a,b provide a summary of the magnetization dependent and temperature dependent ANE values for various magnetic materials. In contrast to conventional strong ferromagnets (such as Co/Ni alloy), which exhibit ANE signals that scale roughly linearly with the spontaneous magnetization, topological magnets—including Heusler compounds (e.g., Co_2_MnGa,^[^ ^180^, ^181^ ^]^ Fe_2_YZ(Y = Co,Ni, Z = Al, Ga)),^[^ ^182^ ^]^ Kagome compounds (e.g., Co_3_Sn_2_S_2_,^[^ ^33^, ^183^, ^184^ ^]^ Fe_3_Sn_2_,^[^ ^185^ ^]^ RMn_6_Sn_6_(R = Tb,Gd,Tm,Lu),^[^ ^186^, ^187^ ^]^ AV_3_Sb_5_(A = K,Rb,Cs)),^[^ ^188^ ^]^ iron‐based binary compounds (e.g., Fe_3_Al,^[^ ^179^ ^]^ Fe_3_Ga,^[^ ^179^ ^]^ and Fe_3_Sn),^[^ ^189^ ^]^ pnictide antiferromagnet (e.g.,YbMnBi_2_,^[^ ^177^ ^]^ MnBi),^[^ ^190^ ^]^ and Mn_3_Si_2_Te_6_^[^ ^191^ ^]^ —violate this conventional scaling relation. These topological magnets have large *S*_ANE_ values, often one or two orders of magnitude higher than expected, due to significant Berry curvature contributions from Weyl points or gapped nodal rings. Alongside Weyl ferromagnets, large ANE signals have also been observed in topological antiferromagnets with no net magnetization, such as Mn_3_Sn^[^ ^192^, ^193^, ^194^ ^]^ and YbMnBi_2_^[^ ^177^ ^]^ Compared to ferromagnets, antiferromagnets offer great benefits for practical transverse TE applications, such as high mobility, ultrafast dynamics, extremely low magnetization, and small magnetic disturbance. Figure 22c illustrates the temperature dependence of anomalous Nernst TE figure of merit (*zT*_ANE_), which shows a significantly lower magnitude than the conventional longitudinal TE figure of merit (*zT_long._*).

In addition to intrinsic Berry curvature effects, extrinsic mechanisms—such as magnon‐drag and skew scattering—can also contribute to ANE. This has been demonstrated in ferromagnetic MnBi, where these extrinsic factors significantly enhance ANE signals.^[^ ^190^ ^]^ When a temperature gradient is applied to the ferromagnetic material, heat is first transferred from the phonon system to the magnon system, thermally excited magnons that carry both linear momentum and spin angular momentum. The transfer of linear momentum (*p*) generates the magnon‐drag thermopower (*S*_MD_). In a material with strong spin‐orbit coupling (SOC) like MnBi, the transfer of spin angular momentum creates an out‐of‐equilibrium additional spin polarization of the conduction electrons. This spin polarization can then generate a transverse thermoelectric voltage via the inverse spin Hall effect, contributing to the observed giant ANE in MnBi. As a result, a cross‐plane ANE thermopower reaching 10 µV K^−1^ at 80 K under a 0.6 T magnetic field was realized in MnBi. The ANC value is outstanding compared to other magnetic materials, owing to the magnon‐mediated transport and low resistivity in MnBi.

#### 2.11.4 Topological Nernst Effects

**Figure 23:**
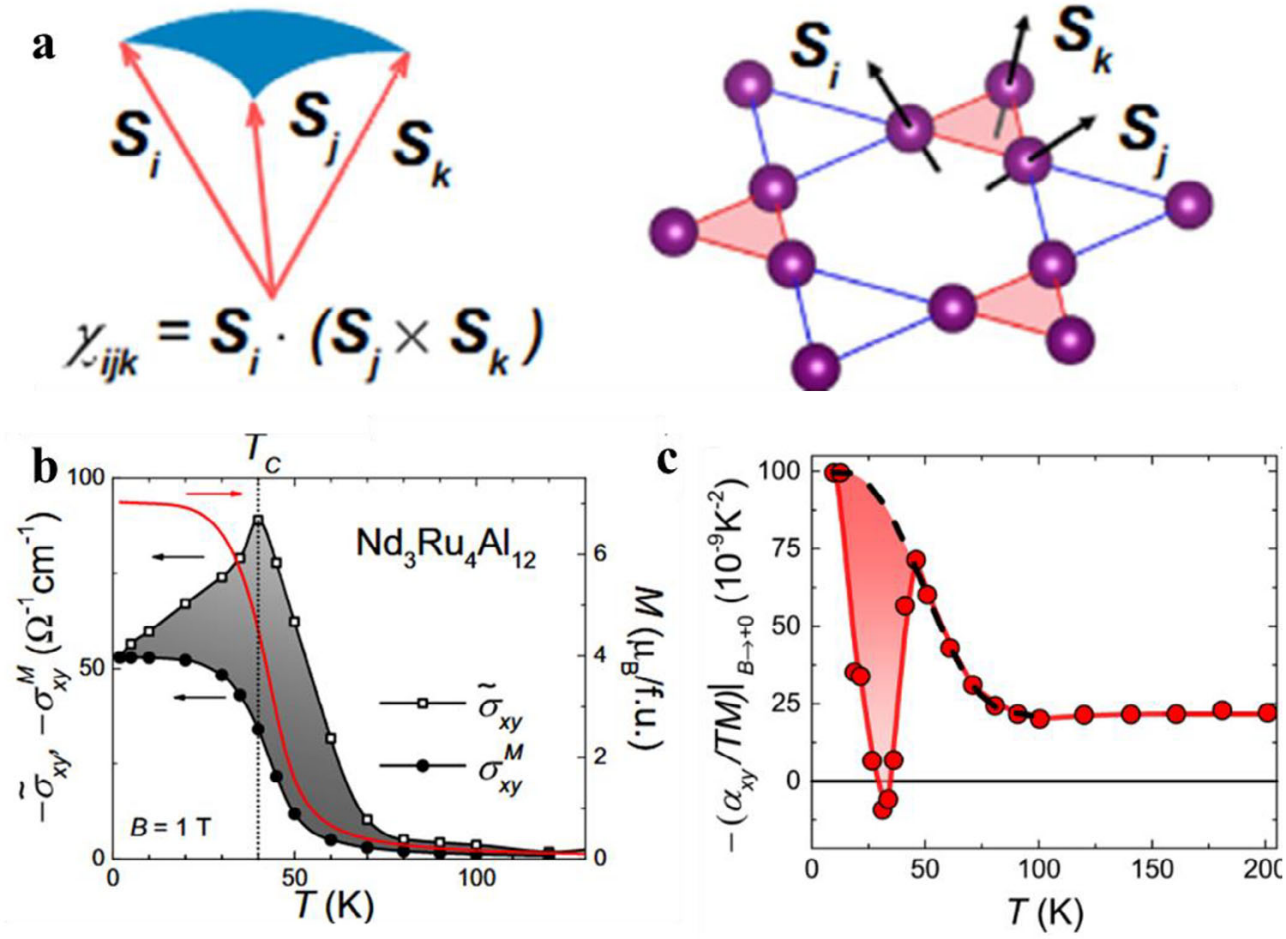
Topological Nernst effect in ferromagnetic Nd_3_Ru_4_Al_12_ material. a) Schematic illustration of the solid angle subtended by three non‐coplanar spins, resulting in a finite scalar spin chirality (SSC). b) Temperature dependence of anomalous Hall conductivity (≈σ_xy_) and magnetization‐dependent intrinsic anomalous Hall conductivity ($\sigma_{\mathrm{xy}}^{M}$) at *B* = 1 T. The shaded area stands for the SSC‐related Hall conductivity $\sigma_{\mathrm{xy}}^{\chi }=∼\sigma_{xy}-\sigma_{\mathrm{xy}}^{M}$. c) Temperature dependence of the quantity ${\left.\alpha_{xy}/\left(TM\right)\right|}_{B\to +0}$ provides an estimate for the magnetization‐proportional anomalous term ($\sigma_{\mathrm{xy}}^{M}$). The dashed black line represents a polynomial fit to the data at low temperatures and above T = 50K. A strong deviation from the main trend (shaded region) is visible close to the Curie temperature. Figure adapted with permission.^[^ ^201^ ^]^ Copyright 2021, PNAS license.

In addition to the normal Nernst effect, which is proportional to an external magnetic field, and the anomalous Nernst effect, which is proportional to spontaneous magnetization, a third contribution to Nernst signals exists—known as the topological Nernst effect (TNE). TNE is a thermoelectric counterpart of the topological Hall effect (THE) and has been observed in the skyrmion lattice phase or in chiral magnets, such as MnGe,^[^ ^195^ ^]^ Gd_2_PdSi_3_^[^ ^196^ ^]^ and Fe_3_Sn_2_.^[^ ^197^ ^]^ The origin of TNE can be attributed to the Berry phase in real space and associated with scalar spin chirality produced from non‐coplanar/topological spin textures.^[^ ^195^, ^198^ ^]^ These topological spin textures describe the intricate alignment of electron spins in chiral magnets, encompassing features like skyrmions and antiskyrmions.^[^ ^199^ ^]^ These textures have unique topological properties that are characterized by a quantity called the topological charge. The topological nontrivial spin texture can yield a Berry phase proportional to the scalar spin chirality **S**_1_ (**S**_2_×**S**_3_) of adjacent three spins (**Figure** **23**a). This leads to an emergence of additional fictitious magnetic field in real space. As a result, the propagating electrons will deflect and give an additional contribution of Nernst signals when the longitudinal temperature is applied. The topological Nernst conductivity is similarly related to the energy derivative of the topological Hall conductivity and follows the Mott relation. TNE can be identified by analyzing the field dependence of Nernst signals in the low‐field region and at temperatures where the skyrmion lattice is present. Its sign may differ from that of the magnetization‐dependent ANE, leading to a sign reversal in the total TNE signal at low magnetic fields. Moreover, TNE varies with temperature in proportion to the fictitious flux generated by scalar spin chirality.^[^ ^195^ ^]^ Recently, the topological semimetal Fe₃Sn₂ was found to exhibit a significant TNE at low temperatures, which is linked to the non‐zero spin chirality of the skyrmion bubble phase.^[^ ^200^ ^]^ Beyond materials with long‐range noncoplanar magnetic orders or topologically nontrivial spin textures (such as magnetic skyrmions), recent studies have revealed that thermal fluctuations near magnetic ordering transitions can also generate finite scalar spin chirality, leading to transverse transport properties such as Hall conductivity and the Nernst effect. The temperature‐dependent Hall conductivity and Nernst signal (Figure 23b,c) measured in Nd₃Ru₄Al₁₂ show a strong enhancement near the Curie temperature, which cannot be explained by conventional magnetization‐dependent models.^[^ ^201^ ^]^ The non‐monotonic behavior, featuring a pronounced minimum, suggests an additional spin‐chirality‐driven contribution to the Nernst effect, opposite in sign to the magnetization‐dependent anomalous Nernst effect. This indicates that spin chirality arising from momentum‐space Berry curvature contributes significantly to the Nernst effect.^[^ ^201^ ^]^

## 3 Potential Applications of Topological Thermoelectrics

### 3.1 Nernst‐Based Thermoelectric Generation

**Figure 24:**
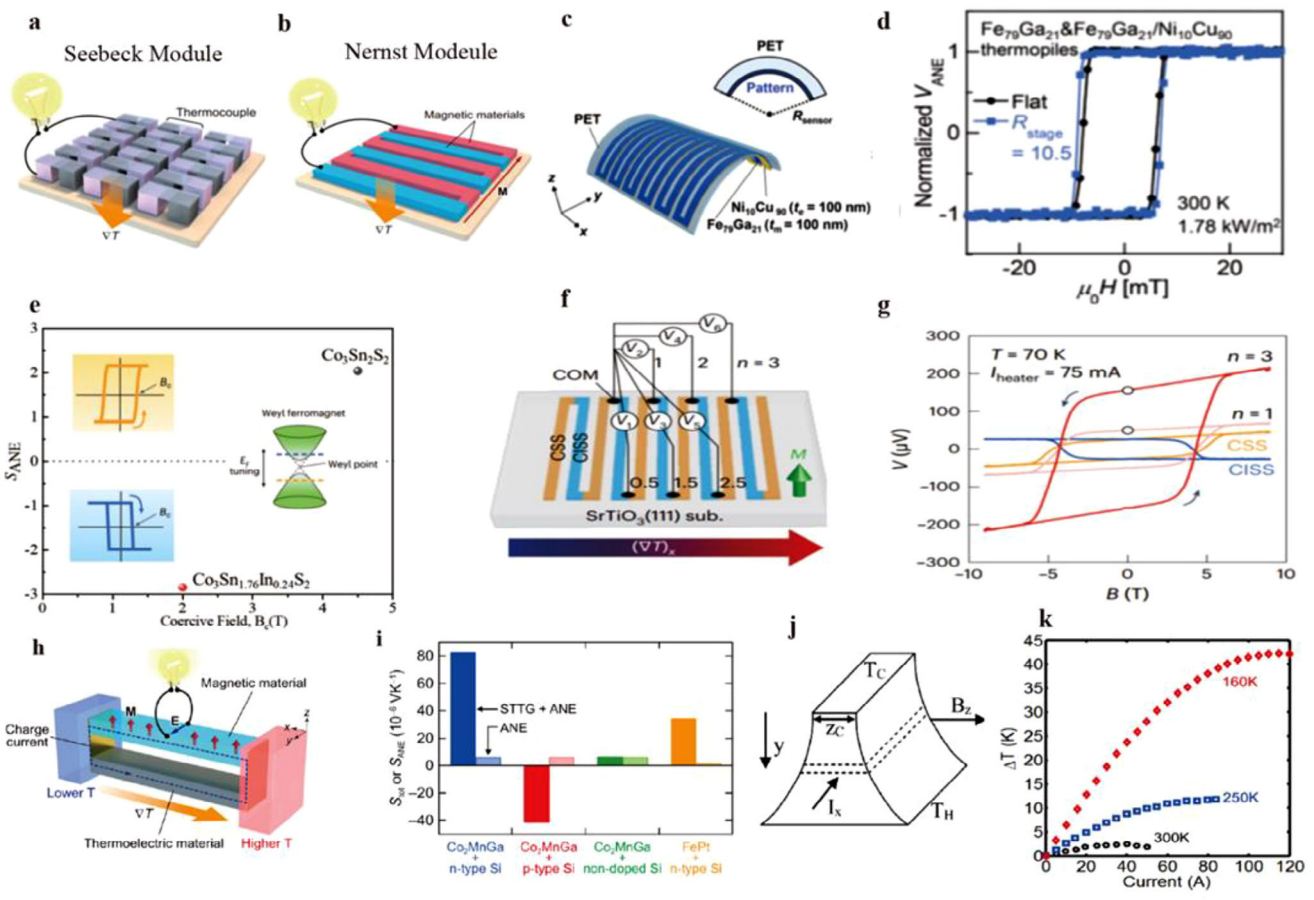
Thermoelectric generation and Ettingshausen cooler. a) Conventional Seebeck‐based module composed of two different material types.^[^ ^176^ ^]^ b) Nernst‐based thermopile with a planar structure, capable of being made from a single thermoelectric material. The polarity of the voltage can be reversed by altering the magnetization (*M*) direction. a,b) Adapted with permission.^[^ ^176^ ^]^ Copyright 2021, AIP Publishing. c) Schematic diagram of a flexible anomalous Nernst thermoelectric sensor structure, consisting of Fe‐Ga thin films (ANE materials) and Ni‐Cu (electrode materials) on PET substrates. d) ANE voltage as a function of magnetic field for the sensors on the bending stage (blue squares) and the flat stage (black circles) at 300K, demonstrating the flexibility of the sensor. c,d) Adapted with permission from^[^ ^207^ ^]^ under the Creative Commons CC BY license. e) ANE signal as a function of the coercive field for Co_3_Sn_2_S_2_ and Co_3_Sn_1.76_In_0.24_S_2_ with different ANE polarity. The polarity of ANE (positive indicated by yellow hysteresis loop and negative indicated by blue hysteresis loop) can be controlled by tuning the Fermi energy across the exchange gap of the Weyl band. f) Schematic illustration of a thermopile fabricated by Co_3_Sn_2_S_2_ (orange region, CSS) and Co_3_In_0.24_Sn_1.83_S_2_ (blue region, CISS) in series. g) ANE voltage along y direction as a function of magnetic field (z direction) when applying a heater current along x direction. e–g) Adapted with permission.^[^ ^208^ ^]^ Copyright 2024, Springer Nature. h) Schematic illustration of Seebeck‐driven transverse thermoelectric generation (STTG) in a closed circuit comprising conventional thermoelectric and magnetic materials electrically connected at both ends. The Seebeck effect in thermoelectric materials can generate a charge current, and then the charge current can be converted into a transverse electric field in the magnetic material via the anomalous Hall effect.^[^ ^176^ ^]^ i) Room temperature transverse thermal voltage values (*S*_tot_) for STTG samples with magnetic material ‒ conventional thermoelectric material connections, namely, Co_2_MnGa‐*n*‐type Si, Co_2_MnGa‐*p*‐type Si, Co_2_MnGa‐nondoped Si, and FePt‐*n*‐type Si, as well as the *S*_ANE_ of magnetic materials (Co_2_MnGa and FePt) without connections.h and i adapted with permission.^[^ ^185^ ^]^ Copyright 2021, AIP Publishing.^[^ ^176^ ^]^ j) Schematic diagram of an infinite staged Ettingshausen cooler with a cascade structure using a single piece of thermoelectric material. k) The temperature difference as a function of current at various heat sink temperatures of 160, 250, and 300K for an Ettingshausen device fabricated from Bi_0.97_Sb_0.03_ material. j,k) Adapted with permission.^[^ ^209^ ^]^ Copyright 2016, IOP Publishing.

With recent advancements in high magnetic field technologies and topological material science, Nernst‐based TE devices have garnered renewed interest.^[^ ^202^, ^203^, ^204^, ^205^ ^]^ The Nernst effect enables direct TE energy conversion in a transverse configuration, thereby offering numerous advantages over conventional longitudinal TE effects. Compared with longitudinal TE devices (**Figure** **24**a), the Nernst thermopile (Figure 24b) features a planar structure where magnetic wires are connected in series across a heat source. Importantly, this architecture does not require both n‐ and p‐type materials, as the voltage polarity can be reversed by alternating the magnetization orientation of adjacent elements. Furthermore, the output voltage of transverse TE devices can be amplified by increasing the length of magnetic wires attached to the heat source.^[^ ^176^ ^]^ Specially, the Nernst‐based thermopiles allow for the exploitation of diverse heat source shapes, making them particularly suitable for applications such as heat flux sensors with low thermal resistance and high flexibility. A recently proposed V‐shaped configuration utilizes topological antiferromagnet Mn₃Sn thin films, which produce a substantial voltage without any conductive wires. This is achieved through a large angle between the applied field and magnetization due to the disruption of magnetic octupoles at the planar edge of Mn₃Sn.^[^ ^206^ ^]^ This configuration offers several advantages, including contactless operation, a simple structure, and a tunable voltage sign by adjusting the tilting angle. Additionally, an ANE‐based heat flux sensor (Figure 24c) uses Fe‐Ga thin films as ANE materials and Ni‐Cu thin films as electrodes on polyethylene terephthalate (PET) substrate.^[^ ^207^ ^]^ These sensors can directly sense perpendicular heat flux, as the Seebeck coefficient of the Ni‐Cu alloy can be adjusted to match that of the Fe‐Ga alloy, thus eliminating the Seebeck contribution in the thermopile device. The normalized Nernst voltage of the sensors remains stable under bending and flat conditions at 300 K (Figure 24d).

By fine‐tuning the Fermi energy across the exchange gap of the Weyl band, the polarity of the ANE—and thus the direction of the transverse voltage hysteresis loop—can be controlled, as demonstrated by substituting Co with In in Co₃Sn₂S₂ thin films while preserving their topological band structure and large coercive field (Figure 24e).^[^ ^208^ ^]^ This device, composed of Co₃Sn₂S₂ (orange) and Co_3_In_0.24_Sn_1.83_S_2_ (blue) films (Figure 24f), generates a significant ANE voltage along the y‐axis when a temperature gradient is applied along the x‐axis. The voltage output scales with the number of pairs (Figure 24g), and the device can function at zero magnetic field after field‐cooling, with the magnetization of the films oriented out‐of‐plane.

Despite these innovations, the practical efficiency of ANE‐based devices is currently limited by the relatively small ANE thermopower compared to the Seebeck coefficient. It is worth mentioning that Zhou et al. recently proposed a novel concept known as Seebeck‐driven transverse thermoelectric generation (STTG).^[^ ^210^ ^]^ This STTG device consists of a conventional TE leg and a ferromagnetic leg connected in a closed circuit (Figure 24h). When a temperature gradient is applied, the conventional TE material drives a charge current through the magnetic material, which is then converted into a transverse electric field due to the anomalous Hall effect (AHE) in the magnetic material. As a result, this configuration increases the magnitude of the transverse voltage, leading to a signal as large as 82.3 (−41.0) µV K^−1^ in Co_2_MnGa/*n*(*p*)‐type‐Si hybrid material (Figure 24i), which is one order of magnitude larger than the record‐high *S*_ANE_.

In summary, the three‐dimensionality of the ANE can greatly simplify thermopile structures^[^ ^204^ ^]^ and enable versatile, low‐cost energy harvester for Internet‐of‐Things (IoT) sensors and wearable devices. Notably, certain Weyl semimetals, such as NbP, exhibit both large longitudinal magneto‐Seebeck and transverse Nernst effects, presenting further potential for combined TE applications.

### 3.2 Cryogenic Cooling Applications

TE materials that work efficiently at very low temperatures have promising applications in the field of solid‐state cryogenic cooling. An ideal scheme for cryogenic solid‐state cooling is to use a Peltier cooler. Strongly correlated electron systems, such as Kondo insulators^[^ ^211^, ^212^ ^]^ and heavy fermion system,^[^ ^213^, ^214^, ^215^ ^]^ are suggested as potential candidates for low‐temperature TE cooling applications. Topological materials demonstrate a giant magneto‐Peltier effect at cryogenic temperatures when they are subjected in a magnetic field, which may provide a good approach to bring TE cryogenic cooling closer to reality. It has been reported that *n*‐type topological insulator BiSb alloys have high *zT* values (*zT* > 1) in the temperature range of 120 K–270 K when a magnetic field is applied.^[^ ^216^ ^]^ Unfortunately, there is a lack of efficient *p*‐type low temperature TE material with a matching *zT* to make an efficient cryocooler. Furthermore, multistage Peltier coolers need to be built to reach cryogenic temperatures because the maximum temperature difference is limited to $\Delta \;T_{MAX}=\frac{1}{2}\;zT_{C}^{2}$ (*T_c_*: cold side temperature).^[^ ^217^ ^]^

The transverse Nernst–Ettingshausen effect is more favorable for cooling application below room temperature compared to the Peltier effect, because of its superiority in material optimization and device configuration. Peltier coolers need a matrix of *n*‐ and *p*‐type TE legs electrically connected in series and thermally in parallel to achieve substantial cooling power, while Nernst‐Ettingshausen coolers require only one type of material, and even one single bar of materials can achieve the required cooling power. For substantial cooling power, one can design a cascade structure with a small cross‐sectional area in the direction of the electrical current and a large cross‐ sectional area in the direction of the heat flow. An infinite‐stage‐cascade Ettingshausen cooler with an exponential shape that was fabricated from Bi_0.97_Sb_0.03_ has shown Δ*T*≈42K (Figure 24j,k), starting from 160K, at 0.75 T,^[^ ^209^, ^218^ ^]^ indicating that all solid‐state cooling to 118K is possible. TSMs with a large Nernst thermopower and power factor are potential candidates for solid‐state Nernst–Ettingshausen coolers.

## 4 Outlook

The integration of topological science into TE research has given rise to an exciting and rapidly developing field known as topological thermoelectrics. This interdisciplinary area offers profound opportunities and challenges, both for advancing fundamental physics and for developing high‐performance energy conversion materials. The rich physics of topological phases, such as their unique nontrivial band structures and quantum surface states, holds immense potential for designing TE materials and devices with improved efficiency and novel functionalities, including those enabled by transverse thermoelectric devices. Recent studies have demonstrated high‐efficiency TE effects, such as the Seebeck effect, magneto‐Seebeck effect, Nernst effect, and anomalous Nernst effect, in various topological materials. These effects are closely linked to nontrivial electronic features, such as inverted bulk bands, topological surface states, linear Dirac/Weyl dispersions, and large Berry curvatures. This review has summarized key theoretical and experimental advances in understanding and manipulating TE transport phenomena in TMs, highlighting the roles of different topological states in achieving better TE performance.

Despite the promise of topological thermoelectrics, several challenges remain in advancing this field, particularly in fully understanding the role of topology in TE transport phenomena. While bulk topological insulators (TIs) exhibit promising TE properties through conventional optimization strategies, experimentally verifying the significant enhancement of TE efficiency via topological surface states remains elusive. Theoretical predictions suggest high *zT* values in 2D TIs, but challenges such as separating surface‐state contributions from bulk states, and addressing the effects of temperature, chemical potential, impurities, and defects, continue to impede progress. To advance experimental research, it is essential to develop ideal TI systems characterized by minimal defects, highly insulating bulk states, and high‐quality nanostructures. Additionally, the development of advanced experimental techniques capable of high‐precision characterization of topological band structures and TE transport properties is crucial. The competition between topological and bulk states further emphasizes the need to precisely tune parameters such as material dimensions, defect levels, and Fermi energy to optimize TE performance. The role of topological boundary states in bulk TI composites and heterostructures also remains poorly understood and represents an exciting avenue for enhancing *zT*. Beyond surface state contributions in TIs, recent studies have underscored the critical role of bulk transport phenomena—particularly those driven by band inversion—in shaping TE properties. Bulk band inversion is especially relevant for TE transport in the bulk, yet research on bulk states and their charge transport characteristics in TIs remains limited. Expanding the scope of bulk TE studies could unlock new design strategies for developing advanced TE materials by harnessing band inversion‐driven effects. Topological phase transitions, induced by high pressure or chemical doping, have been identified as effective strategies for achieving high *zT* values. However, there is considerable interest in exploring alternative methods to induce topological phases and further enhance TE performance beyond conventional strategies. Nevertheless, the intersection of TIs and TEs presents promising opportunities for discovering new high‐performance TE materials.

TSMs are generally considered as poor TE materials due to the presence of bipolar effects. However, some uncompensated semimetals, characterized by a combination of linear dispersion and conventional bands near the Fermi level, exhibit band asymmetry that enhances thermopower and carrier mobility while minimizing bipolar effects. Most of these semimetals can demonstrate large Peltier conductivity and power factor at cryogenic temperature, enabling applications like cryogenic cooling that are beyond the reach of conventional semiconductors. Therefore, the potential of TSMs with complex bands deserves further exploration. Under perpendicular magnetic fields, TSMs show very large longitudinal and transverse magneto‐thermopowers due to their gapless linear band dispersions. These can lead to very large magneto‐thermoelectric power factors or figures of merit below room temperature, which are promising for cryogenic cooling applications. However, the relatively high phonon thermal conductivity and low average *zT* values of current materials, coupled with the requirement for large magnetic fields, limit their practical use. As such, future research should focus on identifying novel TSMs that combine high carrier mobility and low phonon group velocities, thereby improving the magneto‐thermoelectric performance. Additionally, there is a growing need for materials that exhibit large thermopower in moderate magnetic fields, thus overcoming the challenges of magnetic field reliance. Topological magnets, which exhibit significant transverse anomalous Nernst effects (ANE) without external magnetic fields, hold great promise for the development of efficient, flexible, and densely integrated transverse thermoelectric devices. However, current ANE values remain below the thresholds required for practical applications, necessitating further advancements in materials design. Beyond intrinsic mechanisms, exploring novel physical phenomena, such as spin‐dependent impurity scattering and magnon‐electron coupling, may significantly improve performance. Another critical gap in current research is the exploration of materials that can operate effectively above room temperature, especially for power generation applications. Magnetic materials with high Curie temperatures hold particular promise due to their ability to maintain robust thermoelectric properties at elevated temperatures. While traditional TE devices have predominantly focused on longitudinal effects, transverse TE devices and hybrid TE device combining longitudinal and transverse TE effects, remains relatively underexplored. These new TE devices may offer potential application in electronics, sensors, and beyond.

An additional unexplored frontier lies in the influence of topological phonons on TE performance. While topological phonons are theorized to impact thermal transport significantly, their role in TE materials remains largely unknown. Understanding how topological phonons interact with electronic and lattice properties could provide groundbreaking insights for reducing lattice thermal conductivity and enhancing overall TE efficiency.

In summary, the emergence of topological science offers innovative concepts and mechanisms for designing the next‐generation highly efficiency energy conversion materials and devices. The diverse and rich topological band structures provide unprecedented opportunities to manipulate TE transport properties, through dimensional tuning, symmetry manipulation, topological phase transitions, and external field modulation. As research on topological TE advances, it is expected that new types of topological materials will be discovered, with more comprehensive theoretical analyses and experimental validations leading to greater progress and breakthroughs in the field. These innovations promise to offer novel solutions for energy conversion and utilization, potentially reshaping the landscape of thermoelectric technology in the near future.

## Conflict of Interest

The authors declare no conflict of interest.

## References

[1] L. E. C. Bell , Science 2008, 321, 1457. 18787160 10.1126/science.1158899

[2] S. M. Pourkiaei , M. H. Ahmadi , M. Sadeghzadeh , S. Moosavi , F. Pourfayaz , L. Chen , M. A. Pour Yazdi , R. Kumar , Energy 2019, 186, 115849.

[3] D. Champier , Energy Convers. Manage. 2017, 140, 167.

[4] L. Yang , Z. G. Chen , M. S. Dargusch , J. Zou , Adv. Energy Mater. 2018, 8, 1701797.

[5] F. J. DiSalvo , Science 1999, 285, 703. 10426986 10.1126/science.285.5428.703

[6] G. Tan , F. Shi , S. Hao , H. Chi , L.‐D. Zhao , C. Uher , C. Wolverton , V. P. Dravid , M. G. Kanatzidis , J. Am. Chem. Soc. 2015, 137, 5100. 25856499 10.1021/jacs.5b00837

[7] Y. Pei , H. Wang , G. J. Snyder , Adv. Mater. 2012, 24, 6125. 23074043 10.1002/adma.201202919

[8] Y. Tang , Z. M. Gibbs , L. A. Agapito , G. Li , H.‐S. Kim , M. B. Nardelli , S. Curtarolo , G. J. Snyder , Nat. Mater. 2015, 14, 1223. 26436339 10.1038/nmat4430

[9] J. Xin , Y. Tang , Y. Liu , X. Zhao , H. Pan , T. Zhu , npj Quantum Mater. 2018, 3, 9.

[10] J. P. Heremans , B. Wiendlocha , A. M. Chamoire , Energy Environ. Sci. 2012, 5, 5510.

[11] X. Shi , L. Chen , C. Uher , Int. Mater. Rev. 2016, 61, 379.

[12] J.‐F. Li , W.‐S. Liu , L.‐D. Zhao , M. Zhou , NPG Asia Mater. 2010, 2, 152.

[13] C. J. Vineis , A. Shakouri , A. Majumdar , M. G. Kanatzidis , Adv. Mater. 2010, 22, 3970. 20661949 10.1002/adma.201000839

[14] M. S. Dresselhaus , G. Chen , M. Y. Tang , R. G. Yang , H. Lee , D. Z. Wang , Z. F. Ren , J.‐P. Fleurial , P. Gogna , Adv. Mater. 2007, 19, 1043.

[15] Y. Zhou , L. D. Zhao , Adv. Mater. 2017, 29, 1702676.

[16] W. Kim , J. Mater. Chem. C 2015, 3, 10336.

[17] Z. Chen , X. Zhang , Y. Pei , Adv. Mater. 2018, 30, 1705617. 10.1002/adma.201705617 29399915

[18] R. Fortulan , S. Aminorroaya Yamini , Materials (Basel) 2021, 14, 6059. 34683651 10.3390/ma14206059 PMC8540781

[19] M. J. Gilbert , Commun. Phys. 2021, 4, 70.

[20] Y. Liu , X. Chen , Y. Xu , Adv. Funct. Mater. 2019, 30, 1904784. PMC7537547 33029110

[21] A. Bansil , H. Lin , T. C. Das , Rev. Mod. Phys. 2016, 88, 021004.

[22] Q. L. He , T. L. Hughes , N. P. Armitage , Y. Tokura , K. L. Wang , Nat. Mater. 2022, 21, 15. 34949869 10.1038/s41563-021-01138-5

[23] H. Luo , P. Yu , G. Li , K. Yan , Nat. Rev. Phys. 2022, 4, 611.

[24] C. Bao , P. Tang , D. Sun , S. Zhou , Nat. Rev. Phys. 2022, 4, 33.

[25] J. P. Heremans , R. J. Cava , N. Samarth , Nat. Rev. Mater. 2017, 2, 17049.

[26] H. Zhang , C.‐X. Liu , X.‐L. Qi , X. Dai , Z. Fang , S.‐C. Zhang , Nat. Phys. 2009, 5, 438.

[27] Y. M. Zuev , J. S. Lee , C. Galloy , H. Park , P. Kim , Nano Lett. 2010, 10, 3037. 20698617 10.1021/nl101505q

[28] A. Mavrokefalos , A. L. Moore , M. T. Pettes , L. Shi , W. Wang , X. Li , J. Appl. Phys. 2009, 105, 104318.

[29] J. Zhang , X. Feng , Y. Xu , M. Guo , Z. Zhang , Y. Ou , Y. Feng , K. Li , H. Zhang , L. Wang , X. Chen , Z. Gan , S.‐C. Zhang , K. He , X. Ma , Q.‐K. Xue , Y. Wang , Phys. Rev. B 2015, 91, 075431.

[30] Y. Xu , Z. Gan , S.‐C. Zhang , Phys. Rev. Lett. 2014, 112, 226801. 24949782 10.1103/PhysRevLett.112.226801

[31] P. K. Ghose , T. K. Dalui , A. Chatterjee , S. Majumdar , S. Giri , Appl. Phys. Lett. 2021, 118, 243901.

[32] W. Zhang , P. Wang , B. Skinner , R. Bi , V. Kozii , C. W. Cho , R. Zhong , J. Schneeloch , D. Yu , G. Gu , L. Fu , X. Wu , L. Zhang , Nat. Commun. 2020, 11, 1046. 32098952 10.1038/s41467-020-14819-7 PMC7042294

[33] H. Yang , W. You , J. Wang , J. Huang , C. Xi , X. Xu , C. Cao , M. Tian , Z.‐A. Xu , J. Dai , Y. Li , Phys. Rev. Mater. 2020, 4, 024202.

[34] C. Fu , S. N. Guin , S. J. Watzman , G. Li , E. Liu , N. Kumar , V. Süβ , W. Schnelle , G. Auffermann , C. Shekhar , Y. Sun , J. Gooth , C. Felser , Energy Environ. Sci. 2018, 11, 2813.

[35] N. Xu , Y. Xu , J. Zhu , npj Quantum Mater. 2017, 2, 1.

[36] C. Fu , Y. Sun , C. T. Felser , APL Mater. 2020, 8, 040913.

[37] T. Yang , Y. Yang , X. Wang , G. Zhang , Z. Cheng , Mater. Today Chem. 2023, 30, 101488.

[38] P. Narang , C. A. C. Garcia , C. Felser , Nat. Mater. 2021, 20, 293. 33139890 10.1038/s41563-020-00820-4

[39] B. Singh , H. Lin , A. Bansil , Adv. Mater. 2023, 35, 2201058. 10.1002/adma.202201058 36414399

[40] B. Yan , C. Felser , Annu. Rev. Condens. Matter Phys. 2017, 8, 337.

[41] F. Zhang , C. L. Kane , E. J. Mele , Phys. Rev. B 2012, 86, 081303.

[42] T. Zhang , P. Cheng , X. Chen , J.‐F. Jia , X. Ma , K. He , L. Wang , H. Zhang , X. Dai , Z. Fang , X. Xie , Q.‐K. Xue , Phys. Rev. Lett. 2009, 103, 266803. 20366330 10.1103/PhysRevLett.103.266803

[43] J. C. Teo , L. Fu , C. Kane , Phys. Rev. B—Condens. Matter. Mater. Phys. 2008, 78, 045426.

[44] J. Ma , C. Yi , B. Lv , Z. Wang , S. Nie , L. Wang , L. Kong , Y. Huang , P. Richard , P. Zhang , K. Yaji , K. Kuroda , S. Shin , H. Weng , B. A. Bernevig , Y. Shi , T. Qian , H. Ding , Sci. Adv. 2017, 3, 1602415. 10.1126/sciadv.1602415 PMC5419706 28508059

[45] C.‐C. Liu , J.‐J. Zhou , Y. Yao , F. Zhang , Phys. Rev. Lett. 2016, 116, 066801. 26919004 10.1103/PhysRevLett.116.066801

[46] R. Noguchi , T. Takahashi , K. Kuroda , M. Ochi , T. Shirasawa , M. Sakano , C. Bareille , M. Nakayama , M. D. Watson , K. Yaji , A. Harasawa , H. Iwasawa , P. Dudin , T. K. Kim , M. Hoesch , V. Kandyba , A. Giampietri , A. Barinov , S. Shin , R. Arita , T. Sasagawa , T. Kondo , Nature 2019, 566, 518. 30742073 10.1038/s41586-019-0927-7

[47] R. Noguchi , M. Kobayashi , K. Kawaguchi , W. Yamamori , K. Aido , C. Lin , H. Tanaka , K. Kuroda , A. Harasawa , V. Kandyba , M. Cattelan , A. Barinov , M. Hashimoto , D. Lu , M. Ochi , T. Sasagawa , T. Kondo , Phys. Rev. Lett. 2024, 133, 086602. 39241706 10.1103/PhysRevLett.133.086602

[48] Y. Tanaka , Z. Ren , T. Sato , K. Nakayama , S. Souma , T. Takahashi , K. Segawa , Y. Ando , Nat. Phys. 2012, 8, 800.

[49] S.‐Y. Xu , C. Liu , N. Alidoust , M. Neupane , D. Qian , I. Belopolski , J. D. Denlinger , Y. J. Wang , H. Lin , L. A. Wray , G. Landolt , B. Slomski , J. H. Dil , A. Marcinkova , E. Morosan , Q. Gibson , R. Sankar , F. C. Chou , R. J. Cava , A. Bansil , M. Z. Hasan , Nat. Commun. 2012, 3, 1192. 23149737 10.1038/ncomms2191

[50] P. Dziawa , B. J. Kowalski , K. Dybko , R. Buczko , A. Szczerbakow , M. Szot , E. Lusakowska , T. Balasubramanian , B. M. Wojek , M. H. Berntsen , O. Tjernberg , T. Story , Nat. Mater. 2012, 11, 1023. 23023551 10.1038/nmat3449

[51] S. Y. Matsushita , K. Ichimura , K. K. Huynh , K. Tanigaki , Phys. Rev. Mater. 2021, 5, 014205.

[52] S. Wang , B.‐C. Lin , A.‐Q. Wang , D.‐P. Yu , Z.‐M. Liao , Adv. Phys.: X 2017, 2, 518.

[53] N. Kumar , S. N. Guin , K. Manna , C. Shekhar , C. Felser , Chem. Rev. 2021, 121, 2780. 33151662 10.1021/acs.chemrev.0c00732 PMC7953380

[54] Z. K. Ding , Y. J. Zeng , W. Liu , L. M. Tang , K. Q. Chen , Adv. Funct. Mater. 2024, 34, 2401684.

[55] T. Zhang , Z. Song , A. Alexandradinata , H. Weng , C. Fang , L. Lu , Z. Fang , Phys. Rev. Lett. 2018, 120, 016401. 29350958 10.1103/PhysRevLett.120.016401

[56] J. Li , Q. Xie , S. Ullah , R. Li , H. Ma , D. Li , Y. Li , X.‐Q. Chen , Phys. Rev. B 2018, 97, 054305.

[57] G. Liu , Y. Jin , Z. Chen , H. Xu , Phys. Rev. B 2021, 104, 024304.

[58] Q.‐B. Liu , Z.‐Q. Wang , H.‐H. Fu , Phys. Rev. B 2021, 104, L041405.

[59] C. Xie , H. Yuan , Y. Liu , X. Wang , Phys. Rev. B 2022, 105, 054307.

[60] Z.‐K. Ding , Y.‐J. Zeng , H. Pan , N. Luo , J. Zeng , L.‐M. Tang , K.‐Q. Chen , Phys. Rev. B 2022, 106, L121401.

[61] W. Liu , Z.‐K. Ding , N. Luo , J. Zeng , L.‐M. Tang , K.‐Q. Chen , Phys. Rev. B 2024, 109, 115422.

[62] J. Zhu , W. Wu , J. Zhao , C. Chen , Q. Wang , X.‐L. Sheng , L. Zhang , Y. X. Zhao , S. A. Yang , Phys. Rev. B 2022, 105, 085123.

[63] L. Zhang , J. Ren , J.‐S. Wang , B. Li , Phys. Rev. Lett. 2010, 105, 225901. 21231397 10.1103/PhysRevLett.105.225901

[64] S. Singh , Q. Wu , C. Yue , A. H. Romero , A. A. Soluyanov , Phys. Rev. Mater. 2018, 2, 114204.

[65] Z.‐Y. Ong , C. H. Lee , Phys. Rev. B 2016, 94, 134203.

[66] Z.‐K. Ding , Y.‐J. Zeng , H. Pan , N. Luo , L.‐M. Tang , J. Zeng , K.‐Q. Chen , Phys. Rev. B 2024, 109, 245104.

[67] G. W. Winkler , S. Singh , A. A. Soluyanov , Chin. Phys. B 2019, 28, 077303.

[68] V. K. Sharma , V. Kanchana , M. K. Gupta , R. Mittal , Mater. Today Commun. 2023, 35, 106289.

[69] X. Jin , D.‐s. Ma , R. Wang , X. Lv , X. Yang , Cell Reports Physical Science 5 2023, 4.

[70] R. Schaffer , E. K.‐H. Lee , B.‐J. Yang , Y. B. Kim , Rep. Prog. Phys. 2016, 79, 094504. 27540689 10.1088/0034-4885/79/9/094504

[71] Z.‐Y. Ye , H.‐X. Deng , H.‐Z. Wu , S.‐S. Li , S.‐H. Wei , J.‐W. Luo , npj Comput. Mater. 2015, 1, 1.

[72] Z. Zhu , Y. Cheng , U. Schwingenschlögl , Phys. Rev. B 2012, 85, 235401. 10.1103/PhysRevLett.108.266805 23005005

[73] R. Pathak , P. Dutta , A. Srivastava , D. Rawat , R. K. Gopal , A. K. Singh , A. Soni , K. Biswas , Angew. Chem. 2022, 134, 202210783. 10.1002/anie.202210783 35971950

[74] M. Samanta , K. Pal , P. Pal , U. V. Waghmare , K. Biswas , J. Am. Chem. Soc. 2018, 140, 5866. 29641193 10.1021/jacs.8b02691

[75] M. Samanta , K. Pal , U. V. Waghmare , K. Biswas , Angew. Chem., Int. Ed. 2020, 59, 4822. 10.1002/anie.202000343 31970889

[76] N. Wang , M. Li , H. Xiao , Z. Gao , Z. Liu , X. Zu , S. Li , L. Qiao , npj Comput. Mater. 2021, 7, 18.

[77] J. P. Heremans , R. J. Cava , N. Samarth , Nat. Rev. Mater. 2017, 2, 17049.

[78] M. Y. Toriyama , G. J. Snyder , Mater. Horiz. 2024, 11, 1188. 38189468 10.1039/d3mh01930f

[79] I. T. Witting , F. Ricci , T. C. Chasapis , G. Hautier , G. J. Snyder , Research (Wash D C) 2020, 2020, 4361703. 32285043 10.34133/2020/4361703 PMC7150672

[80] M. Y. Toriyama , G. J. Snyder , The Innovation 2025, 10.1016/j.xinn.2024.100782. PMC11910883 40098672

[81] M. Y. Toriyama , G. J. Snyder , Cell Rep. Phys. Sci. 2023, 4, 101392.

[82] D. A. Polvani , J. F. Meng , N. V. Chandra Shekar , J. Sharp , J. V. Badding , Chem. Mater. 2001, 13, 2068.

[83] F.‐X. Bai , Adv. Sci. 2022, 9, 2105709.

[84] H.‐X. Wang , L.‐S. Mao , X. Tan , G.‐Q. Liu , J. Xu , H. Shao , H. Hu , J. Jiang , Nano Energy 2018, 51, 649.

[85] I. V. Korobeinikov , N. V. Morozova , L. N. Lukyanova , O. A. Usov , S. V. Ovsyannikov , Semiconductors 2019, 53, 732.

[86] A. Kore , H. Murari , P. Singh , J. Phys. D: Appl. Phys. 2021, 54, 305503.

[87] L.‐C. Chen , P.‐Q. Chen , W.‐J. Li , Q. Zhang , V. V. Struzhkin , A. F. Goncharov , Z. Ren , X.‐J. Chen , Nat. Mater. 2019, 18, 1321. 31591530 10.1038/s41563-019-0499-9

[88] S. Xie , X. Wan , Y. Wu , C. Li , F. Yan , Y. Ouyang , H. Ge , X. Li , Y. Liu , R. Wang , M. Y. Toriyama , G. J. Snyder , J. Yang , Q. Zhang , W. Liu , X. Tang , Adv. Mater. 2024, 36, 2400845. 10.1002/adma.202400845 38651256

[89] Y. Ando , J. Phys. Soc. Jpn. 2013, 82, 102001.

[90] Y. L. Chen , J. G. Analytis , J.‐H. Chu , Z. K. Liu , S.‐K. Mo , X. L. Qi , H. J. Zhang , D. H. Lu , X. Dai , Z. Fang , S. C. Zhang , I. R. Fisher , Z. Hussain , Z.‐X. Shen , Experimental Realization of a Three‐Dimensional Topological Insulator, Bi 2 Te 3. Science 2009, 325, 178. 10.1126/science.1173034 19520912

[91] S. Ding , X. Chen , Y. Xu , W. Duan , npj Comput. Mater. 2023, 9, 189.

[92] H. S. Shin , B. Hamdou , H. Reith , H. Osterhage , J. Gooth , C. Damm , B. Rellinghaus , E. Pippel , K. Nielsch , Nanoscale 2016, 8, 13552. 27362294 10.1039/c6nr01716a

[93] M. Cassinelli , S. Müller , K.‐O. Voss , C. Trautmann , F. Völklein , J. Gooth , K. Nielsch , M. E. Toimil‐Molares , Nanoscale 2017, 9, 3169. 28221383 10.1039/c6nr09624g

[94] H. Osterhage , J. Gooth , B. Hamdou , P. Gwozdz , R. Zierold , K. Nielsch , Appl. Phys. Lett. 2014, 105, 123117.

[95] S. Souma , M. Komatsu , M. Nomura , T. Sato , A. Takayama , T. Takahashi , K. Eto , K. Segawa , Y. Ando , Phys. Rev. Lett. 2012, 109, 186804. 23215312 10.1103/PhysRevLett.109.186804

[96] P. Ghaemi , R. S. Mong , J. E. Moore , Phys. Rev. Lett. 2010, 105, 166603. 21230991 10.1103/PhysRevLett.105.166603

[97] H. Osterhage , J. Gooth , B. Hamdou , P. Gwozdz , R. Zierold , K. Nielsch , Appl. Phys. Lett. 2014, 105, 123117.

[98] S. Y. Matsushita , K. K. Huynh , H. Yoshino , N. H. Tu , Y. Tanabe , K. Tanigaki , Phys. Rev. Mater. 2017, 1, 054202.

[99] M. Yarmohammadi , K. Mirabbaszadeh , J. Mater. Chem. A 2019, 7, 25573.

[100] M. Paulsson , S. Datta , Phys. Rev. B 2003, 67, 241403.

[101] Y. Xu , Chin. Phys. B 2016, 25, 117309.

[102] B. Hamdou , J. Gooth , T. Böhnert , A. Dorn , L. Akinsinde , E. Pippel , R. Zierold , K. Nielsch , Adv. Energy Mater. 2015, 5, 1500280.

[103] J. Gooth , J. G. Gluschke , R. Zierold , M. Leijnse , H. Linke , K. Nielsch , Semicond. Sci. Technol. 2015, 30, 015015.

[104] H. Hamasaki , Y. Tokumoto , K. Edagawa , J. Phys. Soc. Jpn. 2020, 89, 023703.

[105] M. Tahir , A. Manchon , U. Schwingenschlögl , J. Appl. Phys. 2014, 116, 093708.

[106] L.‐L. Li , W. Xu , Chin. Phys. Lett. 2015, 32, 047304.

[107] P. H. Chang , M. S. Bahramy , N. Nagaosa , B. K. Nikolic , Nano Lett. 2014, 14, 3779. 24932511 10.1021/nl500755m

[108] J. Liang , L. Cheng , J. Zhang , H. Liu , Z. Zhang , Nanoscale 2016, 8, 8855. 27071548 10.1039/c6nr00724d

[109] B. Poudel , Q. Hao , Y. Ma , Y. Lan , A. Minnich , B. Yu , X. Yan , D. Wang , A. Muto , D. Vashaee , X. Chen , J. Liu , M. S. Dresselhaus , G. Chen , Z. Ren , Science 2008, 320, 634. 18356488 10.1126/science.1156446

[110] D. Kim , P. Syers , N. P. Butch , J. Paglione , M. S. Fuhrer , Nano Lett. 2014, 14, 1701. 24605897 10.1021/nl4032154

[111] N.‐X. Yang , Y.‐F. Zhou , P. Lv , Q.‐F. Sun , Phys. Rev. B 2018, 97, 235435.

[112] M. Markov , S. E. Rezaei , S. N. Sadeghi , K. Esfarjani , M. Zebarjadi , Thermoelectric properties of semimetals. Physical Review Materials, 2019, 10.1103/physrevmaterials.3.095401.

[113] S. Han , Z. Zhou , C. Sheng , R. Hu , H. Yuan , Q. Tang , H. Liu , Mater. Today Phys. 2021, 21, 100560.

[114] J. Mao , H. Zhu , Z. Ding , Z. Liu , G. A. Gamage , G. Chen , Z. Ren , Science 2019, 365, 495. 31320557 10.1126/science.aax7792

[115] M. Kim , S. i. Kim , S. W. Kim , H. S. Kim , K. H. Lee , Adv. Mater. 2021, 33, 2170371.

[116] G. J. Snyder , A. H. Snyder , M. Wood , R. Gurunathan , B. H. Snyder , C. Niu , Adv. Mater. 2020, 32, 2001537. 10.1002/adma.202001537 32410214

[117] M. Markov , S. E. Rezaei , S. N. Sadeghi , K. Esfarjani , M. Zebarjadi , Phys. Rev. Mater. 2019, 3, 095401.

[118] Y. Pan , F.‐R. Fan , X. Hong , B. He , C. Le , W. Schnelle , Y. He , K. Imasato , H. Borrmann , C. Hess , B. Büchner , Y. Sun , C. Fu , G. J. Snyder , C. Felser , Adv. Mater. 2021, 33, 2003168. 33296128 10.1002/adma.202003168 PMC12121705

[119] Y. Okamoto , T. Wada , Y. Yamakawa , T. Inohara , K. Takenaka , Appl. Phys. Lett. 2018, 112, 173905.

[120] T. Inohara , Y. Okamoto , Y. Yamakawa , A. Yamakage , K. Takenaka , Appl. Phys. Lett. 2017, 110, 183901.

[121] H. Yao , C. Chen , W. Xue , F. Bai , F. Cao , Y. Lan , X. Liu , Y. Wang , D. J. Singh , X. Lin , Q. Zhang , Sci. Adv. 2021, 7, 6. 10.1126/sciadv.abd6162 PMC7864570 33547075

[122] C. Chen , W. Xue , S. Li , Z. Zhang , X. Li , X. Wang , Y. Liu , J. Sui , X. Liu , F. Cao , Z. Ren , C.‐W. Chu , Y. Wang , Q. Zhang , Proc. Natl. Acad. Sci. USA 2019, 116, 2831. 30718395 10.1073/pnas.1819157116 PMC6386660

[123] X. Feng , B. Skinner , Phys. Rev. Mater. 2021, 5, 024202.

[124] B. Skinner , L. L. Fu , Sci. Adv. 2018, 4, aat2621. 10.1126/sciadv.aat2621 PMC5969823 29806031

[125] V. Kozii , B. Skinner , L. Fu , Phys. Rev. B 2019, 99, 155123.

[126] H. Takahashi , R. Okazaki , S. Ishiwata , H. Taniguchi , A. Okutani , M. Hagiwara , I. Terasaki , Nat. Commun. 2016, 7, 12732. 27597055 10.1038/ncomms12732 PMC5025859

[127] M. Wu , N. Horing , H. Cui , Phys. Rev. B 1996, 54, 5438. 10.1103/physrevb.54.5438 9986502

[128] C. Li , N. H. Protik , N. K. Ravichandran , D. Broido , Phys. Rev. B 2023, 107, L081202.

[129] R. Fletcher , J. C. Maan , K. Ploog , G. Weimann , Phys. Rev. B 1986, 33, 7122. 10.1103/physrevb.33.7122 9938041

[130] M. Tsaousidou , P. N. Butcher , S. S. Kubakaddi , Phys. Rev. Lett. 1999, 83, 4820.

[131] T. M. Fromhold , P. N. Butcher , G. Qin , B. G. Mulimani , J. P. Oxley , B. L. Gallagher , Phys. Rev. B 1993, 48, 5326. 10.1103/physrevb.48.5326 10009051

[132] X. Xu , Y. Liu , G. Seyfarth , A. Pourret , W. Ma , H. Zhou , G. Wang , Z. Qu , S. Jia , Phys. Rev. B 2021, 104, 115164.

[133] Y. Fujishiro , N. Kanazawa , T. Shimojima , A. Nakamura , K. Ishizaka , T. Koretsune , R. Arita , A. Miyake , H. Mitamura , K. Akiba , M. Tokunaga , J. Shiogai , S. Kimura , S. Awaji , A. Tsukazaki , A. Kikkawa , Y. Taguchi , Y. Tokura , Nat. Commun. 2018, 9, 408. 29379016 10.1038/s41467-018-02857-1 PMC5789084

[134] Z. Fang , N. Nagaosa , K. S. Takahashi , A. Asamitsu , R. Mathieu , T. Ogasawara , H. Yamada , M. Kawasaki , Y. Tokura , K. Terakura , Science 2003, 302, 92. 14526076 10.1126/science.1089408

[135] D. Hsieh , Y. Xia , L. Wray , D. Qian , A. Pal , J. H. Dil , J. Osterwalder , F. Meier , G. Bihlmayer , C. L. Kane , Y. S. Hor , R. J. Cava , M. Z. Hasan , Science 2009, 323, 919. 19213915 10.1126/science.1167733

[136] Y. Pan , B. He , X. Feng , F. Li , D. Chen , U. Burkhardt , C. Felser , Nat. Mater. 2025, 24, 76. 39753855 10.1038/s41563-024-02059-9 PMC11698688

[137] T. Liang , Q. Gibson , J. Xiong , M. Hirschberger , S. P. Koduvayur , R. J. Cava , N. P. Ong , Nat. Commun. 2013, 4, 2696. 24176908 10.1038/ncomms3696

[138] J. L. Zhang , C. M. Wang , C. Y. Guo , X. D. Zhu , Y. Zhang , J. Y. Yang , Y. Q. Wang , Z. Qu , L. Pi , H.‐Z. Lu , M. L. Tian , Phys. Rev. Lett. 2019, 123, 196602. 31765179 10.1103/PhysRevLett.123.196602

[139] F. Han , N. Andrejevic , T. Nguyen , V. Kozii , Q. T. Nguyen , T. Hogan , Z. Ding , R. Pablo‐Pedro , S. Parjan , B. Skinner , A. Alatas , E. Alp , S. Chi , J. Fernandez‐Baca , S. Huang , L. Fu , M. Li , Nat. Commun. 2020, 11, 6167. 33268778 10.1038/s41467-020-19850-2 PMC7710760

[140] H. Wang , X. Luo , K. Peng , Z. Sun , M. Shi , D. Ma , N. Wang , T. Wu , J. Ying , Z. Wang , X. Chen , Adv. Funct. Mater. 2019, 29, 1902437.

[141] H. Wang , X. Luo , W. Chen , N. Wang , B. Lei , F. Meng , C. Shang , L. Ma , T. Wu , X. Dai , Z. Wang , X. Chen , Sci. Bull. 2018, 63, 411. 10.1016/j.scib.2018.03.010 36658935

[142] M. Matusiak , J. R. Cooper , D. Kaczorowski , Nat. Commun. 2017, 8, 15219. 28537261 10.1038/ncomms15219 PMC5529674

[143] C. Fu , Research (Wash D C) 2020, 2020, 4643507. 32318686 10.34133/2020/4643507 PMC7166253

[144] M. Neupane , Nat. Commun. 2014, 5, 3786. 24807399 10.1038/ncomms4786

[145] T. Liang , J. Lin , Q. Gibson , T. Gao , M. Hirschberger , M. Liu , R. J. Cava , N. P. Ong , Phys. Rev. Lett. 2017, 118, 136601. 28409962 10.1103/PhysRevLett.118.136601

[146] S. Yue , H. T. Chorsi , M. Goyal , T. Schumann , R. Yang , T. Xu , B. Deng , S. Stemmer , J. A. Schuller , B. Liao , Phys. Rev. Res. 2019, 1, 033101.

[147] C. Zhang , T. Zhou , S. Liang , J. Cao , X. Yuan , Y. Liu , Y. Shen , Q. Wang , J. Zhao , Z. Yang , F. Xiu , Chin. Phys. B 2015, 25, 017202.

[148] Z. Gui , Y. Yang , X. Wen , Y. Zhang , Y. Li , Y. Li , Q. Liu , M. Wang , J. Ying , X. Chen , Energy Environ. Sci. 2024, 17, 7129.

[149] B. Lenoir , M. Cassart , J. P. Michenaud , H. Scherrer , S. Scherrer , J. Phys. Chem. Solids 1996, 57, 89.

[150] D.‐Y. Chung , T. Hogan , P. Brazis , M. Rocci‐Lane , C. Kannewurf , M. Bastea , C. Uher , M. G. Kanatzidis , Science 2000, 287, 1024. 10669411 10.1126/science.287.5455.1024

[151] J. Xiang , H. SiLe , L. Meng , Z. WenLiang , M. ChaoYang , C. ZiYu , S. Frank , C. GenFu , S. PeiJie , Sci. China Phys., Mech. Astronomy. 2020, 63, 1.

[152] P. Wang , C.‐w. Cho , F. Tang , P. Wang , W. Zhang , M. He , G. Gu , X. Wu , Y. Shao , L. Zhang , Phys. Rev. B 2021, 103, 045203.

[153] J. Hu , S.‐Y. Xu , N. Ni , Z. Mao , Annu. Rev. Mater. Res. 2019, 49, 207.

[154] S. J. Watzman , T. M. McCormick , C. Shekhar , S.‐C. Wu , Y. Sun , A. Prakash , C. Felser , N. Trivedi , J. P. Heremans , Phys. Rev. B 2018, 97.

[155] Z. Zhu , Phys. Rev. Lett. 2015, 114, 176601. 25978245 10.1103/PhysRevLett.114.176601

[156] K. Behnia , M. A. Measson , Y. Kopelevich , Phys. Rev. Lett. 2007, 98, 076603. 17359042 10.1103/PhysRevLett.98.076603

[157] P. Li , P. Qiu , Q. Xu , J. Luo , Y. Xiong , J. Xiao , N. Aryal , Q. Li , L. Chen , X. Shi , Nat. Commun. 2022, 13, 7612. 36494353 10.1038/s41467-022-35289-z PMC9734562

[158] P. Li , P. Qiu , J. Xiao , T. Deng , L. Chen , X. Shi , Energy Environ. Sci. 2023, 16, 3753.

[159] T. Feng , P. Wang , Z. Han , L. Zhou , Z. Wang , W. Zhang , Q. Liu , W. Liu , Energy Environ. Sci. 2023, 16, 1560.

[160] T. Feng , Adv. Mater. 2022, 34, 2200931.

[161] H. Wang , Z. Zhou , J. Ying , Z. Xiang , R. Wang , A. Wang , Y. Chai , M. He , X. Lu , G. Han , Y. Pan , G. Wang , X. Zhou , X. Chen , Adv. Mater. 2023, 35, 2206941. 10.1002/adma.202206941 36300801

[162] Z. Chen , X. Zhang , J. Ren , Z. Zeng , Y. Chen , J. He , L. Chen , Y. Pei , Nat. Commun. 2021, 12, 3837. 34158499 10.1038/s41467-021-24161-1 PMC8219662

[163] A. M. Ochs , G. H. Fecher , B. He , W. Schnelle , C. Felser , J. P. Heremans , J. E. Goldberger , Adv. Mater. 2023, 36, 2308151. 10.1002/adma.202308151 37853575

[164] S. Wu , X. Wang , X. Mi , S. Zheng , K. Yang , Z. Zhou , H. Wang , G. Han , X. Lu , Y. Pan , G. Wang , X. Zhou , Adv. Energy Mater. 2024, 14, 2400184.

[165] K. Tsuruda , K. Nakagawa , M. Ochi , K. Kuroki , M. Tokunaga , H. Murakawa , N. Hanasaki , H. Sakai , Adv. Funct. Mater. 2021, 31, 2102275.

[166] Y. Pan , B. He , T. Helm , D. Chen , W. Schnelle , C. Felser , Nat. Commun. 2022, 13, 3909. 35798731 10.1038/s41467-022-31372-7 PMC9262886

[167] S. R. Boona , H. Jin , S. Watzman , J. Appl. Phys. 2021, 130, 171101.

[168] P. Jandl , U. Birkholz , J. Appl. Phys. 1994, 76, 7351.

[169] D. Xiao , Y. Yao , Z. Fang , Q. Niu , Phys. Rev. Lett. 2006, 97, 026603. 16907470 10.1103/PhysRevLett.97.026603

[170] G. Sharma , P. Goswami , S. Tewari , Phys. Rev. B 2016, 93, 035116.

[171] F. Caglieris , C. Wuttke , S. Sykora , V. Süss , C. Shekhar , C. Felser , B. Büchner , C. Hess , Phys. Rev. B 2018, 98, 201107.

[172] J. Hu , M. Caputo , E. B. Guedes , S. Tu , E. Martino , A. Magrez , H. Berger , J. H. Dil , H. Yu , J.‐P. Ansermet , Phys. Rev. B 2019, 100, 115201.

[173] T. Chen , T. Tomita , S. Minami , M. Fu , T. Koretsune , M. Kitatani , I. Muhammad , D. Nishio‐Hamane , R. Ishii , F. Ishii , R. Arita , S. Nakatsuji , Nat. Commun. 2021, 12, 572. 33495448 10.1038/s41467-020-20838-1 PMC7835387

[174] S. Nakatsuji , R. Arita , Annu. Rev. Condens. Matter Phys. 2022, 13, 119.

[175] W.‐L. Lee , S. Watauchi , V. Miller , R. Cava , N. Ong , Phys. Rev. Lett. 2004, 93, 226601. 15601108 10.1103/PhysRevLett.93.226601

[176] K.‐i. Uchida , W. Zhou , Y. Sakuraba , Appl. Phys. Lett. 2021, 118, 140504.

[177] Y. Pan , C. Le , B. He , S. J. Watzman , M. Yao , J. Gooth , J. P. Heremans , Y. Sun , C. Felser , Nat. Mater. 2022, 21, 203. 34811495 10.1038/s41563-021-01149-2 PMC8810386

[178] P. Wang , Z. Hu , X. Wu , Q. Liu , npj Comput. Mater. 2023, 9, 203.

[179] A. Sakai , S. Minami , T. Koretsune , T. Chen , T. Higo , Y. Wang , T. Nomoto , M. Hirayama , S. Miwa , D. Nishio‐Hamane , F. Ishii , R. Arita , S. Nakatsuji , Nature 2020, 581, 53. 32376952 10.1038/s41586-020-2230-z

[180] A. Sakai , Y. P. Mizuta , A. A. Nugroho , R. Sihombing , T. Koretsune , M.‐T. Suzuki , N. Takemori , R. Ishii , D. Nishio‐Hamane , R. Arita , P. Goswami , S. Nakatsuji , Nat. Phys. 2018, 14, 1119.

[181] H. Reichlova , R. Schlitz , S. Beckert , P. Swekis , A. Markou , Y.‐C. Chen , D. Kriegner , S. Fabretti , G. Hyeon Park , A. Niemann , S. Sudheendra , A. Thomas , K. Nielsch , C. Felser , S. T. B. Goennenwein , Appl. Phys. Lett. 2018, 113, 212405.

[182] F. Mende , J. Noky , S. N. Guin , G. H. Fecher , K. Manna , P. Adler , W. Schnelle , Y. Sun , C. Fu , C. Felser , Adv. Sci. 2021, 8, 2100782. 10.1002/advs.202100782 PMC8425906 34240573

[183] S. N. Guin , P. Vir , Y. Zhang , N. Kumar , S. J. Watzman , C. Fu , E. Liu , K. Manna , W. Schnelle , J. Gooth , C. Shekhar , Y. Sun , C. Felser , Adv. Mater. 2019, 31, 1806622. 10.1002/adma.201806622 31044469

[184] L. Ding , J. Koo , L. Xu , X. Li , X. Lu , L. Zhao , Q. Wang , Q. Yin , H. Lei , B. Yan , Z. Zhu , K. Behnia , Phys. Rev. X 2019, 9, 041061.

[185] D. Khadka , Phys. Rev. Mater. 2020, 4, 084203.

[186] H. Zhang , J. Koo , C. Xu , M. Sretenovic , B. Yan , X. Ke , Nat. Commun. 2022, 13, 1091. 35232990 10.1038/s41467-022-28733-7 PMC8888656

[187] S. Roychowdhury , A. M. Ochs , S. N. Guin , K. Samanta , J. Noky , C. Shekhar , M. G. Vergniory , J. E. Goldberger , C. Felser , Adv. Mater. 2022, 34, 2201350. 10.1002/adma.202201350 35980946

[188] X. Zhou , H. Liu , W. Wu , K. Jiang , Y. Shi , Z. Li , Y. Sui , J. Hu , J. Luo , Phys. Rev. B 2022, 105, 205104.

[189] T. Chen , S. Minami , A. Sakai , Y. Wang , Z. Feng , T. Nomoto , M. Hirayama , R. Ishii , T. Koretsune , R. Arita , S. Nakatsuji , Sci. Adv. 2022, 8, abk1480. 10.1126/sciadv.abk1480 PMC8759748 35030028

[190] B. He , C. Sahin , S. R. Boona , B. C. Sales , Y. Pan , C. Felser , M. E. Flatté , J. P. Heremans , Joule 2021, 5, 3057. 34841198 10.1016/j.joule.2021.08.007 PMC8604385

[191] C. Ran , X. Mi , J. Shen , H. Wang , K. Yang , Y. Liu , G. Wang , G. Wang , Y. Shi , A. Wang , Y. Chai , X. Yang , M. He , X. Tong , X. Zhou , Phys. Rev. B 2023, 108, 125103.

[192] M. Ikhlas , T. Tomita , T. Koretsune , M.‐T. Suzuki , D. Nishio‐Hamane , R. Arita , Y. Otani , S. Nakatsuji , Nat. Phys. 2017, 13, 1085.

[193] X. Li , L. Xu , L. Ding , J. Wang , M. Shen , X. Lu , Z. Zhu , K. Behnia , Phys. Rev. Lett. 2017, 119, 056601. 28949739 10.1103/PhysRevLett.119.056601

[194] C. Wuttke , F. Caglieris , S. Sykora , F. Scaravaggi , A. U. B. Wolter , K. Manna , V. Süss , C. Shekhar , C. Felser , B. Büchner , C. Hess , Phys. Rev. B 2019, 100, 085111.

[195] Y. Shiomi , N. Kanazawa , K. Shibata , Y. Onose , Y. Tokura , Phys. Rev. B 2013, 88, 064409.

[196] M. Hirschberger , L. Spitz , T. Nomoto , T. Kurumaji , S. Gao , J. Masell , T. Nakajima , A. Kikkawa , Y. Yamasaki , H. Sagayama , H. Nakao , Y. Taguchi , R. Arita , T.‐H. Arima , Y. Tokura , Phys. Rev. Lett. 2020, 125, 076602. 32857583 10.1103/PhysRevLett.125.076602

[197] H. Zhang , C. Xu , X. Ke , Phys. Rev. B 2021, 103, L201101.

[198] Y. Tokura , N. Kanazawa , Chem. Rev. 2021, 121, 2857. 33164494 10.1021/acs.chemrev.0c00297

[199] X. Z. Yu , W. Koshibae , Y. Tokunaga , K. Shibata , Y. Taguchi , N. Nagaosa , Y. Tokura , Nature 2018, 564, 95. 30518889 10.1038/s41586-018-0745-3

[200] H. Zhang , C. Q. Xu , X. Ke , Phys. Rev. B 2021, 103, L201101.

[201] K. K. Kolincio , M. Hirschberger , J. Masell , S. Gao , A. Kikkawa , Y. Taguchi , T. H. Arima , N. Nagaosa , Y. Tokura , Proc. Natl. Acad. Sci. USA 2021, 10.1073/pnas.2023588118. PMC8379910 34389668

[202] M. Mizuguchi , S. Nakatsuji , Sci. Technol. Adv. Mater. 2019, 20, 262. 30956732 10.1080/14686996.2019.1585143 PMC6442159

[203] M. Murata , K. Nagase , K. Aoyama , A. Yamamoto , Y. Sakuraba , iScience 2021, 24, 101967. 33458616 10.1016/j.isci.2020.101967 PMC7797944

[204] Y. Sakuraba , Scr. Mater. 2016, 111, 29.

[205] W. Zhou , Y. Sakuraba , Appl. Phys. Express 2020, 13, 043001.

[206] X. Li , Z. Zhu , K. Behnia , Adv. Mater. 2021, 33, 2100751. 10.1002/adma.202100751 33844874

[207] H. Tanaka , T. Higo , R. Uesugi , K. Yamagata , Y. Nakanishi , H. Machinaga , S. Nakatsuji , Adv. Mater. 2023, 35, 2303416. 10.1002/adma.202303416 37343181

[208] S. Noguchi , K. Fujiwara , Y. Yanagi , M.‐T. Suzuki , T. Hirai , T. Seki , K.‐I. Uchida , A. Tsukazaki , Nat. Phys. 2024, 20, 254.

[209] A. Ziabari , M. Zebarjadi , D. Vashaee , A. Shakouri , Rep. Prog. Phys. 2016, 79, 095901. 27519021 10.1088/0034-4885/79/9/095901

[210] W. Zhou , K. Yamamoto , A. Miura , R. Iguchi , Y. Miura , K.‐I. Uchida , Y. Sakuraba , Nat. Mater. 2021, 20, 463. 33462463 10.1038/s41563-020-00884-2

[211] M. Figueira , J. Silva‐Valencia , R. Franco , The Eur. Phys. J. B 2012, 85, 1.

[212] Y. Zhang , M. S. Dresselhaus , Y. Shi , Z. Ren , G. Chen , Nano Lett. 2011, 11, 1166. 21280642 10.1021/nl104090j

[213] M. Koirala , H. Wang , M. Pokharel , Y. Lan , C. Guo , C. Opeil , Z. Ren , Nano Lett. 2014, 14, 5016. 25079115 10.1021/nl501436w

[214] K. Wei , J. N. Neu , Y. Lai , K. W. Chen , D. Hobbis , G. S. Nolas , D. E. Graf , T. Siegrist , R. E. Baumbach , Sci. Adv. 2019, 5, aaw6183. 10.1126/sciadv.aaw6183 PMC6544447 31172031

[215] D. Rowe , V. Kuznetsov , L. Kuznetsova , G. Min , J. Phys. D: Appl. Phys. 2002, 35, 2183.

[216] R. Wolfe , G. Smith , Appl. Phys. Lett. 1962, 1, 5.

[217] J. P. Heremans , In Tri‐Technology Device Refrigeration (TTDR), International Society for Optics and Photonics, Bellingham, Washington, USA 2016, 98210G.

[218] K. Scholz , P. Jandl , U. Birkholz , Z. Dashevskii , J. Appl. Phys. 1994, 75, 5406.

